# In Silico Design of a Chimeric Humanized L-asparaginase

**DOI:** 10.3390/ijms24087550

**Published:** 2023-04-20

**Authors:** Alejandro Pedroso, Lisandra Herrera Belén, Jorge F. Beltrán, Rodrigo L. Castillo, Adalberto Pessoa, Enrique Pedroso, Jorge G. Farías

**Affiliations:** 1 Department of Chemical Engineering, Faculty of Engineering and Science, Universidad de La Frontera, Temuco 4811230, Chile; 2 Departamento de Ciencias Básicas, Facultad de Ciencias, Universidad Santo Tomas, Avenida Carlos Schorr 255, Talca 3460000, Chile; 3 Department of Internal Medicine, East Division, Faculty of Medicine, University of Chile, Santiago 7500922, Chile; 4 Department of Biochemical and Pharmaceutical Technology, School of Pharmaceutical Sciences, University of São Paulo, São Paulo 05508-000, Brazil; 5 Department of Family Medicine, Faculty of Medicine, University of Medical Sciences Matanzas, Matanzas 42300, Cuba

**Keywords:** L-asparaginase, chimeric, in silico, immunogenicity, acute lymphoblastic leukemia

## Abstract

Acute lymphoblastic leukemia (ALL) is the most common cancer among children worldwide, characterized by an overproduction of undifferentiated lymphoblasts in the bone marrow. The treatment of choice for this disease is the enzyme L-asparaginase (ASNase) from bacterial sources. ASNase hydrolyzes circulating L-asparagine in plasma, leading to starvation of leukemic cells. The ASNase formulations of *E. coli* and *E. chrysanthemi* present notorious adverse effects, especially the immunogenicity they generate, which undermine both their effectiveness as drugs and patient safety. In this study, we developed a humanized chimeric enzyme from *E. coli* L-asparaginase which would reduce the immunological problems associated with current L-asparaginase therapy. For these, the immunogenic epitopes of *E. coli* L-asparaginase (PDB: 3ECA) were determined and replaced with those of the less immunogenic Homo sapiens asparaginase (PDB:4O0H). The structures were modeled using the Pymol software and the chimeric enzyme was modeled using the SWISS-MODEL service. A humanized chimeric enzyme with four subunits similar to the template structure was obtained, and the presence of asparaginase enzymatic activity was predicted by protein–ligand docking.

## 1. Introduction

Acute lymphoblastic leukemia (ALL) is the most frequent neoplasm in childhood, constituting 80% of all acute leukemias in the pediatric age group. This is a hematologic neoplasm, the lymphoblastic precursors of which proliferate rapidly and replace other hematopoietic cells. Hence, 25% and 19% of all tumors in children under 15 and 19 years of age, respectively, are ALL [1].

Among the drugs used for chemotherapeutic treatment of childhood acute lymphoblastic leukemia (ALL) is L-asparaginase (EC number 3.5.1.1), an enzyme that has successfully contributed to increased survival rates for this disease [2,3]. L-asparaginase is the first therapeutic enzyme with antineoplastic properties extensively studied by researchers and scientists worldwide. The antitumor properties of L-asparaginase are due to its hydrolytic capacity. This enzyme, belonging to the group of amidase enzymes, can decompose the amino acid L-asparagine into aspartate and ammonia and also presents glutaminase activity [4]. Asparagine is a non-essential amino acid necessary for protein synthesis and normal cell growth [5]. Although healthy cells can synthesize asparagine from aspartic acid through the enzyme asparagine synthetase, neoplastic cells depend on an exogenous supply of asparagine for their existence and reproduction since they do not possess the intracellular capacity to synthesize enough asparagine [5,6]. Based on this, the use of L-asparaginase in chemotherapies was implemented, and it has been demonstrated that in tumor cells, the hydrolysis of asparagine by L-asparaginase drains all circulating asparagine, resulting in the depletion of the serum asparagine, leading to starvation of the leukemic cells [5]. This leads to starvation of cancer cells and ultimately to DNA breaks, cell cycle arrest, and apoptosis [7,8]. As a result of these observations, L-asparaginase-containing therapies have been developed to treat hematologic malignancies including acute lymphoblastic leukemia (ALL). Native ASNases derived from *Escherichia coli* [9], a recombinant preparation of ASNase from *E. coli* [10], and two PEGylated forms of ASNase from native *E. coli* [11,12], are available. The ASNase enzyme isolated from *Erwinia chrysanthemi* [13] and a recombinant version of it are also available [14].

Due, in part, to their bacterial origin and large size, the clinically used L-asparaginases are a notable example of a well-established therapeutic protein with the ability to elicit an immune response in patients [15]. Problems associated with immunodeficiency and acute liver dysfunction are the main side effects of ASNase in leukemia therapy [16]. When L-asparaginase is administered to patients, both normal and leukemic lymphoblasts can cleave it via the lysosomal proteases cathepsin B and asparagine endopeptidase, enhancing antigenic antigen processing [17] and promoting an immune response [18,19,20]. This immune response is the leading cause of discontinuation of L-asparaginase treatment and is known as hypersensitivity [21]. Adverse reactions caused by hypersensitivity include anaphylaxis, edema, serum sickness, bronchospasm, urticaria and exanthema, pruritus and swelling of the extremities, and erythema [18]. Currently, between 30% and 75% of patients exhibit some form of hypersensitivity and up to 70% develop antidrug antibodies [22]. Additionally, the production of anti-L-asparaginase antibodies is known to alter the pharmacokinetics and clearance of the drug, which may be the cause of reported cases of reduced drug potency [23,24,25,26]. This induces a condition called subclinical hypersensitivity or silent inactivation in which the efficacy of the drug is reduced or eliminated and the patient is asymptomatic, resulting in poor therapeutic outcomes [5,27]. In general, this is a major disadvantage of using bacterial enzymes as therapeutic agents.

Several solutions have been explored to minimize the immunogenicity of bacterial asparaginase. These strategies include PEGylation, protein engineering, new asparaginase sources, and N-glycosylation, among others [28,29,30,31,32,33,34].

Previous work involving the production of chimeric proteins has also been developed in association with ASNase. In 1995, Newsted et al. demonstrated that the resistance of L-asparaginase to proteolytic degradation by trypsin can be increased by developing a chimera comprising a fusion of the ASNase gene with that of a single-strand antibody derived from a pre-selected monoclonal antibody capable of providing protection against trypsin [35]. Engineered chimeric L-asparaginase retained 75% of its original activity after exposure to trypsin while the native L-asparaginase control was completely inactivated [35]. In 2006, Qi Gaofu et al. developed a chimeric variant comprising asparaginase, tetanus toxin helper T-cell epitope, and B-cell epitope of human cholesteryl ester transfer protein (CETP) [36]. The chimeric enzyme was expressed as a soluble protein in *Escherichia coli* and, once purified, exhibited approximately 83% of native asparaginase activity [36]. With the bioinformatics boom, computational tools used to resolve protein immunogenicity problems have garnered increasing interest. It has been shown that de-immunogenization of therapeutic proteins by modifying T-cell or B-cell epitopes using bioinformatic tools and site-directed mutagenesis is a successful strategy for obtaining safer biopharmaceutical products [16,37,38]. Based on these ideas and on the high immunogenicity of *E. coli* asparaginase in 2021, Belén et al. developed a chimera of L-asparaginase in silico by replacing epitope peptides in the *E. coli* enzyme variant with peptides from human serum albumin and even demonstrated proof of concept that the engineered variant is recombinantly expressed in *E. coli* (paper accepted for publication).

Native-type variants of known human L-asparaginases are unsuitable replacements for clinically used bacterial enzymes as they have a very high KM value for Asn. Given the physiological blood concentration of Asn (~50 μM), the enzyme must have an Asn KM in the low micromolar range to be clinically relevant [39,40,41]. However, this does not restrict the use of its fragments in epitope substitution in enzyme variants of other species to limit the development of an immune response in therapeutic treatments.

On this basis, this research proposal developed a humanized chimeric enzyme in silico from *E. coli* L-asparaginase. The in-silico detection of potentially antigenic and allergenic epitopes in bacterial ASNase and the substitution of these epitopes by peptide fragments of human ASNase with similar secondary structures were carried out without affecting the active center, thereby avoiding the development of an immune response and obtaining a chemically and functionally stable in silico chimeric protein.

## 2. Results and Discussion

### 2.1. Determination of Epitope Density

Epitope density is defined as an alternative measure to estimate the degree of response of the immune system. It consists of a comparative analysis of the number of epitopes of two different proteins to predict which one is more immunogenic. For example, if you have two proteins X and Y, if protein X has many more epitopes than protein Y, then it can be estimated that protein X will be more immunogenic than protein Y [42,43,44].

The study of pathogens has benefitted from the calculation of the density of epitopes presented by MHC I and II since this method can determine how, using mutations and epitope regulations introduced in these histocompatibility complexes, bacteria and viruses evade recognition by the immune system [45,46,47].

This method has also been widely used in the study of the degree of immunogenicity of many proteins for therapeutic purposes. Hence, in this study, the same concept of optical density was applied to evaluate the degree of immunogenicity of the *E. coli* L-asparaginase enzyme by calculating the relative frequency of the predicted immunogenic peptides. The aim was to demonstrate the high immunogenicity of the *E. coli* enzyme. The results showed the existence of 532 immunogenic peptides with a total relative frequency of 0.24 for a number of epitopes amounting to 2205 according to the peptide fragmentation that was designed (9 to 15 aa), indicating that about one quarter of the total peptides into which the 3ECA protein was fragmented can induce an immune response by generating the adverse reaction to the treatment (Supplementary Table S1). This is in line with previous studies where the *E. coli* enzyme was compared with ErA which found that both enzymes present a high density of immunogenic epitopes homogeneously distributed in their structures [37]. This finding was justified based on the high sequence similarity of both bacterial enzymes [37,48].

Additionally, an allergenicity analysis was performed to identify the antigenic peptides analyzed from the HLA-DRB1*04:01 and HLA-DRB1*07:01 allele (Supplementary Table S1). A total of 120 allergenic peptides were discovered, of which 49 correspond to the HLA-DRB1*04:01 allele and the remaining 71 to the HLA-DRB1*07:01 allele. The results obtained are similar to those reported by Belen in 2019 [49]. These peptides have the highest capacity to induce a more potent immune response due to their antigenic and allergenic nature and are therefore favorable candidates to be considered for substitution in the design of a chimeric enzyme.

On the other hand, once the human and bacterial variants 4O0H and 3ECA were coupled, it was found that the human variant presented 19.84% of sequence identity with 3 ECA due to the evolutionary distance between bacteria and humans. However, both enzymes have the same functionality, which points to the existence of a common ancestor. Moreover, since they present the same enzymatic function, there are structural residues that must be conserved and be common not only in these enzymes but in all those that belong to the same family to conserve the function of degrading asparagine [38,50,51]. Since human L-asparaginases, unlike the *E. coli*-derived one, possess a KM value outside the micromolar range and are therefore not clinically relevant, they are not suitable substitutes for clinically used bacterial enzymes [40]. However, their human origin prevents patients from developing hypersensitivity to the treatment [40,41].

The deimmunization of proteins for therapeutic use by substituting potentially immunogenic epitopes of both T-cells and B-cells is a successful strategy in developing safer biopharmaceuticals. In this case, *E. coli* L-asparaginase has high immunogenicity, as demonstrated in ongoing research and preceding studies; therefore, obtaining fewer immunogenic mutated variants of the enzyme through different pathways is a step forward in the use of this enzyme as a chemotherapeutic drug. Despite the low similarity between the human enzyme and that of bacterial origin, the human enzyme is an excellent candidate for peptide substitution in obtaining a less immunogenic humanized chimeric enzyme since it makes the organism non-reactive to the selected fragments, thus lowering the threshold of the immune response to the use of a humanized chimera.

### 2.2. Mapping and Structure Determination of the Chimeric Enzyme

The in-silico design of the humanized chimeric enzyme included the substitution of peptides other than the native 3ECA protein. In the present study, two peptides were substituted from the native enzyme with a size of 15 amino acids each and an affinity value for MHC II lower than 4 in a range from 0 to 10, indicating recognition and high affinity by MHC II toward these peptides from the native protein [37,52]. This is an important indicator to consider when substituting fragments of the bacterial enzyme for fragments of human proteins as it is a marker of how strongly the epitopes bind to MHC II and therefore indicates a favorable substitution to reduce immunogenicity [52].

There is a marked incidence of patients treated with L-asparaginase experiencing hypersensitivity reactions at a rate of 25% to 30% [53], which is why the number of immunogenic epitopes with allergenic capacity present in the structure of the 3ECA enzyme was considered in the development of the humanized chimeric enzyme. As a result, a high density of allergenic epitopes was obtained in the native protein, which is consistent with some studies that suggest that the bacterial enzyme from *E. coli* develops a greater hypersensitivity reaction than other enzymes of bacterial origin [29,37,54]. Several combinations of peptides were tested according to the alleles studied and reported as more immunogenic. The most accepted combination correspond to the VENLVNAVPQLKDIA peptide, which was reported as immunogenic by the HLA-DRB1*08:01 allele, and the DGPFNLYNAVVVTAAD peptide, which was reported as immunogenic by the HLA-DRB1*01:01, HLA-DRB1*07:01, and HLA-DRB1*15:01 alleles. Previous studies indicate that the HLA-DRB1*07:01 allele is associated with a high risk of hypersensitivity in patients with acute lymphoblastic leukemia treated with *E. coli* asparaginase [23,37,38]. For the case of the HLA-DRB1*15:01 allele, this peptide was also observed to bind strongly to MHC II. Taking this as background, it was decided that this conjugation of the two peptides would be the ideal one to substitute in the design of the chimeric enzyme to decrease the allergenic load of the chimeric protein. The substitution of the DGPFNLYNAVVTAAD peptide for peptide analogs of the human protein caused a decrease of 14 immunogenic epitopes in the HLA-DRB1*07:01 allele in the chimeric enzyme compared to the native 3ECA, which increases the feasibility of using this enzyme in the treatment of patients with ALL by decreasing the probability of developing hypersensitivity with treatment associated with this specific allele.

**Figure 1:**
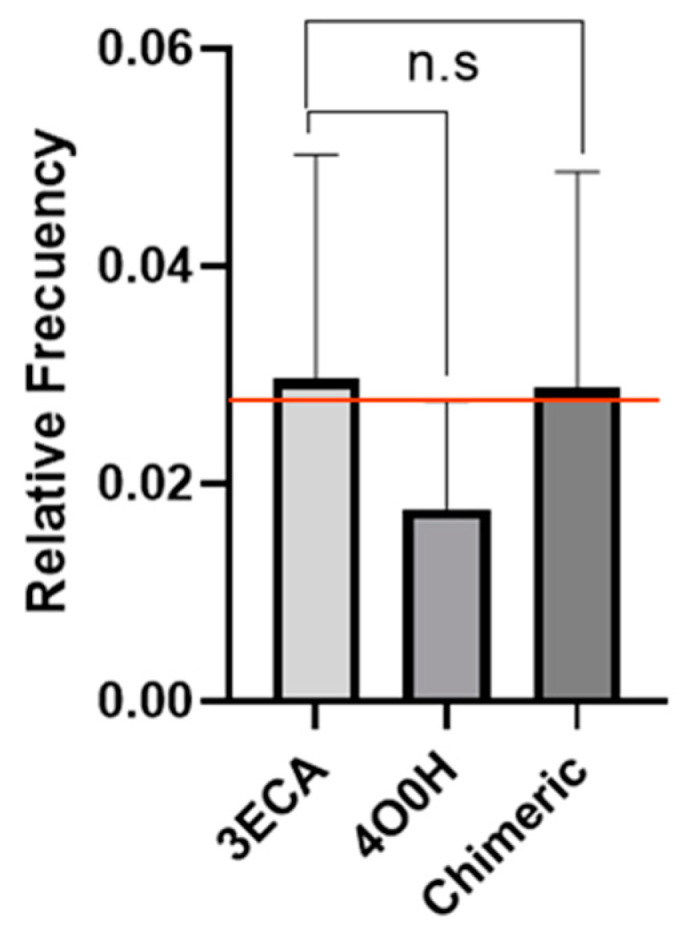
Relative frequency of T-cell immunogenic epitopes for 3ECA, 4O0H proteins, and humanized 3ECA chimeric protein (Chimeric). Statistical analysis of the relative frequency of predicted T-cell immunogenic epitopes for the chimeric enzyme according to the number of immunogenic peptides of HLA-DRB1*01 alleles: 01, HLA-DRB1*03:01, HLA-DRB1*04:01, HLA-DRB1*07:01, HLADRB1*08:01, HLA-DRB1*11:01, HLA-DRB1*13:01, and HLADRB1*15:01, which are reference alleles in the literature with a wide global frequency. The results showed no significant differences between the relative frequencies calculated for both enzymes, with a probability of *p* = 0.075. The red line shows the slight numerical decrease between the native and chimeric enzymes. To compare the epitope density of the enzymes, a Tukey test (*p* < 0.05) was performed with the mean epitope densities using the statistical package GraphPad Prism version 5.0 for Windows (GraphPad Software, San Diego, CA, USA).

When the overall antigenicity of the chimeric enzyme and the native enzyme were compared, the chimeric enzyme was slightly lower than the native enzyme (Figure 1). This decrease is not statistically significant and is only reflected in a difference of 14 fewer immunogenic peptides in the chimeric enzyme than the native enzyme. When analyzing this result, it should be considered that this program only considers the protein sequence as a monomer, i.e., it calculates the immunogenicity of the supplied sequence independently of its protein structure. The asparaginase enzyme in its native folded conformation presents a homotetrameric structure, i.e., the sequence is folded and repeated four times to form a homotetramer and thus conform to the three-dimensional structure of the protein [55]. Nevertheless, the fact that there is a decrease in the immunogenicity of the chimeric enzyme compared to the native one in this preliminary in silico study may represent a favorable advance for in vitro studies where cellular dynamism may magnify the positive effect desired with the design of this chimera, which may be a step forward in the de-immunogenization of asparaginase.

**Figure 2:**
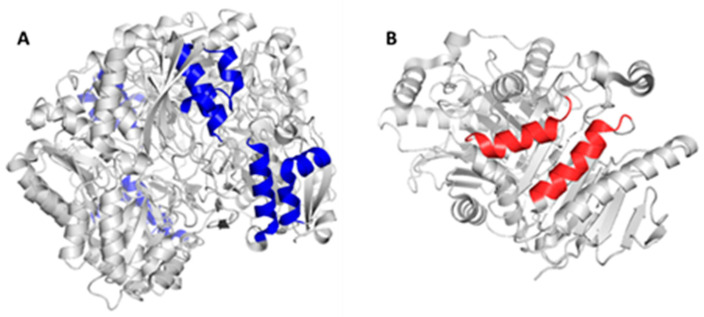
Substituted sequences for the construction of the chimeric *E. coli* L-asparaginase enzyme. (**A**) Tetrameric structure of L-asparaginase II from *E. coli* 3ECA; the residues to be substituted by peptide fragments of the human enzyme are indicated in blue. (**B**) Dimeric structure of human L-asparaginase (4O0H); the fragments of the human enzyme that align to the bacterial enzyme and that were used as substituents in the formation of the chimeric construct are indicated in red. Both fragments have the same secondary structure.

Considering the structural characteristics of the designed chimera protein, it was observed that the substituent polypeptide chains retain the same secondary structure in the form of an alpha helix as the fragments of the native 3ECA mold protein (Figure 2).

It is known that the secondary structure of proteins is the folding that the polypeptide chain adopts thanks to the formation of hydrogen bridges between the atoms that form the peptide bond. Hydrogen bridges are established between the CO and NH groups of the peptide bond (the former as an H acceptor and the latter as an H donor). In this way, the polypeptide chain can adopt conformations with lower free energy and is therefore more stable [56]. It is therefore essential for the peptide substituents of the chimeric enzyme to retain this secondary structure as the stability and functionality of the enzyme may otherwise be compromised.

**Figure 3:**
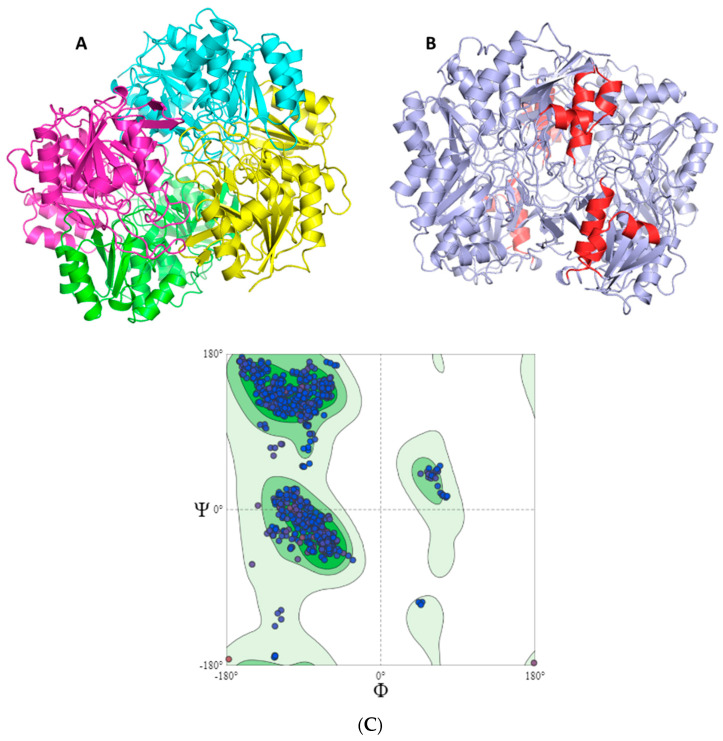
Humanized chimeric *E. coli* L-asparaginase with fragments of human L-asparaginase 4O0H and evaluation of the accuracy of the predicted protein structure. (**A**) The humanized 3ECA-derived chimeric enzyme is shown in tetrameric conformation with four independently folded subunits. Subunit A is shown in green, subunit B in cyan, subunit C in magenta, and subunit D in yellow. (**B**) The humanized 3ECA-derived chimeric enzyme is shown; the fragments of human L-asparaginase 4O0H that were substituted are shown in red. (**C**) Ramachandran diagram for the chimeric L-asparaginase protein. Observed values of the Φ and Ψ angles (blue point) for all humanized 3ECA-derived amino acids in the chimeric protein. In dark green are presented the allowed and favorable zones. In light green, the less favorable zones are presented and in lighter green and white, the most unstable and prohibited areas, respectively, where the combinations of the Φ and Ψ angles are not allowed by steric hindrance. In the first quadrant, the left α-helix combinations are represented, in the second quadrant are the β-sheet combinations, and in the third quadrant are the right α-helices and loops. The chimeric protein was characterized by a stable conformation with most of the amino acids in the allowed zone. It was characterized by a predominance of right α-helices and β-sheets.

Morphologically, the chimeric protein obtained was characterized by four folded subunits that are distinguishable at a glance (Figure 3A,B). This coincides with what is reported in the literature for *E. coli* L-asparaginase (3ECA), which is shown in its active conformation as a tetramer composed of four identical subunits—A, B, C, and D—with an approximate symmetry of 222 [55,57]. The chimeric protein obtained by homology modeling was characterized as presenting stability and a correct arrangement of the three-dimensional structure. The results obtained in the Ramachandran plot were 96.06% favorable zones (Figure 3C). The result obtained for the present chimeric protein demonstrates that most of the residues in the protein fall within the permitted areas of the diagram, especially in the regions corresponding to the β-sheets and α-helices. There are also some residues that fall in less common regions, encompassing even the area of the type II turns. However, there is a small fraction of residues that appears in forbidden areas of the diagram, which indicates that the three-dimensional structure available to us is not perfect and presents some residues in positions where they are forced so it is unlikely that they are their real locations, but they do not limit or affect the stability of the protein.

**Figure 4:**
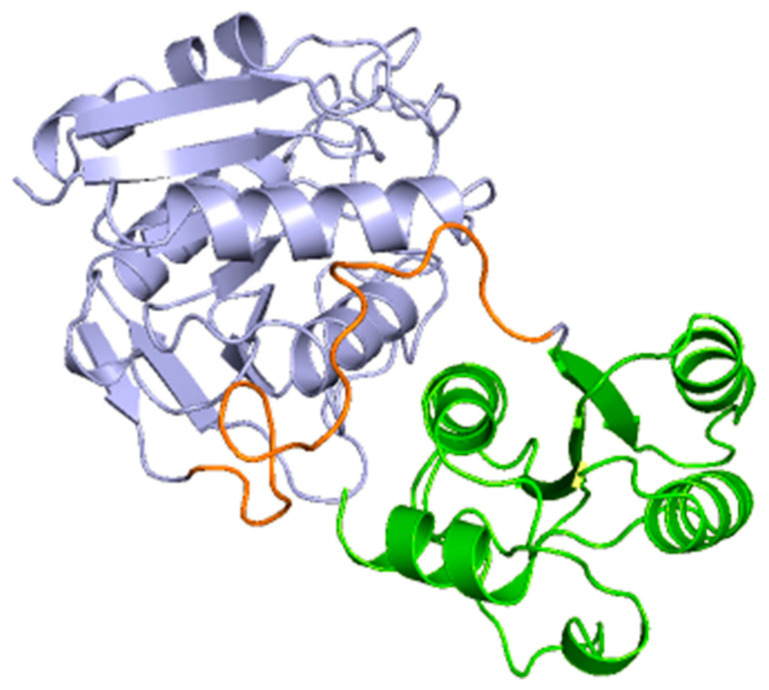
Subunit of chimeric humanized *E. coli* L-asparaginase with fragments of human L-asparaginase 4O0H. Subunit of the chimeric humanized 3ECA-derived chimeric enzyme is shown in independently folded three-dimensional conformation. The C-terminal end of the subunit with four α-helices and one parallel β-sheet is shown in green. The loop separating the two α/β domains formed by the subunit is shown in orange.

In the subunits of the obtained chimeric enzyme, it can be estimated that a single subunit is composed of two polypeptide clusters with a wide density of amino acids that fold independently, forming loops and turns, beta sheets, and alpha helices. In addition, it is observed that these dense populations of amino acids are connected by a loop extending from amino acid 190 to approximately 213. Although this analysis is inconclusive, it provides us with an approximation of how the protein structure is maintained in the face of the imposed modifications (Figure 4).

This analysis is consistent with the structure described for the basic subunit of the enzyme 3ECA, which distributes its amino acid sequence into two α/β domains connected by a linker sequence (191 aa-212 aa) [55]. Additionally, for the C-terminal end of the protein subunit of the bacterial enzyme, the existence of four alpha helices and a beta sheet composed of three parallel strands has been shown [55]. These characteristics are common to the subunit of the modeled chimeric enzyme as shown in Figure 4; therefore, it can be inferred that both proteins present the same folding and three-dimensional structures and that they only differ in their amino acid sequences.

Since the predicted chimeric protein model was made using the SWISS-MODEL server, which is based on homology modeling, it was decided to model the protein using AlphaFold2 in order to make the structure more solid. This program takes information from the amino acid sequence of the chimeric protein and compares it with known structures determined by laboratory techniques. Thus, by means of an algorithm, it generates a data matrix that calculates the probability that two amino acids can be close in a protein [58]. Using this data and machine learning, the system repeatedly tests multiple possible structures, thus evaluating the most likely three-dimensional conformation the amino acid chain of the chimeric protein will adopt [58].

From the AlphaFold2 modeling, the chimeric protein dimer BD was obtained, which exhibited the presence of two α/β domains connected by a loop that serves as a linking sequence between the two. This corresponds to the same structural features as the SWISS-MODEL monomer and are in line with the native 3ECA protein reported in the PDB database [55]. For a better understanding of the results, the similarity between the two designs was analyzed by aligning the dimer of the chimeric protein obtained by AlphaFold2 with the dimer of the protein obtained by SWISS-MODEL and with that of the native protein by means of the PyMOL software. The overlapping dimers were quantified using the RMSD value, where a low value between 0 and 5 represents a good alignment standard while the closer the value is to zero, the higher the similarity between the overlapping protein structures [59]. The overlap of the three dimers was almost perfect with an RMSD value of 0.471, indicating excellent alignment between the three dimers and high similitude between them (Supplementary Figure S4). Therefore, the mutations to design the present chimeric protein did not induce any breakage or relevant affectations in the three-dimensional structure of the enzyme since they presented a correct and expected folding regardless of the design route.

The quality of the model produced by the SWISS-MODEL structure prediction server was further evaluated by the ProSa-Web servers and by the SAVES-6.0 server. The z-score was calculated by ProSa-Web and the ERRAT and Verify3D factors were calculated using the SAVES-6.0 server. The results obtained in ProSa-Web were contrasted with the values corresponding to the structure obtained by the AlphaFold2 server and with the native asparaginase 3ECA derived from *E. coli* to verify that the parameters were similar and contained in a very close range of values.

Using the ProSa-Web server, the z-score value of the chimeric protein obtained in SWISS-MODEL was plotted together with all the z-scores of the proteins. To date, the structure has been experimentally determined by X-ray crystallography and NMR spectroscopy. The z-score obtained for the protein was −11.22 (Supplementary Figure S5). As reported in the literature, proteins with a composition ranging from 300 to 500 amino acid residues have z-scores in the range of −1 to −13 [60]. Therefore, the z-score obtained for this model is within the range of the scores reported for native proteins of similar size since the chimeric protein has 326 residues. This demonstrates the good quality of the 3D structure model generated by the SWISS-MODEL server. The z-score was also calculated for the native 3ECA protein derived from *E. coli* and for the one modeled using AlphaFold2 and reported z-scores of −12.1 and −11.47, respectively (Supplementary Figure S5). These results indicate that the model obtained by SWISS-MODEL presents a quality close to that of the native 3ECA enzyme and the naturally folded chimera by AlphaFold2.

On the other hand, in the ERRAT analysis, a value of 95.94% was obtained. This makes the model obtained by SWISS-MODEL a good one since a reported value of this parameter of 95% or higher is a marker of a good high-resolution model [61]. The quality value obtained in Verify3D was 83.97%, indicating that 83.97% of the residuals have a mean 3D-1D score ≥0.1. This parameter considers quality models to be those whose values exceed 80% [62], so it was again found that the predictive model of the chimeric protein developed in SWISS-MODEL is a quality model that meets the standard of the Verify3D parameter.

This protein folding factor is paramount to the development of chimeras since the basis of this process is a mutation or amino acid substitution. The amino acid sequence is the primary structure of a protein and governs protein folding. Introducing, substituting, or mutating an amino acid can involve steric hindrance, the establishment of nonspecific bonds, and the breaking of bonds that stabilize the protein [56]. This can induce twisting of polypeptide chains, change of the secondary structure of the protein, chain breakage, exposure of residues from the internal environment to the external environment and vice versa, destabilization of the tertiary and quaternary structure of a protein, and even inactivation of the native structure and denaturation of the protein [56]. In addition, it is known that the activities of enzymes are determined by their three-dimensional structure, which in turn is determined by the amino acid sequence. Therefore, the conformational similarity between the humanized chimeric and native 3ECA proteins in their active conformations found in the present study constitutes a relevant factor since it shows that there is a high probability that the chimeric protein, by conserving the folding of the native mold, also preserves the active center and the enzymatic activity. In general, the 3D structure from the chimeric protein designed by homologation modeling in SWISS-MODEL is a good candidate to carry out subsequent studies such as docking and molecular dynamics.

### 2.3. Docking and In Silico Determination of Substrate Affinity by the Chimeric Enzyme

It is possible that the change or modification induced in the original enzyme produces substrate displacement of the active site or keeps it in a closed conformation. In such a case, the chimeric enzyme would be inactive and lose its purpose. This is why verifying the enzymatic activity of the chimeric mutant obtained once it has been modeled is vital to ensuring the efficiency of the mutations made.

In the chimeric protein of the present work designed by SWISS-MODEL, molecular docking was used to verify the presence of enzymatic activity and the conservation of the active site in comparison with the native enzyme.

The docking assay for the chimeric humanized enzyme that was developed showed nine scoring functions that were increasing in value, indicating that the substrate, in this case asparagine, has nine possible binding sites on the enzyme. Within these values, the lowest were −5.8 and −5.6, which represent the positions corresponding to those data where the substrate has greater affinity for the enzyme and the enzyme–substrate complex is more stable. In this case, the value of −5.6 was chosen because these data coincide with the minimum value obtained by the same analysis for the native bacterial enzyme 3ECA. The value corresponding to −5.8 presupposes a hypothetical model where the affinity of the asparaginase–asparagine complex is maximum but transient; it was subsequently found in the absence of a three-dimensional model that justifies this interaction.

**Figure 5:**
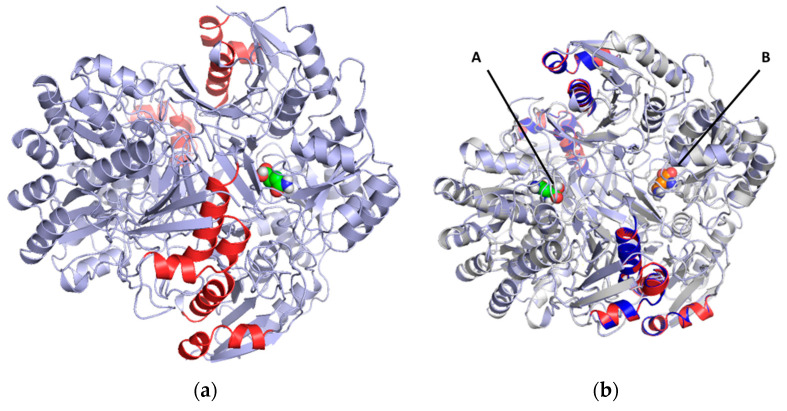

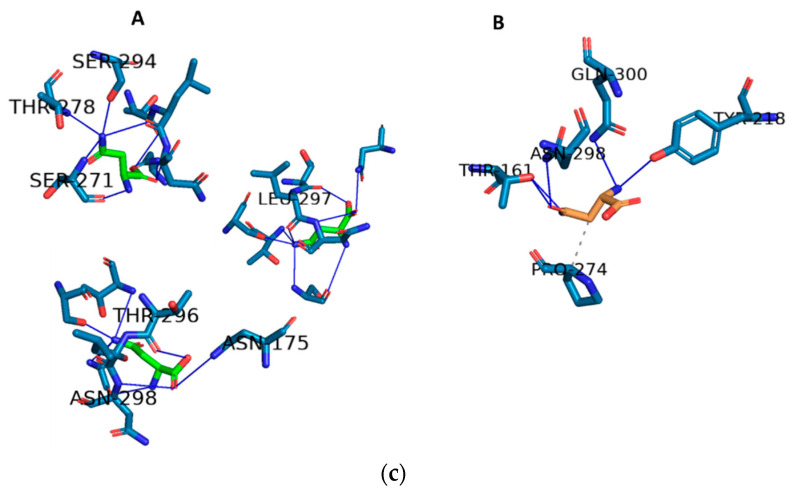
Docking of chimeric humanized L-asparaginase derived from 3ECA with fragments of human L-asparaginase 4O0H with the substrate asparagine. (**a**) The chimeric enzyme is shown in tetrameric conformation with four independently folded subunits. Towards the interior, the asparagine substrate is spherical and is interacting with the enzyme residues (**b**) Alignment of the docking of humanized chimeric L-asparaginase derived from 3ECA with fragments of human L-asparaginase 4O0H with the substrate asparagine and of the docking of the native *E. coli* 3ECA protein with the substrate asparagine. The chimeric enzyme is shown in tetrameric conformation with four independently folded subunits aligned with the L-asparaginase enzyme. Towards the interior, the substrate asparagine has a spherical shape and is interacting with the enzyme residues. (A) corresponds to the interaction with the chimera enzyme and (B) to the interaction with the native *E. coli* template enzyme. There is a 100% structural match with no doublets or chain breaks. Both substrates are arranged in different subunits of the protein. (**c**) Interaction of residues of the active center of chimeric humanized L-asparaginase derived from 3ECA with fragments of human L-asparaginase 4O0H and of the native *E. coli* 3ECA protein with the substrate asparagine. (A) The interaction of the amino acids of the active center of the chimeric enzyme with asparagine is shown. The hydrogen bridge bonds established between the substrate and the amino acids of the active center can be seen in blue. (B) The interaction of the amino acids of the active center of the native *E. coli* enzyme with asparagine is shown. The hydrogen-bridging bonds between the substrate and the amino acids of the active center can be seen in blue and the hydrophobic interactions are represented by a dashed line.

To contrast the adequate docking selection, the three-dimensional structure of the humanized chimeric protein coupled to the substrate was modeled and aligned with its counterpart (the native enzyme 3ECA–substrate complex) (Figure 4B and Figure 5a). The result showed a 100% coincidence in the structural conformation of the two proteins, but instead the substrates were arranged in different dimers almost symmetrically. Previous studies of the structure of the native 3ECA protein showed the existence of four active sites located between the subunits of the intimate dimers. The four active sites are distributed such that there are two in each dimer, making only the tetrameric enzyme functional [55,57]. This increased the probability that the chimeric enzyme was functional, albeit in an active site different from the one modeled for the native 3ECA enzyme.

Each intimate dimer has two-fold symmetry and thus comprises two active sites. Mutational and kinetic studies identified Thr12, Tyr25, Thr89, Asp90, and Lys162 (3ECA numbering) as the residues involved in both the catalytic action and substrate binding [49,62,63] while Ser58, Gln59, and Asn248 were found to be chiefly responsible for substrate binding [62,63]. In the designed chimeric enzyme, these residues were not substituted and therefore are present as part of the catalytic pocket and the loop structure that mediates the passage from the substrate to the active center and catalysis. If function as part of the catalytic action mechanism of the enzyme is intact as well as its proximity to the substrate once positioned within the active center, then this suggests that the catalytic action mechanism of the enzyme is intact. This points to the substrate being located in one of the active centers of the dimer.

To demonstrate the presence of the substrate and an active center, for the three alternative active centers which differ from those of the chimeric protein, the residues that interact and form the active site of both enzymes were determined. The modeling of these residues showed that the developed chimera establishes seven bonds with asparagine, all by hydrogen bonds with the following amino acids: ASN 298, SER 294, ASN 175, THR 296, LEU 297, SER 271, and THR 278. By contrast, the native bacterial mold protein only establishes six bonds: five by hydrogen bonds with the amino acids THR 161, TYR 218, GLU 300, and ASN 298 and one by hydrophobic interaction with PRO 274 (Figure 5c).

**Figure 6:**
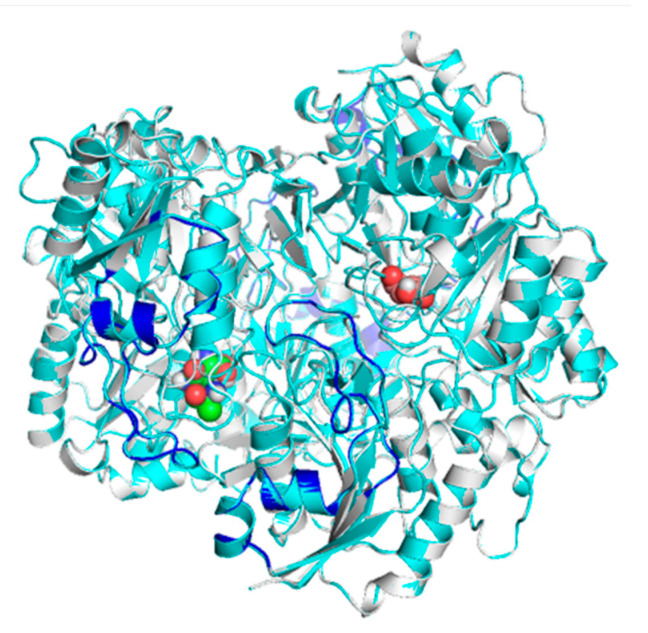
Alignment of the N-terminal end of chimeric humanized *E. coli* L-asparaginase with fragments of human L-asparaginase 4O0H with the N-terminal of the native *E. coli* protein. The N-terminal end that forms the loop that blocks the active center regulating substrate entry and product exit is shown in blue. There is a 100% structural match with no bends or chain breaks.

In terms of residues, it was observed that, in contrast to the native enzyme, the chimera enzyme retained the substrate bond with asparagine 298. The remaining seven bonds are approximately five residues away from their closest corresponding amino acids in the native structure. This is consistent with the active site being considered highly versatile and dynamic [55,57]. Considering that the forces maintaining the structure of the active site are fundamentally weak interactions, different interconvertible conformational states of the active site can exist in a set of molecules of the same enzyme, ranging from those that greatly facilitate binding to the substrate to those that practically do not allow the entry of the substrate, passing through all imaginable intermediate states [55]. Additionally, it is known that the enzyme has a loop towards the N-terminal end that specifically blocks the active site regulating the entry and exit of substrates [55]. Although a small fraction of this loop is modified in the chimeric enzyme as part of the enzyme construction process, it can be seen from an alignment of the chimeric and native sequences and a comparison of this with other homologous proteins that the essential amino acids for the active site are conserved in the chimeric enzyme created. A superposition of the chimeric and template enzymes coupled to the substrate showed the conservation of the structure and position of this loop in the four monomers of both enzymes and their proximity to the substrate (Figure 6).

Taking all these docking data as background, it may be stated that the constructed chimeric enzyme retains its hydrolytic activity and is therefore a functional protein despite the differential substrate arrangement.

## 3. Materials and Methods

### 3.1. Sequence Data of L-asparaginases

For this study, the amino acid sequences as well as the native three-dimensional structure of the L-asparaginases from Escherichia coli type II (EcA) and Homo sapiens were downloaded from the Protein Data Bank (https://www.rcsb.org/ (accessed on 14 March 2022).) with the identification codes 3ECA [55] and 4O0H [63], respectively. For subsequent analyses, the monomers of these enzymes were used.

### 3.2. T-Cell Epitope Prediction and Epitope Density Determination

The NetMHCII 4.0 server (https://services.healthtech.dtu.dk/service.php?NetMHCIIpan-4.0 (accessed on 14 March 2022).) [42] was used for T-cell epitope prediction, setting the default parameters of the program. The program was supplied with the amino acid sequence to be evaluated in pdb and the sequence was fragmented into peptides with lengths ranging from 9 to 15 residues. The alleles to be evaluated were selected and the analysis was started. From the expected data, peptides predicted as “strong binding” (SB ≤ 1%) and “weak binding” (WB ≤ 5%) were selected as immunogenic epitopes. For the calculation of epitope density, the relative frequency was used: fi = ni/N, where ni is the number of predicted immunogenic epitopes and N is the total number of epitopes determined by the program (immunogenic and nonimmunogenic). The epitope density of each protein was determined for the HLA-DRB1*01:01, HLA-DRB1*03:01, HLA-DRB1*04:01, HLA-DRB1*07:01, HLADRB1*08:01, HLA-DRB1*11:01, HLA-DRB1*13:01, and HLADRB1*15:01 alleles, which are reference alleles in the literature with a wide global frequency [43,44,64,65].

### 3.3. Prediction of Allergenic Epitopes

Allergenicity prediction was performed using the AllerTOP v. 2.0 server (http://www.ddg-pharmfac.net/AllerTOP/ (accessed on 16 March 2022).) [66]. Each T-cell epitope predicted as immunogenic for the HLA-DRB1*04:01 and HLA-DRB1*07:01 alleles which fulfilled the conditions indicated above was processed with this tool. The program evaluates the peptide sequence and returns the results as “probable allergen” or “probable non-allergen”.

### 3.4. Epitope Alignment and Mapping

Once the results described above were obtained, the most exposed epitopes were selected and the epitopes of the bacterial enzyme were aligned in the Clustal Omega Program (https://www.ebi.ac.uk/Tools/msa/clustalo/ (accessed on 20 March 2022).) with the human enzyme in search of possible substituents [67]. Next, the PyMOL software (The PyMOL Molecular Graphics System, Version 2.3.1 Schrödinger, LLC) was used to visualize the epitopes in the native structures of the enzymes. Once visualized, the three-dimensional structure of the epitopes was analyzed and the *E. coli* L-asparaginase was humanized with the corresponding substituents.

### 3.5. Modeling of the Three-Dimensional Structure of the Chimeric Enzyme

For the three-dimensional modeling of the protein, the SWISS-MODEL software (https://swissmodel.expasy.org/ (accessed on 15 May 2022).) was used to obtain the three-dimensional structure of the chimeric protein by homology [68]. Subsequently, the quality of the model was evaluated with the Rachamandram plot analysis using the MolProbity tool [69] available on the SWISS-MODEL platform [68].

#### Verify3D Model

To check the homology modeling results performed in SWISS-MODEL, the protein was recreated again using AlphaFold2 AF2 (V2.0.1). This server was used for structure prediction with the necessary databases downloaded from the AF23 GitHub repository [70]. Subsequently, the structures of both designed proteins were compared jointly with that of the 3ECA native by similarity analysis. To assess the similarity of the models, the PDB of each was loaded and superimposed. The overlapping complexes were quantified using the root mean square deviation (RMSD) value [59]. Model visualization and overlay were performed with PyMOL 2.4.0 Molecular Graphics System software (Schrodinger, Inc.) (https://pymol.org/2/ (accessed on 22 February 2023).).

As a counterpart to the verification of the Ramachandran parameters, the quality of each protein model was analyzed with Protein Structure Analysis (ProSA-web) (https://prosa.services.came.sbg.ac.at/prosa.php (accessed on 5 March 2023).) [60] and the model developed in SWISS-MODEL was also evaluated with the SAVES v6.0 (https://saves.mbi.ucla.edu/ (accessed on 5 March 2023).) server [71]. The SAVES v6.0 server includes ERRAT and Verify3D.

### 3.6. In Silico Determination of Substrate Affinity

To determine the substrate affinity in the chimeric enzyme, protein–ligand docking was performed with the Autodock Vina program [72] which is integrated into the PyRx software [73]. To perform the molecular docking, an interaction region was established around the entire heterotetramer generated (blind docking) using the following parameters: center_x = 54.7089; center_y = 20.7493; center_z = 27.1783; size _x = 83.0180121136; size _y = 72.2542178059; size _z = 75.5301778221; and exhaustiveness = 8.0. After checking the interactions of the active center, the Protein–Ligand Interaction Profiler program (https://plip-tool.biotec.tu-dresden.de/plip-web/plip/index (accessed on 16 May 2022).) was used [74]. The docking was validated by retrospective docking [75] (Supplementary Tables S4 and S5).

### 3.7. Statistical Analysis of Epitope Density

To compare the epitope density of both enzymes, a paired *t*-test (*p* < 0.05) was performed with the mean epitope densities using the statistical package GraphPad Prism version 5.0 for Windows (GraphPad Software, San Diego, CA, USA).

## 4. Conclusions

This work focused on the in-silico expression of a chimeric enzyme generated by substituting the antigenic determinants present in the ASNase of *E. coli* 3ECA for regions present in a human enzyme which will make it possible to minimize the recognition of the *E. coli* epitopes by the immune system. The human 4O0H enzyme was used since this enzyme variant has not been used in previous studies. The results obtained demonstrated that it is possible to design a humanized chimeric enzyme with fragments of the human variant of the enzyme using 3ECA as a template. It was found that the human enzyme is a good substitute because it presents less immunogenicity than the bacterial enzyme, although the difference is not significant. The present research allowed the development of a stable chimeric enzyme similar to the commercially validated native variant for the treatment of acute lymphoblastic leukemia, according to the bioinformatics parameters measured. In addition to presenting structural similarities, this modeled enzyme could result in less immunogenicity than the native one, which will be experimentally verified in subsequent studies, as well as the conservation of the catalytic activity by this chimera. The humanized chimera obtained constitutes a step forward in the synthesis of this less immunogenic biopharmaceutical. Although the results obtained are promising, additional studies are required to express this variant in recombinant form, perform stability and enzymatic activity assays in vitro, and test the immunogenicity of the resulting enzyme in immunological assays in vitro and with animal models.

## Data Availability

All data is available as part of Supplementary Materials.

## Supplementary Materials

The following supporting information can be downloaded at: https://www.mdpi.com/article/10.3390/ijms24087550/s1.

## Abbreviations

ALL—acute lymphoblastic leukemia, DNA—deoxyribonucleic acid, EcA—*Escherichia coli* asparaginase, ErA—*Erwinia chrysanthemi* asparaginase, PEG—polyethylene glycol, Asn—asparagine, MHCII—major histocompatibility complex II, ASNase—asparaginase.
