# Supplementary material for: In Silico Design of a Chimeric Humanized L-asparaginase
Source: Int J Mol Sci. 2023 Apr 20;24(8):7550. doi: 10.3390/ijms24087550 (PMC10144303; doi:10.3390/ijms24087550)
Supplement: Supplementary file 1 [file ijms-24-07550-s001.zip › ijms-2206162-SI.pdf]

HMNPIVVVHGGGAGPISKDRKERVHQGMVRAATVGYGILREGGSAV
DAVEGAVVALEDDPEFNAGCGSVLNTNGEVEMDASIMDGKDLSAGA
VSAVQCIANPIKLARLVMEKTPHCFLTDQGAAQFAAAMGVPEIPGEKL
VTERNKKRLEKEKHEKGAQKTDCQKNLGTVGAVALDCKGNVAYATSTG
GIVNKMVGRVGDSPCLGAGGYADNDIGAVSTTGHGESILKVNLARLTL
FHIEQGKTVEEAADLSLGYMKSRVKGLGGLIVVSKTGDWVAKWTSTS
MPWAAAK DGKLHFGIDPDDTTITDLP

**Figure S1.** Amino acid sequence of Homo sapiens Asparaginase 4O0H extracted from the Protein Data Bank website.

LPNITILATGGTIAGGGDSATKSNYTVGKVGVENLVNAVPQLKDIANVK
GEQVVNIGSQDMNDNVWLTLAKKINTDCDKTDGFVITHGTDTMEET
AYFLDLTVKCDKPVVMVGAMRPSTSMSADGPFNLYNAVVTAADKASA
NRGVLVVMNDTVLDGRDVTKTNTTDVATFKSVNYGPLGYIHNGKIDY
QRTPARKHTSDTPFDVSKLNELPKVGIVYNYANASDLPAKALVDAGYD
GIVSAGVGNGNLYKSVFDTLATAAKTGTAVVRSSRVPTGATTQDAEVD
DAKYGFV ASGTLNPQKARVLLQLALTQTKDPQQIQQIFNQY

**Figure S2.** Amino acid sequence of Escherichia coli Asparaginase 3ECA extracted from the Protein Data Bank website.

LPNITILATGGTIAGGGDSATKSNYTVGKVGVDAVEGAVVALEDDPNV
KGEQVVNIGSQDMNDNVWLTLAKKINTDCDKTDGFVITHGTDTMEE
TAYFLDLTVKCDKPVVMVGAMRPSTSMSAANPIKLARLVMEKTPKAS
ANRGVLVVMNDTVLDGRDVTKTNTTDVATFKSVNYGPLGYIHNGKID
YQRTPARKHTSDTPFDVSKLNELPKVGIVYNYANASDLPAKALVDAGYD
GIVSAGVGNGNLYKSVFDTLATAAKTGTAVVRSSRVPTGATTQDAEVD
DAKYGFV ASGTLNPQKARVLLQLALTQTKDPQQIQQIFNQY

**Figure S3.** Amino acid sequence of Humanized Chimeric Asparaginase engineered with human asparaginase fragments. The residues corresponding to human asparaginase that were inserted as substituents in the E.coli enzyme are highlighted in blue.

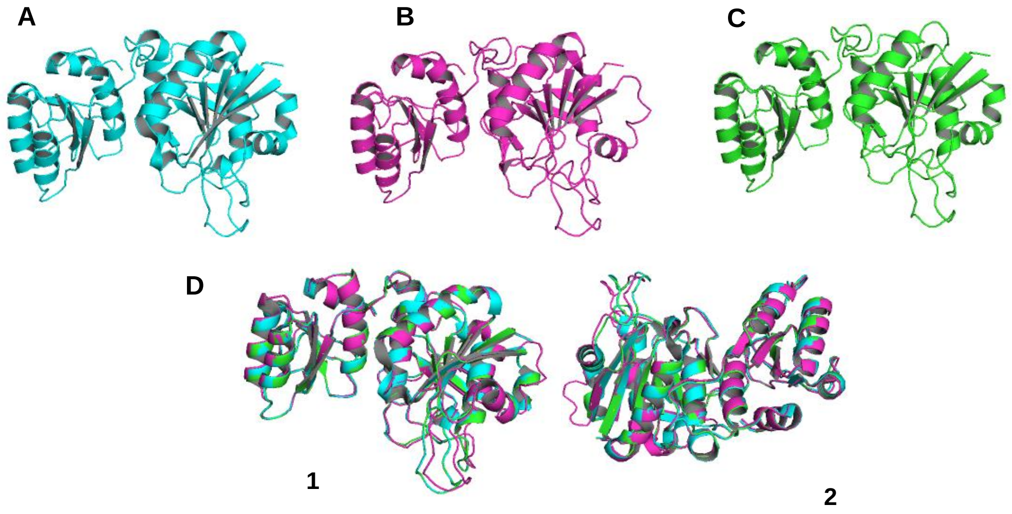


**Figure S4. Validation of the three-dimensional structure of the humanized 3ECA chimeric enzyme predicted by the SWISS Model server by similarity analysis of its AC dimers with the BD dimers of the native 3ECA protein and the chimeric protein model predicted by AlphaFold 2. A.** Structure of the BD dimer of the native 3ECA protein**. B.** BD dimer structure of the humanized 3ECA chimeric protein obtained from the AlphaFold2 server. **C.** Structure of the BD dimer of the humanized 3ECA chimeric protein obtained from the SWISS Model server. **D.** Alienation of the 3 structures with a view from two different psotions. The root mean square deviation (RMSD) was used to calculate the quality of aliasing and reported an RMSD value of 0.47 . All visualized structures were performed with PyMOL 2.4.0 Molecular Graphics System software as well as the alignment.

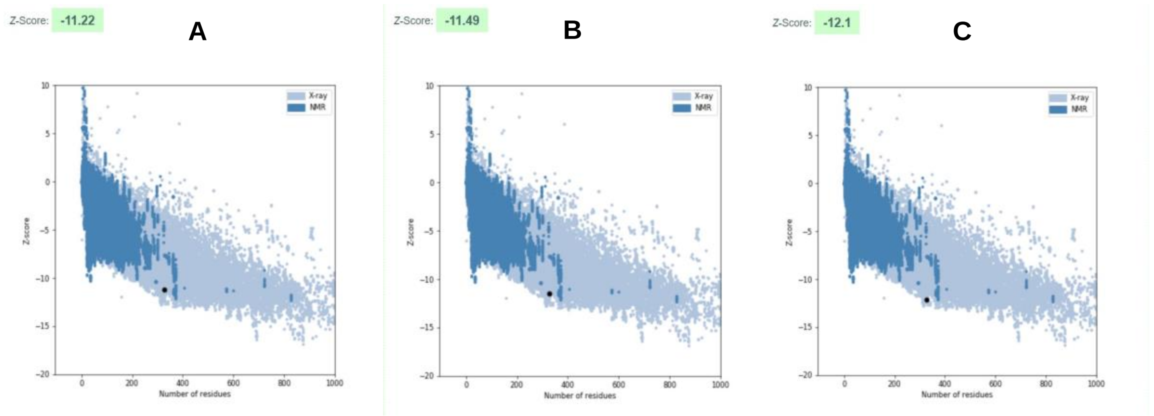


**Figure S5. Graph of Protein Data Bank (PDB) protein structures (blue), (A) 3ECA chimeric enzyme predicted by the SWISS Model server (black), (B ) the chimeric protein model predicted by AlphaFold 2 and (C) native 3ECA protein (black) , all with z-scores computed by ProSA-web server.** PDB structures determined by X-ray crystallography and nuclear magnetic resonance (NMR) were shown in light blue and dark blue, respectiely. Models generated by SWISS-MODEL , Alpha Fold and 3ECA Native from PDB server were shown in circle.

Table S1. Output of the NetMHCIIpan version 4.0 program. for the 3ECA protein. Of a total of 2025 fragmented peptides, only those that bind to MHCII either weakly or strongly and are therefore considered antigenic are presented. In green The antigenic peptides that additionally present allergenicity, determined by the AllerTOP server, are represented for the DRB1_0401 and DRB1_0701 allele. Additionally in blue the peptides that were selected as surrogates for designing the humanized chimeric asparaginase are indicated.

# NetMHCIIpan version 4.0

# Input is in FASTA format

# Peptide length 9,10,11,12,13,14,15

# Prediction Mode: EL

# Threshold for Strong binding peptides (%Rank)1%
# Threshold for Weak binding peptides (%Rank) 5%

# Allele: DRB1_0101

| Pos | MHC | Peptide | Of | Core | Core_Rel | Identity | Score_EL | %Rank_EL | Exp_Bind | BindLevel |
|---|---|---|---|---|---|---|---|---|---|---|
| 215 | DRB1_0101 | GIVYNYANASDLPA | 3 | YNYANASDL | 0.987 | 3ECA_A_PDBID_CH | 0.856610 | 0.60 | NA | <=SB |
| 216 | DRB1_0101 | IVYNYANASDLPAK | 2 | YNYANASDL | 0.987 | 3ECA_A_PDBID_CH | 0.805187 | 0.85 | NA | <=SB |
| 233 | DRB1_0101 | DAGYDGIVSAGVGN | 3 | YDGIVSAGV | 1.000 | 3ECA_A_PDBID_CH | 0.776991 | 0.98 | NA | <=SB |
| 214 | DRB1_0101 | VGIVYNYANASDLPA | 4 | YNYANASDL | 0.967 | 3ECA_A_PDBID_CH | 0.842813 | 0.67 | NA | <=SB |
| 215 | DRB1_0101 | GIVYNYANASDLPAK | 3 | YNYANASDL | 1.000 | 3ECA_A_PDBID_CH | 0.939400 | 0.26 | NA | <=SB |
| 232 | DRB1_0101 | VDAGYDGIVSAGVGN | 4 | YDGIVSAGV | 1.000 | 3ECA_A_PDBID_CH | 0.787291 | 0.94 | NA | <=SB |
| 233 | DRB1_0101 | DAGYDGIVSAGVGNG | 3 | YDGIVSAGV | 1.000 | 3ECA_A_PDBID_CH | 0.889292 | 0.47 | NA | <=SB |
| 286 | DRB1_0101 | DAKYGFVASGTLNPQ | 3 | YGFVASGTL | 1.000 | 3ECA_A_PDBID_CH | 0.789841 | 0.92 | NA | <=SB |
| 42 | DRB1_0101 | LKDIANVKGEQVV | 3 | IANVKGEQV | 1.000 | 3ECA_A_PDBID_CH | 0.453290 | 2.93 | NA | <=WB |
| 43 | DRB1_0101 | KDIANVKGEQVVN | 2 | IANVKGEQV | 1.000 | 3ECA_A_PDBID_CH | 0.587101 | 1.95 | NA | <=WB |
| 80 | DRB1_0101 | TDGFVITHGTDTM | 3 | FVITHGTDT | 1.000 | 3ECA_A_PDBID_CH | 0.395657 | 3.45 | NA | <=WB |
| 81 | DRB1_0101 | DGFVITHGTDTME | 2 | FVITHGTDT | 1.000 | 3ECA_A_PDBID_CH | 0.365173 | 3.76 | NA | <=WB |
| 213 | DRB1_0101 | KVGIVYNYANASD | 3 | IVYNYANAS | 1.000 | 3ECA_A_PDBID_CH | 0.431889 | 3.12 | NA | <=WB |
| 215 | DRB1_0101 | GIVYNYANASDLP | 3 | YNYANASDL | 0.967 | 3ECA_A_PDBID_CH | 0.692847 | 1.39 | NA | <=WB |

| | | | | | | | | | | |
|---|---|---|---|---|---|---|---|---|---|---|
| 216 | DRB1_0101 | IVYNYANASDLPA | 2 | YNYANASDL | 1.000 | 3ECA_A_PDBID_CH | 0.626087 | 1.73 | NA | <=WB |
| 217 | DRB1_0101 | VYNYANASDLPAK | 1 | YNYANASDL | 0.833 | 3ECA_A_PDBID_CH | 0.423387 | 3.19 | NA | <=WB |
| 233 | DRB1_0101 | DAGYDGIVSAGVG | 3 | YDGIVSAGV | 1.000 | 3ECA_A_PDBID_CH | 0.566505 | 2.08 | NA | <=WB |
| 234 | DRB1_0101 | AGYDGIVSAGVGN | 2 | YDGIVSAGV | 1.000 | 3ECA_A_PDBID_CH | 0.519855 | 2.39 | NA | <=WB |
| 286 | DRB1_0101 | DAKYGFVASGTLN | 3 | YGFVASGTL | 0.993 | 3ECA_A_PDBID_CH | 0.412389 | 3.29 | NA | <=WB |
| 287 | DRB1_0101 | AKYGFVASGTLNP | 2 | YGFVASGTL | 0.987 | 3ECA_A_PDBID_CH | 0.385619 | 3.54 | NA | <=WB |
| 294 | DRB1_0101 | SGTLNPQKARVLL | 3 | LNPQKARVL | 1.000 | 3ECA_A_PDBID_CH | 0.293319 | 4.59 | NA | <=WB |
| 295 | DRB1_0101 | GTLNPQKARVLLQ | 2 | LNPQKARVL | 1.000 | 3ECA_A_PDBID_CH | 0.269743 | 4.94 | NA | <=WB |
| 3 | DRB1_0101 | NITILATGGTIAGG | 3 | ILATGGTIA | 0.993 | 3ECA_A_PDBID_CH | 0.318591 | 4.28 | NA | <=WB |
| 41 | DRB1_0101 | QLKDIANVKGEQVV | 4 | IANVKGEQV | 1.000 | 3ECA_A_PDBID_CH | 0.413779 | 3.28 | NA | <=WB |
| 42 | DRB1_0101 | LKDIANVKGEQVVN | 3 | IANVKGEQV | 1.000 | 3ECA_A_PDBID_CH | 0.683266 | 1.44 | NA | <=WB |
| 43 | DRB1_0101 | KDIANVKGEQVVNI | 2 | IANVKGEQV | 1.000 | 3ECA_A_PDBID_CH | 0.582497 | 1.98 | NA | <=WB |
| 79 | DRB1_0101 | KTDGFVITHGTDTM | 4 | FVITHGTDT | 1.000 | 3ECA_A_PDBID_CH | 0.353500 | 3.88 | NA | <=WB |
| 80 | DRB1_0101 | TDGFVITHGTDTME | 3 | FVITHGTDT | 1.000 | 3ECA_A_PDBID_CH | 0.570299 | 2.06 | NA | <=WB |
| 81 | DRB1_0101 | DGFVITHGTDTMEE | 2 | FVITHGTDT | 1.000 | 3ECA_A_PDBID_CH | 0.444882 | 3.00 | NA | <=WB |
| 94 | DRB1_0101 | ETAYFLDLTVKCDK | 3 | YFLDLTVKC | 0.980 | 3ECA_A_PDBID_CH | 0.288631 | 4.66 | NA | <=WB |
| 144 | DRB1_0101 | RGVLVVMNDTVLDG | 3 | LVVMNDTVL | 0.993 | 3ECA_A_PDBID_CH | 0.382118 | 3.58 | NA | <=WB |
| 145 | DRB1_0101 | GVLVVMNDTVLDGR | 2 | LVVMNDTVL | 0.980 | 3ECA_A_PDBID_CH | 0.276061 | 4.85 | NA | <=WB |
| 212 | DRB1_0101 | PKVGIVYNYANASD | 4 | IVYNYANAS | 1.000 | 3ECA_A_PDBID_CH | 0.392505 | 3.48 | NA | <=WB |
| 213 | DRB1_0101 | KVGIVYNYANASDL | 3 | IVYNYANAS | 0.907 | 3ECA_A_PDBID_CH | 0.445260 | 3.00 | NA | <=WB |
| 214 | DRB1_0101 | VGIVYNYANASDLP | 4 | YNYANASDL | 0.833 | 3ECA_A_PDBID_CH | 0.675097 | 1.48 | NA | <=WB |
| 217 | DRB1_0101 | VYNYANASDLPAKA | 1 | YNYANASDL | 0.533 | 3ECA_A_PDBID_CH | 0.389067 | 3.51 | NA | <=WB |
| 232 | DRB1_0101 | VDAGYDGIVSAGVG | 4 | YDGIVSAGV | 1.000 | 3ECA_A_PDBID_CH | 0.518277 | 2.40 | NA | <=WB |
| 234 | DRB1_0101 | AGYDGIVSAGVGNG | 2 | YDGIVSAGV | 1.000 | 3ECA_A_PDBID_CH | 0.648984 | 1.61 | NA | <=WB |
| 285 | DRB1_0101 | DDAKYGFVASGTLN | 4 | YGFVASGTL | 0.987 | 3ECA_A_PDBID_CH | 0.348675 | 3.93 | NA | <=WB |
| 286 | DRB1_0101 | DAKYGFVASGTLNP | 3 | YGFVASGTL | 0.993 | 3ECA_A_PDBID_CH | 0.663099 | 1.54 | NA | <=WB |

| | | | | | | | | | | |
|---|---|---|---|---|---|---|---|---|---|---|
| 287 | DRB1_0101 | AKYGFVASGTLNPQ | 2 | YGFVASGTL | 0.980 | 3ECA_A_PDBID_CH | 0.486700 | 2.64 | NA | <=WB |
| 294 | DRB1_0101 | SGTLNPQKARVLLQ | 3 | LNPQKARVL | 1.000 | 3ECA_A_PDBID_CH | 0.455215 | 2.91 | NA | <=WB |
| 2 | DRB1_0101 | PNITILATGGTIAGG | 4 | ILATGGTIA | 0.853 | 3ECA_A_PDBID_CH | 0.331687 | 4.12 | NA | <=WB |
| 3 | DRB1_0101 | NITILATGGTIAGGG | 3 | ILATGGTIA | 1.000 | 3ECA_A_PDBID_CH | 0.493740 | 2.58 | NA | <=WB |
| 40 | DRB1_0101 | PQLKDIANVKGEQVV | 5 | IANVKGEQV | 0.967 | 3ECA_A_PDBID_CH | 0.342488 | 3.99 | NA | <=WB |
| 41 | DRB1_0101 | QLKDIANVKGEQVVN | 4 | IANVKGEQV | 1.000 | 3ECA_A_PDBID_CH | 0.680513 | 1.45 | NA | <=WB |
| 42 | DRB1_0101 | LKDIANVKGEQVVNI | 3 | IANVKGEQV | 1.000 | 3ECA_A_PDBID_CH | 0.692057 | 1.39 | NA | <=WB |
| 43 | DRB1_0101 | KDIANVKGEQVVNIG | 2 | IANVKGEQV | 0.987 | 3ECA_A_PDBID_CH | 0.475962 | 2.73 | NA | <=WB |
| 62 | DRB1_0101 | NDNVWLTLAKKINTD | 3 | VWLTLAKKI | 0.953 | 3ECA_A_PDBID_CH | 0.266068 | 5.00 | NA | <=WB |
| 78 | DRB1_0101 | DKTDGFVITHGTDTM | 5 | FVITHGTDT | 1.000 | 3ECA_A_PDBID_CH | 0.300074 | 4.49 | NA | <=WB |
| 79 | DRB1_0101 | KTDGFVITHGTDTME | 4 | FVITHGTDT | 1.000 | 3ECA_A_PDBID_CH | 0.570440 | 2.06 | NA | <=WB |
| 80 | DRB1_0101 | TDGFVITHGTDTMEE | 3 | FVITHGTDT | 1.000 | 3ECA_A_PDBID_CH | 0.690766 | 1.40 | NA | <=WB |
| 81 | DRB1_0101 | DGFVITHGTDTMEET | 2 | FVITHGTDT | 1.000 | 3ECA_A_PDBID_CH | 0.362081 | 3.79 | NA | <=WB |
| 93 | DRB1_0101 | EETAYFLDLTVKCDK | 4 | YFLDLTVKC | 0.993 | 3ECA_A_PDBID_CH | 0.272270 | 4.90 | NA | <=WB |
| 94 | DRB1_0101 | ETAYFLDLTVKCDKP | 3 | YFLDLTVKC | 0.993 | 3ECA_A_PDBID_CH | 0.415586 | 3.27 | NA | <=WB |
| 124 | DRB1_0101 | DGPFNLYNAVVTAAD | 3 | FNLYNAVVT | 0.987 | 3ECA_A_PDBID_CH | 0.393252 | 3.47 | NA | <=WB |
| 143 | DRB1_0101 | NRGVLVVMNDTVLDG | 4 | LVVMNDTVL | 0.947 | 3ECA_A_PDBID_CH | 0.388702 | 3.51 | NA | <=WB |
| 144 | DRB1_0101 | RGVLVVMNDTVLDGR | 3 | LVVMNDTVL | 1.000 | 3ECA_A_PDBID_CH | 0.571900 | 2.05 | NA | <=WB |
| 211 | DRB1_0101 | LPKVGIVYNYANASD | 5 | IVYNYANAS | 1.000 | 3ECA_A_PDBID_CH | 0.325031 | 4.20 | NA | <=WB |
| 212 | DRB1_0101 | PKVGIVYNYANASDL | 4 | IVYNYANAS | 0.933 | 3ECA_A_PDBID_CH | 0.404097 | 3.37 | NA | <=WB |
| 213 | DRB1_0101 | KVGIVYNYANASDLP | 5 | YNYANASDL | 0.467 | 3ECA_A_PDBID_CH | 0.710908 | 1.30 | NA | <=WB |
| 216 | DRB1_0101 | IVYNYANASDLPAKA | 2 | YNYANASDL | 0.907 | 3ECA_A_PDBID_CH | 0.715427 | 1.27 | NA | <=WB |
| 217 | DRB1_0101 | VYNYANASDLPAKAL | 3 | YANASDLPA | 0.513 | 3ECA_A_PDBID_CH | 0.307190 | 4.41 | NA | <=WB |
| 231 | DRB1_0101 | LVDAGYDGIVSAGVG | 5 | YDGIVSAGV | 1.000 | 3ECA_A_PDBID_CH | 0.477626 | 2.72 | NA | <=WB |
| 234 | DRB1_0101 | AGYDGIVSAGVGNGN | 2 | YDGIVSAGV | 0.987 | 3ECA_A_PDBID_CH | 0.562906 | 2.11 | NA | <=WB |
| 247 | DRB1_0101 | GNLYKSVFDTLATAA | 3 | YKSVFDTLA | 1.000 | 3ECA_A_PDBID_CH | 0.268710 | 4.96 | NA | <=WB |

| | | | | | | | | | | |
|---|---|---|---|---|---|---|---|---|---|---|
| 284 | DRB1_0101 | VDDAKYGFVASGTLN | 5 | YGFVASGTL | 0.993 | 3ECA_A_PDBID_CH | 0.299478 | 4.50 | NA | <=WB |
| 285 | DRB1_0101 | DDAKYGFVASGTLNP | 4 | YGFVASGTL | 0.993 | 3ECA_A_PDBID_CH | 0.653044 | 1.59 | NA | <=WB |
| 287 | DRB1_0101 | AKYGFVASGTLNPQK | 2 | YGFVASGTL | 0.920 | 3ECA_A_PDBID_CH | 0.389288 | 3.50 | NA | <=WB |
| 293 | DRB1_0101 | ASGTLNPQKARVLLQ | 4 | LNPQKARVL | 1.000 | 3ECA_A_PDBID_CH | 0.419235 | 3.23 | NA | <=WB |
| 294 | DRB1_0101 | SGTLNPQKARVLLQL | 3 | LNPQKARVL | 1.000 | 3ECA_A_PDBID_CH | 0.376540 | 3.64 | NA | <=WB |

# Allele: DRB1_0301

| | | | | | | | | | | |
|---|---|---|---|---|---|---|---|---|---|---|
| 100 | DRB1_0301 | DLTVKCDKPVVMV | 3 | VKCDKPVVM | 1.000 | 3ECA_A_PDBID_CH | 0.377936 | 2.99 | NA | <=WB |
| 101 | DRB1_0301 | LTVKCDKPVVMVG | 2 | VKCDKPVVM | 1.000 | 3ECA_A_PDBID_CH | 0.295409 | 3.85 | NA | <=WB |
| 184 | DRB1_0301 | NGKIDYQRTPARK | 3 | IDYQRTPAR | 1.000 | 3ECA_A_PDBID_CH | 0.270114 | 4.21 | NA | <=WB |
| 185 | DRB1_0301 | GKIDYQRTPARKH | 2 | IDYQRTPAR | 1.000 | 3ECA_A_PDBID_CH | 0.226714 | 4.92 | NA | <=WB |
| 213 | DRB1_0301 | KVGIVYNYANASD | 3 | IVYNYANAS | 1.000 | 3ECA_A_PDBID_CH | 0.305954 | 3.72 | NA | <=WB |
| 99 | DRB1_0301 | LDLTVKCDKPVVMV | 4 | VKCDKPVVM | 1.000 | 3ECA_A_PDBID_CH | 0.340310 | 3.34 | NA | <=WB |
| 100 | DRB1_0301 | DLTVKCDKPVVMVG | 3 | VKCDKPVVM | 1.000 | 3ECA_A_PDBID_CH | 0.481993 | 2.17 | NA | <=WB |
| 101 | DRB1_0301 | LTVKCDKPVVMVGA | 2 | VKCDKPVVM | 1.000 | 3ECA_A_PDBID_CH | 0.322670 | 3.50 | NA | <=WB |
| 145 | DRB1_0301 | GVLVVMNDTVLDGR | 3 | VVMNDTVLD | 0.533 | 3ECA_A_PDBID_CH | 0.240197 | 4.69 | NA | <=WB |
| 146 | DRB1_0301 | VLVVMNDTVLDGRD | 3 | VMNDTVLDG | 0.700 | 3ECA_A_PDBID_CH | 0.299411 | 3.80 | NA | <=WB |
| 178 | DRB1_0301 | PLGYIHNGKIDYQR | 3 | YIHNGKIDY | 0.947 | 3ECA_A_PDBID_CH | 0.230814 | 4.85 | NA | <=WB |
| 183 | DRB1_0301 | HNGKIDYQRTPARK | 4 | IDYQRTPAR | 1.000 | 3ECA_A_PDBID_CH | 0.255598 | 4.43 | NA | <=WB |
| 184 | DRB1_0301 | NGKIDYQRTPARKH | 3 | IDYQRTPAR | 1.000 | 3ECA_A_PDBID_CH | 0.327924 | 3.45 | NA | <=WB |
| 185 | DRB1_0301 | GKIDYQRTPARKHT | 2 | IDYQRTPAR | 0.987 | 3ECA_A_PDBID_CH | 0.231616 | 4.84 | NA | <=WB |
| 212 | DRB1_0301 | PKVGIVYNYANASD | 4 | IVYNYANAS | 0.993 | 3ECA_A_PDBID_CH | 0.277959 | 4.09 | NA | <=WB |
| 213 | DRB1_0301 | KVGIVYNYANASDL | 3 | IVYNYANAS | 0.993 | 3ECA_A_PDBID_CH | 0.286539 | 3.97 | NA | <=WB |

| | | | | | | | | | | |
|---|---|---|---|---|---|---|---|---|---|---|
| 94 | DRB1_0301 | ETAYFLDLTVKCDKP | 3 | YFLDLTVKC | 0.993 | 3ECA_A_PDBID_CH | 0.262903 | 4.32 | NA | <=WB |
| 98 | DRB1_0301 | FLDLTVKCDKPVVMV | 5 | VKCDKPVVM | 1.000 | 3ECA_A_PDBID_CH | 0.304364 | 3.74 | NA | <=WB |
| 99 | DRB1_0301 | LDLTVKCDKPVVMVG | 4 | VKCDKPVVM | 1.000 | 3ECA_A_PDBID_CH | 0.475115 | 2.22 | NA | <=WB |
| 100 | DRB1_0301 | DLTVKCDKPVVMVGA | 3 | VKCDKPVVM | 1.000 | 3ECA_A_PDBID_CH | 0.537227 | 1.80 | NA | <=WB |
| 101 | DRB1_0301 | LTVKCDKPVVMVGAM | 2 | VKCDKPVVM | 1.000 | 3ECA_A_PDBID_CH | 0.256839 | 4.41 | NA | <=WB |
| 118 | DRB1_0301 | STSMSADGPFNLYNA | 3 | MSADGPFNL | 1.000 | 3ECA_A_PDBID_CH | 0.267122 | 4.26 | NA | <=WB |
| 144 | DRB1_0301 | RGVLVVMNDTVLDGR | 4 | VVMNDTVLD | 0.507 | 3ECA_A_PDBID_CH | 0.271332 | 4.19 | NA | <=WB |
| 145 | DRB1_0301 | GVLVVMNDTVLDGRD | 4 | VMNDTVLDG | 0.533 | 3ECA_A_PDBID_CH | 0.409175 | 2.73 | NA | <=WB |
| 146 | DRB1_0301 | VLVVMNDTVLDGRDV | 3 | VMNDTVLDG | 0.773 | 3ECA_A_PDBID_CH | 0.331094 | 3.42 | NA | <=WB |
| 150 | DRB1_0301 | MNDTVLDGRDVTKTN | 3 | TVLDGRDVT | 0.993 | 3ECA_A_PDBID_CH | 0.275046 | 4.13 | NA | <=WB |
| 178 | DRB1_0301 | PLGYIHNGKIDYQRT | 3 | YIHNGKIDY | 0.953 | 3ECA_A_PDBID_CH | 0.237083 | 4.74 | NA | <=WB |
| 182 | DRB1_0301 | IHNGKIDYQRTPARK | 5 | IDYQRTPAR | 0.993 | 3ECA_A_PDBID_CH | 0.266253 | 4.27 | NA | <=WB |
| 183 | DRB1_0301 | HNGKIDYQRTPARKH | 4 | IDYQRTPAR | 1.000 | 3ECA_A_PDBID_CH | 0.359918 | 3.16 | NA | <=WB |
| 184 | DRB1_0301 | NGKIDYQRTPARKHT | 3 | IDYQRTPAR | 1.000 | 3ECA_A_PDBID_CH | 0.379228 | 2.98 | NA | <=WB |
| 211 | DRB1_0301 | LPKVGIVYNYANASD | 5 | IVYNYANAS | 0.980 | 3ECA_A_PDBID_CH | 0.256249 | 4.42 | NA | <=WB |
| 212 | DRB1_0301 | PKVGIVYNYANASDL | 4 | IVYNYANAS | 1.000 | 3ECA_A_PDBID_CH | 0.288226 | 3.94 | NA | <=WB |
| 213 | DRB1_0301 | KVGIVYNYANASDLP | 3 | IVYNYANAS | 0.987 | 3ECA_A_PDBID_CH | 0.374633 | 3.02 | NA | <=WB |

# Allele: DRB1_0401

| | | | | | | | | | | |
|---|---|---|---|---|---|---|---|---|---|---|
| 218 | DRB1_0401 | YNYANASDLPAK | 2 | YANASDLPA | 1.000 | 3ECA_A_PDBID_CH | 0.699347 | 0.84 | NA | <=SB |
| 213 | DRB1_0401 | KVGIVYNYANASD | 3 | IVYNYANAS | 1.000 | 3ECA_A_PDBID_CH | 0.736986 | 0.67 | NA | <=SB |
| 217 | DRB1_0401 | VYNYANASDLPAK | 3 | YANASDLPA | 1.000 | 3ECA_A_PDBID_CH | 0.909565 | 0.13 | NA | <=SB |
| 218 | DRB1_0401 | YNYANASDLPAKA | 2 | YANASDLPA | 1.000 | 3ECA_A_PDBID_CH | 0.894517 | 0.16 | NA | <=SB |
| 39 | DRB1_0401 | VPQLKDIANVKGEQ | 3 | LKDIANVKG | 1.000 | 3ECA_A_PDBID_CH | 0.671939 | 0.96 | NA | <=SB |
| 94 | DRB1_0401 | ETAYFLDLTVKCDK | 3 | YFLDLTVKC | 0.993 | 3ECA_A_PDBID_CH | 0.688832 | 0.88 | NA | <=SB |

| | | | | | | | | | | |
|---|---|---|---|---|---|---|---|---|---|---|
| 212 | DRB1_0401 | PKVGIVYNYANASD | 4 | IVYNYANAS | 1.000 | 3ECA_A_PDBID_CH | 0.715918 | 0.76 | NA | <=SB |
| 213 | DRB1_0401 | KVGIVYNYANASDL | 3 | IVYNYANAS | 0.993 | 3ECA_A_PDBID_CH | 0.727693 | 0.70 | NA | <=SB |
| 216 | DRB1_0401 | IVYNYANASDLPAK | 4 | YANASDLPA | 1.000 | 3ECA_A_PDBID_CH | 0.895092 | 0.16 | NA | <=SB |
| 217 | DRB1_0401 | VYNYANASDLPAKA | 3 | YANASDLPA | 1.000 | 3ECA_A_PDBID_CH | 0.945743 | 0.06 | NA | <=SB |
| 218 | DRB1_0401 | YNYANASDLPAKAL | 2 | YANASDLPA | 1.000 | 3ECA_A_PDBID_CH | 0.845737 | 0.30 | NA | <=SB |
| 247 | DRB1_0401 | GNLYKSVFDTLATA | 3 | YKSVFDTLA | 1.000 | 3ECA_A_PDBID_CH | 0.670025 | 0.97 | NA | <=SB |
| 288 | DRB1_0401 | KYGFVASGTLNPQK | 3 | FVASGTLNP | 1.000 | 3ECA_A_PDBID_CH | 0.709457 | 0.79 | NA | <=SB |
| 38 | DRB1_0401 | AVPQLKDIANVKGEQ | 4 | LKDIANVKG | 1.000 | 3ECA_A_PDBID_CH | 0.663240 | 1.00 | NA | <=SB |
| 39 | DRB1_0401 | VPQLKDIANVKGEQV | 3 | LKDIANVKG | 1.000 | 3ECA_A_PDBID_CH | 0.676495 | 0.94 | NA | <=SB |
| 94 | DRB1_0401 | ETAYFLDLTVKCDKP | 3 | YFLDLTVKC | 1.000 | 3ECA_A_PDBID_CH | 0.769601 | 0.55 | NA | <=SB |
| 126 | DRB1_0401 | PFNLYNAVVTAADKA | 4 | YNAVVTAAD | 0.693 | 3ECA_A_PDBID_CH | 0.681508 | 0.92 | NA | <=SB |
| 127 | DRB1_0401 | FNLYNAVVTAADKAS | 3 | YNAVVTAAD | 0.987 | 3ECA_A_PDBID_CH | 0.689372 | 0.88 | NA | <=SB |
| 213 | DRB1_0401 | KVGIVYNYANASDLP | 3 | IVYNYANAS | 0.993 | 3ECA_A_PDBID_CH | 0.758515 | 0.59 | NA | <=SB |
| 215 | DRB1_0401 | GIVYNYANASDLPAK | 5 | YANASDLPA | 0.967 | 3ECA_A_PDBID_CH | 0.859535 | 0.26 | NA | <=SB |
| 216 | DRB1_0401 | IVYNYANASDLPAKA | 4 | YANASDLPA | 1.000 | 3ECA_A_PDBID_CH | 0.936469 | 0.07 | NA | <=SB |
| 217 | DRB1_0401 | VYNYANASDLPAKAL | 3 | YANASDLPA | 1.000 | 3ECA_A_PDBID_CH | 0.916075 | 0.11 | NA | <=SB |
| 218 | DRB1_0401 | YNYANASDLPAKALV | 2 | YANASDLPA | 1.000 | 3ECA_A_PDBID_CH | 0.761364 | 0.58 | NA | <=SB |
| 247 | DRB1_0401 | GNLYKSVFDTLATAA | 3 | YKSVFDTLA | 1.000 | 3ECA_A_PDBID_CH | 0.723465 | 0.72 | NA | <=SB |
| 287 | DRB1_0401 | AKYGFVASGTLNPQK | 4 | FVASGTLNP | 0.993 | 3ECA_A_PDBID_CH | 0.664480 | 1.00 | NA | <=SB |
| 288 | DRB1_0401 | KYGFVASGTLNPQKA | 3 | FVASGTLNP | 1.000 | 3ECA_A_PDBID_CH | 0.725718 | 0.71 | NA | <=SB |
| 128 | DRB1_0401 | NLYNAVVTAADK | 2 | YNAVVTAAD | 0.960 | 3ECA_A_PDBID_CH | 0.345675 | 3.77 | NA | <=WB |
| 214 | DRB1_0401 | VGIVYNYANASD | 2 | IVYNYANAS | 1.000 | 3ECA_A_PDBID_CH | 0.382356 | 3.26 | NA | <=WB |
| 217 | DRB1_0401 | VYNYANASDLPA | 3 | YANASDLPA | 0.973 | 3ECA_A_PDBID_CH | 0.323084 | 4.13 | NA | <=WB |
| 219 | DRB1_0401 | NYANASDLPAKA | 1 | YANASDLPA | 1.000 | 3ECA_A_PDBID_CH | 0.479246 | 2.28 | NA | <=WB |
| 39 | DRB1_0401 | VPQLKDIANVKGE | 3 | LKDIANVKG | 1.000 | 3ECA_A_PDBID_CH | 0.550328 | 1.72 | NA | <=WB |
| 40 | DRB1_0401 | PQLKDIANVKGEQ | 2 | LKDIANVKG | 1.000 | 3ECA_A_PDBID_CH | 0.531248 | 1.86 | NA | <=WB |

| | | | | | | | | | | |
|---|---|---|---|---|---|---|---|---|---|---|
| 94 | DRB1_0401 | ETAYFLDLTVKCD | 3 | YFLDLTVKC | 1.000 | 3ECA_A_PDBID_CH | 0.537989 | 1.81 | NA | <=WB |
| 95 | DRB1_0401 | TAYFLDLTVKCDK | 2 | YFLDLTVKC | 0.980 | 3ECA_A_PDBID_CH | 0.528021 | 1.89 | NA | <=WB |
| 96 | DRB1_0401 | AYFLDLTVKCDKP | 1 | YFLDLTVKC | 0.947 | 3ECA_A_PDBID_CH | 0.286146 | 4.80 | NA | <=WB |
| 127 | DRB1_0401 | FNLYNAVVTAADK | 3 | YNAVVTAAD | 0.887 | 3ECA_A_PDBID_CH | 0.528900 | 1.88 | NA | <=WB |
| 128 | DRB1_0401 | NLYNAVVTAADKA | 2 | YNAVVTAAD | 0.967 | 3ECA_A_PDBID_CH | 0.589305 | 1.44 | NA | <=WB |
| 212 | DRB1_0401 | PKVGIVYNYANAS | 4 | IVYNYANAS | 0.987 | 3ECA_A_PDBID_CH | 0.326994 | 4.06 | NA | <=WB |
| 214 | DRB1_0401 | VGIVYNYANASDL | 2 | IVYNYANAS | 0.987 | 3ECA_A_PDBID_CH | 0.516516 | 1.97 | NA | <=WB |
| 215 | DRB1_0401 | GIVYNYANASDLP | 1 | IVYNYANAS | 0.767 | 3ECA_A_PDBID_CH | 0.305171 | 4.43 | NA | <=WB |
| 216 | DRB1_0401 | IVYNYANASDLPA | 4 | YANASDLPA | 0.920 | 3ECA_A_PDBID_CH | 0.455856 | 2.47 | NA | <=WB |
| 219 | DRB1_0401 | NYANASDLPAKAL | 1 | YANASDLPA | 1.000 | 3ECA_A_PDBID_CH | 0.551685 | 1.71 | NA | <=WB |
| 247 | DRB1_0401 | GNLYKSVFDTLAT | 3 | YKSVFDTLA | 1.000 | 3ECA_A_PDBID_CH | 0.558782 | 1.66 | NA | <=WB |
| 248 | DRB1_0401 | NLYKSVFDTLATA | 2 | YKSVFDTLA | 1.000 | 3ECA_A_PDBID_CH | 0.407716 | 2.95 | NA | <=WB |
| 288 | DRB1_0401 | KYGFVASGTLNPQ | 3 | FVASGTLNP | 0.987 | 3ECA_A_PDBID_CH | 0.534775 | 1.84 | NA | <=WB |
| 289 | DRB1_0401 | YGFVASGTLNPQK | 2 | FVASGTLNP | 1.000 | 3ECA_A_PDBID_CH | 0.475145 | 2.31 | NA | <=WB |
| 38 | DRB1_0401 | AVPQLKDIANVKGE | 4 | LKDIANVKG | 1.000 | 3ECA_A_PDBID_CH | 0.530749 | 1.87 | NA | <=WB |
| 40 | DRB1_0401 | PQLKDIANVKGEQV | 2 | LKDIANVKG | 1.000 | 3ECA_A_PDBID_CH | 0.532987 | 1.85 | NA | <=WB |
| 93 | DRB1_0401 | EETAYFLDLTVKCD | 4 | YFLDLTVKC | 1.000 | 3ECA_A_PDBID_CH | 0.477869 | 2.29 | NA | <=WB |
| 95 | DRB1_0401 | TAYFLDLTVKCDKP | 2 | YFLDLTVKC | 0.973 | 3ECA_A_PDBID_CH | 0.615284 | 1.27 | NA | <=WB |
| 126 | DRB1_0401 | PFNLYNAVVTAADK | 4 | YNAVVTAAD | 0.627 | 3ECA_A_PDBID_CH | 0.566013 | 1.61 | NA | <=WB |
| 127 | DRB1_0401 | FNLYNAVVTAADKA | 3 | YNAVVTAAD | 0.913 | 3ECA_A_PDBID_CH | 0.630319 | 1.18 | NA | <=WB |
| 128 | DRB1_0401 | NLYNAVVTAADKAS | 2 | YNAVVTAAD | 0.993 | 3ECA_A_PDBID_CH | 0.624347 | 1.22 | NA | <=WB |
| 130 | DRB1_0401 | YNAVVTAADKASAN | 3 | VVTAADKAS | 0.813 | 3ECA_A_PDBID_CH | 0.313628 | 4.29 | NA | <=WB |
| 131 | DRB1_0401 | NAVVTAADKASANR | 2 | VVTAADKAS | 0.813 | 3ECA_A_PDBID_CH | 0.321282 | 4.16 | NA | <=WB |
| 144 | DRB1_0401 | RGVLVVMNDTVLDG | 3 | LVVMNDTVL | 0.680 | 3ECA_A_PDBID_CH | 0.392752 | 3.12 | NA | <=WB |
| 145 | DRB1_0401 | GVLVVMNDTVLDGR | 3 | VVMNDTVLD | 0.600 | 3ECA_A_PDBID_CH | 0.454569 | 2.48 | NA | <=WB |
| 146 | DRB1_0401 | VLVVMNDTVLDGRD | 2 | VVMNDTVLD | 0.540 | 3ECA_A_PDBID_CH | 0.369872 | 3.41 | NA | <=WB |

| | | | | | | | | | | |
|---|---|---|---|---|---|---|---|---|---|---|
| 184 | DRB1_0401 | NGKIDYQRTPARKH | 3 | IDYQRTPAR | 0.953 | 3ECA_A_PDBID_CH | 0.283986 | 4.84 | NA | <=WB |
| 214 | DRB1_0401 | VGIVYNYANASDLP | 2 | IVYNYANAS | 0.947 | 3ECA_A_PDBID_CH | 0.603462 | 1.34 | NA | <=WB |
| 215 | DRB1_0401 | GIVYNYANASDLPA | 5 | YANASDLPA | 0.613 | 3ECA_A_PDBID_CH | 0.434539 | 2.68 | NA | <=WB |
| 219 | DRB1_0401 | NYANASDLPAKALV | 1 | YANASDLPA | 0.993 | 3ECA_A_PDBID_CH | 0.406808 | 2.96 | NA | <=WB |
| 233 | DRB1_0401 | DAGYDGIVSAGVGN | 3 | YDGIVSAGV | 1.000 | 3ECA_A_PDBID_CH | 0.360469 | 3.54 | NA | <=WB |
| 246 | DRB1_0401 | NGNLYKSVFDTLAT | 4 | YKSVFDTLA | 1.000 | 3ECA_A_PDBID_CH | 0.474676 | 2.32 | NA | <=WB |
| 248 | DRB1_0401 | NLYKSVFDTLATAA | 2 | YKSVFDTLA | 1.000 | 3ECA_A_PDBID_CH | 0.428596 | 2.74 | NA | <=WB |
| 287 | DRB1_0401 | AKYGFVASGTLNPQ | 4 | FVASGTLNP | 0.967 | 3ECA_A_PDBID_CH | 0.478671 | 2.28 | NA | <=WB |
| 289 | DRB1_0401 | YGFVASGTLNPQKA | 2 | FVASGTLNP | 1.000 | 3ECA_A_PDBID_CH | 0.505138 | 2.06 | NA | <=WB |
| 37 | DRB1_0401 | NAVPQLKDIANVKGE | 5 | LKDIANVKG | 1.000 | 3ECA_A_PDBID_CH | 0.472183 | 2.34 | NA | <=WB |
| 40 | DRB1_0401 | PQLKDIANVKGEQVV | 2 | LKDIANVKG | 0.993 | 3ECA_A_PDBID_CH | 0.434920 | 2.68 | NA | <=WB |
| 92 | DRB1_0401 | MEETAYFLDLTVKCD | 5 | YFLDLTVKC | 0.993 | 3ECA_A_PDBID_CH | 0.404740 | 2.98 | NA | <=WB |
| 93 | DRB1_0401 | EETAYFLDLTVKCDK | 4 | YFLDLTVKC | 0.993 | 3ECA_A_PDBID_CH | 0.660361 | 1.02 | NA | <=WB |
| 95 | DRB1_0401 | TAYFLDLTVKCDKPV | 2 | YFLDLTVKC | 0.947 | 3ECA_A_PDBID_CH | 0.510534 | 2.02 | NA | <=WB |
| 112 | DRB1_0401 | VGAMRPSTSMSADGP | 3 | MRPSTSMSA | 1.000 | 3ECA_A_PDBID_CH | 0.333296 | 3.96 | NA | <=WB |
| 125 | DRB1_0401 | GPFNLYNAVVTAADK | 5 | YNAVVTAAD | 0.600 | 3ECA_A_PDBID_CH | 0.526624 | 1.90 | NA | <=WB |
| 128 | DRB1_0401 | NLYNAVVTAADKASA | 2 | YNAVVTAAD | 0.907 | 3ECA_A_PDBID_CH | 0.562629 | 1.63 | NA | <=WB |
| 129 | DRB1_0401 | LYNAVVTAADKASAN | 4 | VVTAADKAS | 0.667 | 3ECA_A_PDBID_CH | 0.352420 | 3.66 | NA | <=WB |
| 130 | DRB1_0401 | YNAVVTAADKASANR | 3 | VVTAADKAS | 0.900 | 3ECA_A_PDBID_CH | 0.448748 | 2.54 | NA | <=WB |
| 143 | DRB1_0401 | NRGVLVVMNDTVLDG | 4 | LVVMNDTVL | 0.627 | 3ECA_A_PDBID_CH | 0.361094 | 3.53 | NA | <=WB |
| 144 | DRB1_0401 | RGVLVVMNDTVLDGR | 3 | LVVMNDTVL | 0.633 | 3ECA_A_PDBID_CH | 0.565814 | 1.61 | NA | <=WB |
| 145 | DRB1_0401 | GVLVVMNDTVLDGRD | 3 | VVMNDTVLD | 0.707 | 3ECA_A_PDBID_CH | 0.525129 | 1.91 | NA | <=WB |
| 146 | DRB1_0401 | VLVVMNDTVLDGRDV | 3 | VMNDTVLDG | 0.567 | 3ECA_A_PDBID_CH | 0.297957 | 4.57 | NA | <=WB |
| 184 | DRB1_0401 | NGKIDYQRTPARKHT | 3 | IDYQRTPAR | 0.853 | 3ECA_A_PDBID_CH | 0.299151 | 4.54 | NA | <=WB |
| 211 | DRB1_0401 | LPKVGIVYNYANASD | 5 | IVYNYANAS | 0.993 | 3ECA_A_PDBID_CH | 0.619601 | 1.24 | NA | <=WB |
| 212 | DRB1_0401 | PKVGIVYNYANASDL | 4 | IVYNYANAS | 1.000 | 3ECA_A_PDBID_CH | 0.661767 | 1.01 | NA | <=WB |

| | | | | | | | | | | |
|---|---|---|---|---|---|---|---|---|---|---|
| 214 | DRB1_0401 | VGIVYNYANASDLPA | 2 | IVYNYANAS | 0.707 | 3ECA_A_PDBID_CH | 0.514557 | 1.99 | NA | <=WB |
| 219 | DRB1_0401 | NYANASDLPAKALVD | 1 | YANASDLPA | 0.967 | 3ECA_A_PDBID_CH | 0.287351 | 4.77 | NA | <=WB |
| 232 | DRB1_0401 | VDAGYDGIVSAGVGN | 4 | YDGIVSAGV | 1.000 | 3ECA_A_PDBID_CH | 0.370250 | 3.41 | NA | <=WB |
| 233 | DRB1_0401 | DAGYDGIVSAGVGNG | 3 | YDGIVSAGV | 0.993 | 3ECA_A_PDBID_CH | 0.465963 | 2.39 | NA | <=WB |
| 245 | DRB1_0401 | GNGNLYKSVFDTLAT | 5 | YKSVFDTLA | 1.000 | 3ECA_A_PDBID_CH | 0.391449 | 3.14 | NA | <=WB |
| 246 | DRB1_0401 | NGNLYKSVFDTLATA | 4 | YKSVFDTLA | 1.000 | 3ECA_A_PDBID_CH | 0.633199 | 1.16 | NA | <=WB |
| 248 | DRB1_0401 | NLYKSVFDTLATAAK | 2 | YKSVFDTLA | 0.947 | 3ECA_A_PDBID_CH | 0.311644 | 4.32 | NA | <=WB |
| 265 | DRB1_0401 | TAVVRSSRVPTGATT | 3 | VRSSRVPTG | 1.000 | 3ECA_A_PDBID_CH | 0.278164 | 4.95 | NA | <=WB |
| 286 | DRB1_0401 | DAKYGFVASGTLNPQ | 5 | FVASGTLNP | 0.807 | 3ECA_A_PDBID_CH | 0.417653 | 2.85 | NA | <=WB |
| 289 | DRB1_0401 | YGFVASGTLNPQKAR | 2 | FVASGTLNP | 0.987 | 3ECA_A_PDBID_CH | 0.365708 | 3.47 | NA | <=WB |

# Allele: DRB1_0701

| | | | | | | | | | | |
|---|---|---|---|---|---|---|---|---|---|---|
| 42 | DRB1_0701 | LKDIANVKGEQVV | 3 | IANVKGEQV | 1.000 | 3ECA_A_PDBID_CH | 0.712443 | 0.67 | NA | <=SB |
| 43 | DRB1_0701 | KDIANVKGEQVVN | 2 | IANVKGEQV | 1.000 | 3ECA_A_PDBID_CH | 0.706160 | 0.69 | NA | <=SB |
| 61 | DRB1_0701 | MNDNVWLTLAKKI | 4 | VWLTLAKKI | 1.000 | 3ECA_A_PDBID_CH | 0.665523 | 0.85 | NA | <=SB |
| 62 | DRB1_0701 | NDNVWLTLAKKIN | 3 | VWLTLAKKI | 1.000 | 3ECA_A_PDBID_CH | 0.853420 | 0.26 | NA | <=SB |
| 63 | DRB1_0701 | DNVWLTLAKKINT | 2 | VWLTLAKKI | 1.000 | 3ECA_A_PDBID_CH | 0.745309 | 0.56 | NA | <=SB |
| 80 | DRB1_0701 | TDGFVITHGTDTM | 3 | FVITHGTDT | 1.000 | 3ECA_A_PDBID_CH | 0.700966 | 0.71 | NA | <=SB |
| 215 | DRB1_0701 | GIVYNYANASDLP | 3 | YNYANASDL | 1.000 | 3ECA_A_PDBID_CH | 0.727702 | 0.62 | NA | <=SB |
| 233 | DRB1_0701 | DAGYDGIVSAGVG | 3 | YDGIVSAGV | 1.000 | 3ECA_A_PDBID_CH | 0.672671 | 0.82 | NA | <=SB |
| 286 | DRB1_0701 | DAKYGFVASGTLN | 3 | YGFVASGTL | 1.000 | 3ECA_A_PDBID_CH | 0.798901 | 0.41 | NA | <=SB |
| 287 | DRB1_0701 | AKYGFVASGTLNP | 2 | YGFVASGTL | 1.000 | 3ECA_A_PDBID_CH | 0.732577 | 0.60 | NA | <=SB |
| 41 | DRB1_0701 | QLKDIANVKGEQVV | 4 | IANVKGEQV | 1.000 | 3ECA_A_PDBID_CH | 0.707213 | 0.69 | NA | <=SB |
| 42 | DRB1_0701 | LKDIANVKGEQVVN | 3 | IANVKGEQV | 1.000 | 3ECA_A_PDBID_CH | 0.779984 | 0.46 | NA | <=SB |

| | | | | | | | | | | |
|---|---|---|---|---|---|---|---|---|---|---|
| 43 | DRB1_0701 | KDIANVKGEQVVNI | 2 | IANVKGEQV | 1.000 | 3ECA_A_PDBID_CH | 0.688739 | 0.76 | NA | <=SB |
| 61 | DRB1_0701 | MNDNVWLTLAKKIN | 4 | VWLTLAKKI | 1.000 | 3ECA_A_PDBID_CH | 0.841039 | 0.29 | NA | <=SB |
| 62 | DRB1_0701 | NDNVWLTLAKKINT | 3 | VWLTLAKKI | 1.000 | 3ECA_A_PDBID_CH | 0.867377 | 0.23 | NA | <=SB |
| 63 | DRB1_0701 | DNVWLTLAKKINTD | 2 | VWLTLAKKI | 1.000 | 3ECA_A_PDBID_CH | 0.804759 | 0.39 | NA | <=SB |
| 79 | DRB1_0701 | KTDGFVITHGTDTM | 4 | FVITHGTDT | 1.000 | 3ECA_A_PDBID_CH | 0.699313 | 0.72 | NA | <=SB |
| 80 | DRB1_0701 | TDGFVITHGTDTME | 3 | FVITHGTDT | 1.000 | 3ECA_A_PDBID_CH | 0.774179 | 0.47 | NA | <=SB |
| 81 | DRB1_0701 | DGFVITHGTDTMEE | 2 | FVITHGTDT | 1.000 | 3ECA_A_PDBID_CH | 0.675938 | 0.81 | NA | <=SB |
| 214 | DRB1_0701 | VGIVYNYANASDLP | 4 | YNYANASDL | 0.947 | 3ECA_A_PDBID_CH | 0.728071 | 0.62 | NA | <=SB |
| 215 | DRB1_0701 | GIVYNYANASDLPA | 3 | YNYANASDL | 1.000 | 3ECA_A_PDBID_CH | 0.786162 | 0.44 | NA | <=SB |
| 216 | DRB1_0701 | IVYNYANASDLPAK | 2 | YNYANASDL | 0.993 | 3ECA_A_PDBID_CH | 0.716844 | 0.65 | NA | <=SB |
| 232 | DRB1_0701 | VDAGYDGIVSAGVG | 4 | YDGIVSAGV | 1.000 | 3ECA_A_PDBID_CH | 0.666211 | 0.85 | NA | <=SB |
| 233 | DRB1_0701 | DAGYDGIVSAGVGN | 3 | YDGIVSAGV | 1.000 | 3ECA_A_PDBID_CH | 0.751984 | 0.54 | NA | <=SB |
| 285 | DRB1_0701 | DDAKYGFVASGTLN | 4 | YGFVASGTL | 1.000 | 3ECA_A_PDBID_CH | 0.786890 | 0.44 | NA | <=SB |
| 286 | DRB1_0701 | DAKYGFVASGTLNP | 3 | YGFVASGTL | 1.000 | 3ECA_A_PDBID_CH | 0.876850 | 0.21 | NA | <=SB |
| 287 | DRB1_0701 | AKYGFVASGTLNPQ | 2 | YGFVASGTL | 0.973 | 3ECA_A_PDBID_CH | 0.774316 | 0.47 | NA | <=SB |
| 40 | DRB1_0701 | PQLKDIANVKGEQVV | 5 | IANVKGEQV | 1.000 | 3ECA_A_PDBID_CH | 0.674838 | 0.82 | NA | <=SB |
| 41 | DRB1_0701 | QLKDIANVKGEQVVN | 4 | IANVKGEQV | 1.000 | 3ECA_A_PDBID_CH | 0.790399 | 0.43 | NA | <=SB |
| 42 | DRB1_0701 | LKDIANVKGEQVVNI | 3 | IANVKGEQV | 1.000 | 3ECA_A_PDBID_CH | 0.766696 | 0.49 | NA | <=SB |
| 60 | DRB1_0701 | DMNDNVWLTLAKKIN | 5 | VWLTLAKKI | 1.000 | 3ECA_A_PDBID_CH | 0.808590 | 0.38 | NA | <=SB |
| 61 | DRB1_0701 | MNDNVWLTLAKKINT | 4 | VWLTLAKKI | 1.000 | 3ECA_A_PDBID_CH | 0.863347 | 0.24 | NA | <=SB |
| 62 | DRB1_0701 | NDNVWLTLAKKINTD | 3 | VWLTLAKKI | 1.000 | 3ECA_A_PDBID_CH | 0.909089 | 0.14 | NA | <=SB |
| 63 | DRB1_0701 | DNVWLTLAKKINTDC | 2 | VWLTLAKKI | 1.000 | 3ECA_A_PDBID_CH | 0.738558 | 0.58 | NA | <=SB |
| 78 | DRB1_0701 | DKTDGFVITHGTDTM | 5 | FVITHGTDT | 1.000 | 3ECA_A_PDBID_CH | 0.664701 | 0.85 | NA | <=SB |
| 79 | DRB1_0701 | KTDGFVITHGTDTME | 4 | FVITHGTDT | 1.000 | 3ECA_A_PDBID_CH | 0.778442 | 0.46 | NA | <=SB |
| 80 | DRB1_0701 | TDGFVITHGTDTMEE | 3 | FVITHGTDT | 1.000 | 3ECA_A_PDBID_CH | 0.819944 | 0.35 | NA | <=SB |
| 213 | DRB1_0701 | KVGIVYNYANASDLP | 5 | YNYANASDL | 0.867 | 3ECA_A_PDBID_CH | 0.724649 | 0.63 | NA | <=SB |

| | | | | | | | | | | |
|---|---|---|---|---|---|---|---|---|---|---|
| 214 | DRB1_0701 | VGIVYNYANASDLPA | 4 | YNYANASDL | 0.987 | 3ECA_A_PDBID_CH | 0.802615 | 0.40 | NA | <=SB |
| 215 | DRB1_0701 | GIVYNYANASDLPAK | 3 | YNYANASDL | 1.000 | 3ECA_A_PDBID_CH | 0.865203 | 0.23 | NA | <=SB |
| 231 | DRB1_0701 | LVDAGYDGIVSAGVG | 5 | YDGIVSAGV | 1.000 | 3ECA_A_PDBID_CH | 0.685672 | 0.78 | NA | <=SB |
| 232 | DRB1_0701 | VDAGYDGIVSAGVGN | 4 | YDGIVSAGV | 1.000 | 3ECA_A_PDBID_CH | 0.797549 | 0.41 | NA | <=SB |
| 233 | DRB1_0701 | DAGYDGIVSAGVGNG | 3 | YDGIVSAGV | 1.000 | 3ECA_A_PDBID_CH | 0.827671 | 0.33 | NA | <=SB |
| 284 | DRB1_0701 | VDDAKYGFVASGTLN | 5 | YGFVASGTL | 1.000 | 3ECA_A_PDBID_CH | 0.761717 | 0.51 | NA | <=SB |
| 285 | DRB1_0701 | DDAKYGFVASGTLNP | 4 | YGFVASGTL | 1.000 | 3ECA_A_PDBID_CH | 0.884281 | 0.20 | NA | <=SB |
| 286 | DRB1_0701 | DAKYGFVASGTLNPQ | 3 | YGFVASGTL | 1.000 | 3ECA_A_PDBID_CH | 0.909081 | 0.14 | NA | <=SB |
| 287 | DRB1_0701 | AKYGFVASGTLNPQK | 2 | YGFVASGTL | 0.887 | 3ECA_A_PDBID_CH | 0.719580 | 0.65 | NA | <=SB |
| 63 | DRB1_0701 | DNVWLTLAKKI | 2 | VWLTLAKKI | 1.000 | 3ECA_A_PDBID_CH | 0.248919 | 4.79 | NA | <=WB |
| 42 | DRB1_0701 | LKDIANVKGEQV | 3 | IANVKGEQV | 1.000 | 3ECA_A_PDBID_CH | 0.389642 | 2.71 | NA | <=WB |
| 43 | DRB1_0701 | KDIANVKGEQVV | 2 | IANVKGEQV | 1.000 | 3ECA_A_PDBID_CH | 0.512511 | 1.69 | NA | <=WB |
| 62 | DRB1_0701 | NDNVWLTLAKKI | 3 | VWLTLAKKI | 1.000 | 3ECA_A_PDBID_CH | 0.565667 | 1.37 | NA | <=WB |
| 63 | DRB1_0701 | DNVWLTLAKKIN | 2 | VWLTLAKKI | 1.000 | 3ECA_A_PDBID_CH | 0.605028 | 1.14 | NA | <=WB |
| 64 | DRB1_0701 | NVWLTLAKKINT | 1 | VWLTLAKKI | 1.000 | 3ECA_A_PDBID_CH | 0.242034 | 4.93 | NA | <=WB |
| 80 | DRB1_0701 | TDGFVITHGTDT | 3 | FVITHGTDT | 1.000 | 3ECA_A_PDBID_CH | 0.469179 | 2.00 | NA | <=WB |
| 81 | DRB1_0701 | DGFVITHGTDTM | 2 | FVITHGTDT | 1.000 | 3ECA_A_PDBID_CH | 0.411896 | 2.48 | NA | <=WB |
| 215 | DRB1_0701 | GIVYNYANASDL | 3 | YNYANASDL | 0.953 | 3ECA_A_PDBID_CH | 0.290386 | 4.03 | NA | <=WB |
| 216 | DRB1_0701 | IVYNYANASDLP | 2 | YNYANASDL | 1.000 | 3ECA_A_PDBID_CH | 0.371573 | 2.90 | NA | <=WB |
| 233 | DRB1_0701 | DAGYDGIVSAGV | 3 | YDGIVSAGV | 1.000 | 3ECA_A_PDBID_CH | 0.278445 | 4.24 | NA | <=WB |
| 234 | DRB1_0701 | AGYDGIVSAGVG | 2 | YDGIVSAGV | 1.000 | 3ECA_A_PDBID_CH | 0.305089 | 3.80 | NA | <=WB |
| 286 | DRB1_0701 | DAKYGFVASGTL | 3 | YGFVASGTL | 1.000 | 3ECA_A_PDBID_CH | 0.390455 | 2.70 | NA | <=WB |
| 287 | DRB1_0701 | AKYGFVASGTLN | 2 | YGFVASGTL | 1.000 | 3ECA_A_PDBID_CH | 0.445933 | 2.20 | NA | <=WB |
| 3 | DRB1_0701 | NITILATGGTIAG | 3 | ILATGGTIA | 0.993 | 3ECA_A_PDBID_CH | 0.299446 | 3.89 | NA | <=WB |
| 41 | DRB1_0701 | QLKDIANVKGEQV | 4 | IANVKGEQV | 1.000 | 3ECA_A_PDBID_CH | 0.512210 | 1.69 | NA | <=WB |
| 44 | DRB1_0701 | DIANVKGEQVVNI | 1 | IANVKGEQV | 0.960 | 3ECA_A_PDBID_CH | 0.243349 | 4.90 | NA | <=WB |

| | | | | | | | | | | |
|---|---|---|---|---|---|---|---|---|---|---|
| 64 | DRB1_0701 | NVWLTLAKKINTD | 1 | VWLTLAKKI | 1.000 | 3ECA_A_PDBID_CH | 0.453146 | 2.14 | NA | <=WB |
| 79 | DRB1_0701 | KTDGFVITHGTDT | 4 | FVITHGTDT | 1.000 | 3ECA_A_PDBID_CH | 0.584234 | 1.26 | NA | <=WB |
| 81 | DRB1_0701 | DGFVITHGTDTME | 2 | FVITHGTDT | 1.000 | 3ECA_A_PDBID_CH | 0.617517 | 1.07 | NA | <=WB |
| 82 | DRB1_0701 | GFVITHGTDTMEE | 1 | FVITHGTDT | 1.000 | 3ECA_A_PDBID_CH | 0.281681 | 4.18 | NA | <=WB |
| 112 | DRB1_0701 | VGAMRPSTSMSAD | 3 | MRPSTSMSA | 1.000 | 3ECA_A_PDBID_CH | 0.301500 | 3.86 | NA | <=WB |
| 127 | DRB1_0701 | FNLYNAVVTAADK | 3 | YNAVVTAAD | 0.933 | 3ECA_A_PDBID_CH | 0.328074 | 3.46 | NA | <=WB |
| 128 | DRB1_0701 | NLYNAVVTAADKA | 2 | YNAVVTAAD | 0.953 | 3ECA_A_PDBID_CH | 0.341037 | 3.28 | NA | <=WB |
| 157 | DRB1_0701 | GRDVTKTNTTDVA | 3 | VTKTNTTDV | 1.000 | 3ECA_A_PDBID_CH | 0.343739 | 3.25 | NA | <=WB |
| 176 | DRB1_0701 | YGPLGYIHNGKID | 3 | LGYIHNGKI | 1.000 | 3ECA_A_PDBID_CH | 0.397489 | 2.62 | NA | <=WB |
| 177 | DRB1_0701 | GPLGYIHNGKIDY | 2 | LGYIHNGKI | 0.987 | 3ECA_A_PDBID_CH | 0.308939 | 3.75 | NA | <=WB |
| 185 | DRB1_0701 | GKIDYQRTPARKH | 4 | YQRTPARKH | 0.960 | 3ECA_A_PDBID_CH | 0.272242 | 4.34 | NA | <=WB |
| 186 | DRB1_0701 | KIDYQRTPARKHT | 3 | YQRTPARKH | 1.000 | 3ECA_A_PDBID_CH | 0.390310 | 2.70 | NA | <=WB |
| 187 | DRB1_0701 | IDYQRTPARKHTS | 2 | YQRTPARKH | 1.000 | 3ECA_A_PDBID_CH | 0.252354 | 4.72 | NA | <=WB |
| 200 | DRB1_0701 | DTPFDVSKLNELP | 3 | FDVSKLNEL | 1.000 | 3ECA_A_PDBID_CH | 0.349988 | 3.16 | NA | <=WB |
| 201 | DRB1_0701 | TPFDVSKLNELPK | 2 | FDVSKLNEL | 1.000 | 3ECA_A_PDBID_CH | 0.282173 | 4.17 | NA | <=WB |
| 213 | DRB1_0701 | KVGIVYNYANASD | 3 | IVYNYANAS | 0.987 | 3ECA_A_PDBID_CH | 0.240914 | 4.95 | NA | <=WB |
| 214 | DRB1_0701 | VGIVYNYANASDL | 4 | YNYANASDL | 0.880 | 3ECA_A_PDBID_CH | 0.442759 | 2.22 | NA | <=WB |
| 216 | DRB1_0701 | IVYNYANASDLPA | 2 | YNYANASDL | 1.000 | 3ECA_A_PDBID_CH | 0.612015 | 1.10 | NA | <=WB |
| 217 | DRB1_0701 | VYNYANASDLPAK | 1 | YNYANASDL | 0.833 | 3ECA_A_PDBID_CH | 0.373666 | 2.88 | NA | <=WB |
| 232 | DRB1_0701 | VDAGYDGIVSAGV | 4 | YDGIVSAGV | 1.000 | 3ECA_A_PDBID_CH | 0.401027 | 2.59 | NA | <=WB |
| 234 | DRB1_0701 | AGYDGIVSAGVGN | 2 | YDGIVSAGV | 1.000 | 3ECA_A_PDBID_CH | 0.546725 | 1.47 | NA | <=WB |
| 285 | DRB1_0701 | DDAKYGFVASGTL | 4 | YGFVASGTL | 1.000 | 3ECA_A_PDBID_CH | 0.520377 | 1.64 | NA | <=WB |
| 288 | DRB1_0701 | KYGFVASGTLNPQ | 1 | YGFVASGTL | 0.753 | 3ECA_A_PDBID_CH | 0.442341 | 2.23 | NA | <=WB |
| 2 | DRB1_0701 | PNITILATGGTIAG | 4 | ILATGGTIA | 0.967 | 3ECA_A_PDBID_CH | 0.280080 | 4.21 | NA | <=WB |
| 3 | DRB1_0701 | NITILATGGTIAGG | 3 | ILATGGTIA | 0.987 | 3ECA_A_PDBID_CH | 0.370175 | 2.91 | NA | <=WB |
| 40 | DRB1_0701 | PQLKDIANVKGEQV | 5 | IANVKGEQV | 0.993 | 3ECA_A_PDBID_CH | 0.447518 | 2.18 | NA | <=WB |

| | | | | | | | | | | |
|---|---|---|---|---|---|---|---|---|---|---|
| 60 | DRB1_0701 | DMNDNVWLTLAKKI | 5 | VWLTLAKKI | 1.000 | 3ECA_A_PDBID_CH | 0.592580 | 1.20 | NA | <=WB |
| 64 | DRB1_0701 | NVWLTLAKKINTDC | 1 | VWLTLAKKI | 1.000 | 3ECA_A_PDBID_CH | 0.333043 | 3.39 | NA | <=WB |
| 78 | DRB1_0701 | DKTDGFVITHGTDT | 5 | FVITHGTDT | 1.000 | 3ECA_A_PDBID_CH | 0.524814 | 1.62 | NA | <=WB |
| 111 | DRB1_0701 | MVGAMRPSTSMSAD | 4 | MRPSTSMSA | 1.000 | 3ECA_A_PDBID_CH | 0.288332 | 4.07 | NA | <=WB |
| 112 | DRB1_0701 | VGAMRPSTSMSADG | 3 | MRPSTSMSA | 1.000 | 3ECA_A_PDBID_CH | 0.378213 | 2.83 | NA | <=WB |
| 113 | DRB1_0701 | GAMRPSTSMSADGP | 2 | MRPSTSMSA | 0.993 | 3ECA_A_PDBID_CH | 0.303119 | 3.83 | NA | <=WB |
| 126 | DRB1_0701 | PFNLYNAVVTAADK | 4 | YNAVVTAAD | 0.807 | 3ECA_A_PDBID_CH | 0.315092 | 3.65 | NA | <=WB |
| 127 | DRB1_0701 | FNLYNAVVTAADKA | 3 | YNAVVTAAD | 0.953 | 3ECA_A_PDBID_CH | 0.395714 | 2.64 | NA | <=WB |
| 128 | DRB1_0701 | NLYNAVVTAADKAS | 2 | YNAVVTAAD | 0.953 | 3ECA_A_PDBID_CH | 0.368699 | 2.93 | NA | <=WB |
| 156 | DRB1_0701 | DGRDVTKTNTTDVA | 4 | VTKTNTTDV | 1.000 | 3ECA_A_PDBID_CH | 0.333816 | 3.38 | NA | <=WB |
| 157 | DRB1_0701 | GRDVTKTNTTDVAT | 3 | VTKTNTTDV | 1.000 | 3ECA_A_PDBID_CH | 0.400437 | 2.59 | NA | <=WB |
| 165 | DRB1_0701 | TTDVATFKSVNYGP | 3 | VATFKSVNY | 0.993 | 3ECA_A_PDBID_CH | 0.266777 | 4.44 | NA | <=WB |
| 175 | DRB1_0701 | NYGPLGYIHNGKID | 4 | LGYIHNGKI | 1.000 | 3ECA_A_PDBID_CH | 0.385308 | 2.75 | NA | <=WB |
| 176 | DRB1_0701 | YGPLGYIHNGKIDY | 3 | LGYIHNGKI | 1.000 | 3ECA_A_PDBID_CH | 0.416118 | 2.44 | NA | <=WB |
| 177 | DRB1_0701 | GPLGYIHNGKIDYQ | 2 | LGYIHNGKI | 0.987 | 3ECA_A_PDBID_CH | 0.339867 | 3.30 | NA | <=WB |
| 185 | DRB1_0701 | GKIDYQRTPARKHT | 4 | YQRTPARKH | 0.967 | 3ECA_A_PDBID_CH | 0.374103 | 2.87 | NA | <=WB |
| 186 | DRB1_0701 | KIDYQRTPARKHTS | 3 | YQRTPARKH | 1.000 | 3ECA_A_PDBID_CH | 0.411950 | 2.48 | NA | <=WB |
| 187 | DRB1_0701 | IDYQRTPARKHTSD | 2 | YQRTPARKH | 1.000 | 3ECA_A_PDBID_CH | 0.302471 | 3.84 | NA | <=WB |
| 199 | DRB1_0701 | SDTPFDVSKLNELP | 4 | FDVSKLNEL | 1.000 | 3ECA_A_PDBID_CH | 0.344300 | 3.24 | NA | <=WB |
| 200 | DRB1_0701 | DTPFDVSKLNELPK | 3 | FDVSKLNEL | 1.000 | 3ECA_A_PDBID_CH | 0.471167 | 1.99 | NA | <=WB |
| 201 | DRB1_0701 | TPFDVSKLNELPKV | 2 | FDVSKLNEL | 1.000 | 3ECA_A_PDBID_CH | 0.272067 | 4.35 | NA | <=WB |
| 213 | DRB1_0701 | KVGIVYNYANASDL | 5 | YNYANASDL | 0.660 | 3ECA_A_PDBID_CH | 0.422519 | 2.39 | NA | <=WB |
| 217 | DRB1_0701 | VYNYANASDLPAKA | 1 | YNYANASDL | 0.653 | 3ECA_A_PDBID_CH | 0.311991 | 3.70 | NA | <=WB |
| 231 | DRB1_0701 | LVDAGYDGIVSAGV | 5 | YDGIVSAGV | 1.000 | 3ECA_A_PDBID_CH | 0.330752 | 3.42 | NA | <=WB |
| 234 | DRB1_0701 | AGYDGIVSAGVGNG | 2 | YDGIVSAGV | 1.000 | 3ECA_A_PDBID_CH | 0.588434 | 1.23 | NA | <=WB |
| 284 | DRB1_0701 | VDDAKYGFVASGTL | 5 | YGFVASGTL | 1.000 | 3ECA_A_PDBID_CH | 0.430511 | 2.32 | NA | <=WB |

| | | | | | | | | | | |
|---|---|---|---|---|---|---|---|---|---|---|
| 288 | DRB1_0701 | KYGFVASGTLNPQK | 1 | YGFVASGTL | 0.533 | 3ECA_A_PDBID_CH | 0.392252 | 2.68 | NA | <=WB |
| 1 | DRB1_0701 | LPNITILATGGTIAG | 5 | ILATGGTIA | 0.953 | 3ECA_A_PDBID_CH | 0.261920 | 4.52 | NA | <=WB |
| 2 | DRB1_0701 | PNITILATGGTIAGG | 4 | ILATGGTIA | 0.973 | 3ECA_A_PDBID_CH | 0.401303 | 2.58 | NA | <=WB |
| 3 | DRB1_0701 | NITILATGGTIAGGG | 3 | ILATGGTIA | 1.000 | 3ECA_A_PDBID_CH | 0.461918 | 2.06 | NA | <=WB |
| 39 | DRB1_0701 | VPQLKDIANVKGEQV | 6 | IANVKGEQV | 0.973 | 3ECA_A_PDBID_CH | 0.394537 | 2.66 | NA | <=WB |
| 43 | DRB1_0701 | KDIANVKGEQVVNIG | 2 | IANVKGEQV | 0.993 | 3ECA_A_PDBID_CH | 0.610748 | 1.11 | NA | <=WB |
| 50 | DRB1_0701 | GEQVVNIGSQDMNDN | 3 | VVNIGSQDM | 0.987 | 3ECA_A_PDBID_CH | 0.258517 | 4.59 | NA | <=WB |
| 59 | DRB1_0701 | QDMNDNVWLTLAKKI | 6 | VWLTLAKKI | 1.000 | 3ECA_A_PDBID_CH | 0.512699 | 1.69 | NA | <=WB |
| 64 | DRB1_0701 | NVWLTLAKKINTDCD | 1 | VWLTLAKKI | 1.000 | 3ECA_A_PDBID_CH | 0.261120 | 4.54 | NA | <=WB |
| 77 | DRB1_0701 | CDKTDGFVITHGTDT | 6 | FVITHGTDT | 1.000 | 3ECA_A_PDBID_CH | 0.454399 | 2.13 | NA | <=WB |
| 81 | DRB1_0701 | DGFVITHGTDTMEET | 2 | FVITHGTDT | 1.000 | 3ECA_A_PDBID_CH | 0.602218 | 1.15 | NA | <=WB |
| 110 | DRB1_0701 | VMVGAMRPSTSMSAD | 5 | MRPSTSMSA | 0.987 | 3ECA_A_PDBID_CH | 0.253941 | 4.69 | NA | <=WB |
| 111 | DRB1_0701 | MVGAMRPSTSMSADG | 4 | MRPSTSMSA | 1.000 | 3ECA_A_PDBID_CH | 0.388613 | 2.72 | NA | <=WB |
| 112 | DRB1_0701 | VGAMRPSTSMSADGP | 3 | MRPSTSMSA | 1.000 | 3ECA_A_PDBID_CH | 0.498589 | 1.80 | NA | <=WB |
| 113 | DRB1_0701 | GAMRPSTSMSADGPF | 2 | MRPSTSMSA | 0.993 | 3ECA_A_PDBID_CH | 0.245624 | 4.86 | NA | <=WB |
| 123 | DRB1_0701 | ADGPFNLYNAVVTAA | 4 | FNLYNAVVT | 0.993 | 3ECA_A_PDBID_CH | 0.245882 | 4.85 | NA | <=WB |
| 124 | DRB1_0701 | DGPFNLYNAVVTAAD | 3 | FNLYNAVVT | 0.887 | 3ECA_A_PDBID_CH | 0.367421 | 2.94 | NA | <=WB |
| 125 | DRB1_0701 | GPFNLYNAVVTAADK | 5 | YNAVVTAAD | 0.627 | 3ECA_A_PDBID_CH | 0.334752 | 3.37 | NA | <=WB |
| 126 | DRB1_0701 | PFNLYNAVVTAADKA | 4 | YNAVVTAAD | 0.887 | 3ECA_A_PDBID_CH | 0.423410 | 2.38 | NA | <=WB |
| 127 | DRB1_0701 | FNLYNAVVTAADKAS | 3 | YNAVVTAAD | 0.953 | 3ECA_A_PDBID_CH | 0.462859 | 2.06 | NA | <=WB |
| 128 | DRB1_0701 | NLYNAVVTAADKASA | 2 | YNAVVTAAD | 0.933 | 3ECA_A_PDBID_CH | 0.320465 | 3.57 | NA | <=WB |
| 155 | DRB1_0701 | LDGRDVTKTNTTDVA | 5 | VTKTNTTDV | 0.993 | 3ECA_A_PDBID_CH | 0.325953 | 3.49 | NA | <=WB |
| 156 | DRB1_0701 | DGRDVTKTNTTDVAT | 4 | VTKTNTTDV | 1.000 | 3ECA_A_PDBID_CH | 0.435618 | 2.28 | NA | <=WB |
| 157 | DRB1_0701 | GRDVTKTNTTDVATF | 3 | VTKTNTTDV | 1.000 | 3ECA_A_PDBID_CH | 0.427199 | 2.35 | NA | <=WB |
| 164 | DRB1_0701 | NTTDVATFKSVNYGP | 4 | VATFKSVNY | 0.993 | 3ECA_A_PDBID_CH | 0.281528 | 4.18 | NA | <=WB |
| 165 | DRB1_0701 | TTDVATFKSVNYGPL | 3 | VATFKSVNY | 0.793 | 3ECA_A_PDBID_CH | 0.258512 | 4.59 | NA | <=WB |

| | | | | | | | | | | |
|---|---|---|---|---|---|---|---|---|---|---|
| 174 | DRB1_0701 | VNYGPLGYIHNGKID | 5 | LGYIHNGKI | 0.993 | 3ECA_A_PDBID_CH | 0.357957 | 3.06 | NA | <=WB |
| 175 | DRB1_0701 | NYGPLGYIHNGKIDY | 4 | LGYIHNGKI | 1.000 | 3ECA_A_PDBID_CH | 0.424361 | 2.37 | NA | <=WB |
| 176 | DRB1_0701 | YGPLGYIHNGKIDYQ | 3 | LGYIHNGKI | 1.000 | 3ECA_A_PDBID_CH | 0.469305 | 2.00 | NA | <=WB |
| 177 | DRB1_0701 | GPLGYIHNGKIDYQR | 2 | LGYIHNGKI | 0.960 | 3ECA_A_PDBID_CH | 0.276477 | 4.27 | NA | <=WB |
| 184 | DRB1_0701 | NGKIDYQRTPARKHT | 5 | YQRTPARKH | 0.947 | 3ECA_A_PDBID_CH | 0.340392 | 3.29 | NA | <=WB |
| 185 | DRB1_0701 | GKIDYQRTPARKHTS | 4 | YQRTPARKH | 0.993 | 3ECA_A_PDBID_CH | 0.407740 | 2.51 | NA | <=WB |
| 186 | DRB1_0701 | KIDYQRTPARKHTSD | 3 | YQRTPARKH | 1.000 | 3ECA_A_PDBID_CH | 0.497606 | 1.81 | NA | <=WB |
| 187 | DRB1_0701 | IDYQRTPARKHTSDT | 2 | YQRTPARKH | 1.000 | 3ECA_A_PDBID_CH | 0.243646 | 4.90 | NA | <=WB |
| 198 | DRB1_0701 | TSDTPFDVSKLNELP | 5 | FDVSKLNEL | 0.993 | 3ECA_A_PDBID_CH | 0.312669 | 3.69 | NA | <=WB |
| 199 | DRB1_0701 | SDTPFDVSKLNELPK | 4 | FDVSKLNEL | 1.000 | 3ECA_A_PDBID_CH | 0.479037 | 1.93 | NA | <=WB |
| 200 | DRB1_0701 | DTPFDVSKLNELPKV | 3 | FDVSKLNEL | 1.000 | 3ECA_A_PDBID_CH | 0.482865 | 1.90 | NA | <=WB |
| 212 | DRB1_0701 | PKVGIVYNYANASDL | 6 | YNYANASDL | 0.647 | 3ECA_A_PDBID_CH | 0.362950 | 2.99 | NA | <=WB |
| 216 | DRB1_0701 | IVYNYANASDLPAKA | 2 | YNYANASDL | 0.933 | 3ECA_A_PDBID_CH | 0.629302 | 1.01 | NA | <=WB |
| 217 | DRB1_0701 | VYNYANASDLPAKAL | 1 | YNYANASDL | 0.633 | 3ECA_A_PDBID_CH | 0.245904 | 4.85 | NA | <=WB |
| 230 | DRB1_0701 | ALVDAGYDGIVSAGV | 6 | YDGIVSAGV | 1.000 | 3ECA_A_PDBID_CH | 0.310335 | 3.72 | NA | <=WB |
| 234 | DRB1_0701 | AGYDGIVSAGVGNGN | 2 | YDGIVSAGV | 0.993 | 3ECA_A_PDBID_CH | 0.551129 | 1.45 | NA | <=WB |
| 261 | DRB1_0701 | AKTGTAVVRSSRVPT | 4 | TAVVRSSRV | 0.993 | 3ECA_A_PDBID_CH | 0.254231 | 4.68 | NA | <=WB |
| 262 | DRB1_0701 | KTGTAVVRSSRVPTG | 3 | TAVVRSSRV | 0.987 | 3ECA_A_PDBID_CH | 0.310394 | 3.72 | NA | <=WB |
| 283 | DRB1_0701 | EVDDAKYGFVASGTL | 6 | YGFVASGTL | 1.000 | 3ECA_A_PDBID_CH | 0.386416 | 2.74 | NA | <=WB |
| 288 | DRB1_0701 | KYGFVASGTLNPQKA | 3 | FVASGTLNP | 0.640 | 3ECA_A_PDBID_CH | 0.393850 | 2.66 | NA | <=WB |

# Allele: DRB1_0801

| | | | | | | | | | | |
|---|---|---|---|---|---|---|---|---|---|---|
| 127 | DRB1_0801 | FNLYNAVVTAADK | 3 | YNAVVTAAD | 1.000 | 3ECA_A_PDBID_CH | 0.816422 | 0.53 | NA | <=SB |
| 128 | DRB1_0801 | NLYNAVVTAADKA | 2 | YNAVVTAAD | 1.000 | 3ECA_A_PDBID_CH | 0.855517 | 0.35 | NA | <=SB |

| | | | | | | | | | | |
|---|---|---|---|---|---|---|---|---|---|---|
| 126 | DRB1_0801 | PFNLYNAVVTAADK | 4 | YNAVVTAAD | 0.987 | 3ECA_A_PDBID_CH | 0.794670 | 0.65 | NA | <=SB |
| 127 | DRB1_0801 | FNLYNAVVTAADKA | 3 | YNAVVTAAD | 1.000 | 3ECA_A_PDBID_CH | 0.865691 | 0.31 | NA | <=SB |
| 128 | DRB1_0801 | NLYNAVVTAADKAS | 2 | YNAVVTAAD | 0.993 | 3ECA_A_PDBID_CH | 0.875745 | 0.27 | NA | <=SB |
| 125 | DRB1_0801 | GPFNLYNAVVTAADK | 5 | YNAVVTAAD | 0.987 | 3ECA_A_PDBID_CH | 0.757895 | 0.86 | NA | <=SB |
| 126 | DRB1_0801 | PFNLYNAVVTAADKA | 4 | YNAVVTAAD | 0.987 | 3ECA_A_PDBID_CH | 0.858004 | 0.34 | NA | <=SB |
| 127 | DRB1_0801 | FNLYNAVVTAADKAS | 3 | YNAVVTAAD | 1.000 | 3ECA_A_PDBID_CH | 0.889852 | 0.22 | NA | <=SB |
| 128 | DRB1_0801 | NLYNAVVTAADKASA | 2 | YNAVVTAAD | 0.993 | 3ECA_A_PDBID_CH | 0.832305 | 0.45 | NA | <=SB |
| 128 | DRB1_0801 | NLYNAVVTAADK | 2 | YNAVVTAAD | 1.000 | 3ECA_A_PDBID_CH | 0.696661 | 1.33 | NA | <=WB |
| 129 | DRB1_0801 | LYNAVVTAADKA | 1 | YNAVVTAAD | 1.000 | 3ECA_A_PDBID_CH | 0.477379 | 3.93 | NA | <=WB |
| 22 | DRB1_0801 | KSNYTVGKVGVEN | 3 | YTVGKVGVE | 0.947 | 3ECA_A_PDBID_CH | 0.423505 | 5.00 | NA | <=WB |
| 63 | DRB1_0801 | DNVWLTLAKKINT | 3 | WLTLAKKIN | 0.967 | 3ECA_A_PDBID_CH | 0.611940 | 2.11 | NA | <=WB |
| 64 | DRB1_0801 | NVWLTLAKKINTD | 2 | WLTLAKKIN | 0.740 | 3ECA_A_PDBID_CH | 0.575930 | 2.51 | NA | <=WB |
| 129 | DRB1_0801 | LYNAVVTAADKAS | 1 | YNAVVTAAD | 0.993 | 3ECA_A_PDBID_CH | 0.633457 | 1.88 | NA | <=WB |
| 213 | DRB1_0801 | KVGIVYNYANASD | 3 | IVYNYANAS | 0.920 | 3ECA_A_PDBID_CH | 0.456524 | 4.32 | NA | <=WB |
| 33 | DRB1_0801 | ENLVNAVPQLKDIA | 3 | VNAVPQLKD | 0.967 | 3ECA_A_PDBID_CH | 0.525880 | 3.17 | NA | <=WB |
| 62 | DRB1_0801 | NDNVWLTLAKKINT | 4 | WLTLAKKIN | 0.920 | 3ECA_A_PDBID_CH | 0.576310 | 2.50 | NA | <=WB |
| 63 | DRB1_0801 | DNVWLTLAKKINTD | 3 | WLTLAKKIN | 0.873 | 3ECA_A_PDBID_CH | 0.715579 | 1.17 | NA | <=WB |
| 129 | DRB1_0801 | LYNAVVTAADKASA | 1 | YNAVVTAAD | 0.847 | 3ECA_A_PDBID_CH | 0.561338 | 2.69 | NA | <=WB |
| 131 | DRB1_0801 | NAVVTAADKASANR | 3 | VTAADKASA | 0.973 | 3ECA_A_PDBID_CH | 0.539697 | 2.97 | NA | <=WB |
| 212 | DRB1_0801 | PKVGIVYNYANASD | 4 | IVYNYANAS | 0.900 | 3ECA_A_PDBID_CH | 0.442398 | 4.60 | NA | <=WB |
| 213 | DRB1_0801 | KVGIVYNYANASDL | 3 | IVYNYANAS | 0.873 | 3ECA_A_PDBID_CH | 0.438295 | 4.69 | NA | <=WB |
| 32 | DRB1_0801 | VENLVNAVPQLKDIA | 4 | VNAVPQLKD | 0.960 | 3ECA_A_PDBID_CH | 0.506812 | 3.46 | NA | <=WB |
| 33 | DRB1_0801 | ENLVNAVPQLKDIAN | 3 | VNAVPQLKD | 1.000 | 3ECA_A_PDBID_CH | 0.577867 | 2.48 | NA | <=WB |
| 61 | DRB1_0801 | MNDNVWLTLAKKINT | 5 | WLTLAKKIN | 0.907 | 3ECA_A_PDBID_CH | 0.486842 | 3.78 | NA | <=WB |
| 62 | DRB1_0801 | NDNVWLTLAKKINTD | 4 | WLTLAKKIN | 0.873 | 3ECA_A_PDBID_CH | 0.687441 | 1.40 | NA | <=WB |
| 63 | DRB1_0801 | DNVWLTLAKKINTDC | 3 | WLTLAKKIN | 0.833 | 3ECA_A_PDBID_CH | 0.509373 | 3.42 | NA | <=WB |

| | | | | | | | | | | |
|---|---|---|---|---|---|---|---|---|---|---|
| 129 | DRB1_0801 | LYNAVVTAADKASAN | 1 | YNAVVTAAD | 0.587 | 3ECA_A_PDBID_CH | 0.550730 | 2.83 | NA | <=WB |
| 130 | DRB1_0801 | YNAVVTAADKASANR | 4 | VTAADKASA | 0.953 | 3ECA_A_PDBID_CH | 0.514749 | 3.34 | NA | <=WB |
| 131 | DRB1_0801 | NAVVTAADKASANRG | 3 | VTAADKASA | 0.993 | 3ECA_A_PDBID_CH | 0.571167 | 2.57 | NA | <=WB |
| 145 | DRB1_0801 | GVLVVMNDTVLDGRD | 3 | VVMNDTVLD | 0.967 | 3ECA_A_PDBID_CH | 0.462530 | 4.21 | NA | <=WB |
| 213 | DRB1_0801 | KVGIVYNYANASDLP | 3 | IVYNYANAS | 0.820 | 3ECA_A_PDBID_CH | 0.503477 | 3.51 | NA | <=WB |

# Allele: DRB1_1101

| | | | | | | | | | | |
|---|---|---|---|---|---|---|---|---|---|---|
| 63 | DRB1_1101 | DNVWLTLAKKINTD | 3 | WLTLAKKIN | 0.907 | 3ECA_A_PDBID_CH | 0.821043 | 0.84 | NA | <=SB |
| 131 | DRB1_1101 | NAVVTAADKASANRG | 3 | VTAADKASA | 1.000 | 3ECA_A_PDBID_CH | 0.832963 | 0.77 | NA | <=SB |
| 63 | DRB1_1101 | DNVWLTLAKKINT | 3 | WLTLAKKIN | 0.993 | 3ECA_A_PDBID_CH | 0.686725 | 1.61 | NA | <=WB |
| 64 | DRB1_1101 | NVWLTLAKKINTD | 2 | WLTLAKKIN | 0.720 | 3ECA_A_PDBID_CH | 0.689332 | 1.59 | NA | <=WB |
| 131 | DRB1_1101 | NAVVTAADKASAN | 3 | VTAADKASA | 1.000 | 3ECA_A_PDBID_CH | 0.511718 | 2.95 | NA | <=WB |
| 132 | DRB1_1101 | AVVTAADKASANR | 2 | VTAADKASA | 1.000 | 3ECA_A_PDBID_CH | 0.516289 | 2.91 | NA | <=WB |
| 178 | DRB1_1101 | PLGYIHNGKIDYQ | 3 | YIHNGKIDY | 0.987 | 3ECA_A_PDBID_CH | 0.377640 | 4.50 | NA | <=WB |
| 213 | DRB1_1101 | KVGIVYNYANASD | 3 | IVYNYANAS | 0.993 | 3ECA_A_PDBID_CH | 0.470140 | 3.40 | NA | <=WB |
| 264 | DRB1_1101 | GTAVVRSSRVPTG | 3 | VVRSSRVPT | 1.000 | 3ECA_A_PDBID_CH | 0.407132 | 4.14 | NA | <=WB |
| 62 | DRB1_1101 | NDNVWLTLAKKINT | 4 | WLTLAKKIN | 0.987 | 3ECA_A_PDBID_CH | 0.643721 | 1.88 | NA | <=WB |
| 64 | DRB1_1101 | NVWLTLAKKINTDC | 2 | WLTLAKKIN | 0.620 | 3ECA_A_PDBID_CH | 0.459464 | 3.52 | NA | <=WB |
| 130 | DRB1_1101 | YNAVVTAADKASAN | 4 | VTAADKASA | 0.987 | 3ECA_A_PDBID_CH | 0.517292 | 2.90 | NA | <=WB |
| 131 | DRB1_1101 | NAVVTAADKASANR | 3 | VTAADKASA | 1.000 | 3ECA_A_PDBID_CH | 0.762907 | 1.16 | NA | <=WB |
| 132 | DRB1_1101 | AVVTAADKASANRG | 2 | VTAADKASA | 1.000 | 3ECA_A_PDBID_CH | 0.633116 | 1.95 | NA | <=WB |
| 177 | DRB1_1101 | GPLGYIHNGKIDYQ | 4 | YIHNGKIDY | 0.907 | 3ECA_A_PDBID_CH | 0.363575 | 4.72 | NA | <=WB |
| 178 | DRB1_1101 | PLGYIHNGKIDYQR | 3 | YIHNGKIDY | 0.993 | 3ECA_A_PDBID_CH | 0.551048 | 2.60 | NA | <=WB |
| 212 | DRB1_1101 | PKVGIVYNYANASD | 4 | IVYNYANAS | 0.993 | 3ECA_A_PDBID_CH | 0.455360 | 3.57 | NA | <=WB |

| | | | | | | | | | | |
|---|---|---|---|---|---|---|---|---|---|---|
| 213 | DRB1_1101 | KVGIVYNYANASDL | 3 | IVYNYANAS | 0.987 | 3ECA_A_PDBID_CH | 0.479862 | 3.29 | NA | <=WB |
| 263 | DRB1_1101 | TGTAVVRSSRVPTG | 4 | VVRSSRVPT | 1.000 | 3ECA_A_PDBID_CH | 0.386141 | 4.39 | NA | <=WB |
| 264 | DRB1_1101 | GTAVVRSSRVPTGA | 3 | VVRSSRVPT | 0.993 | 3ECA_A_PDBID_CH | 0.572548 | 2.42 | NA | <=WB |
| 61 | DRB1_1101 | MNDNVWLTLAKKINT | 5 | WLTLAKKIN | 0.993 | 3ECA_A_PDBID_CH | 0.552126 | 2.59 | NA | <=WB |
| 62 | DRB1_1101 | NDNVWLTLAKKINTD | 4 | WLTLAKKIN | 0.973 | 3ECA_A_PDBID_CH | 0.789760 | 1.02 | NA | <=WB |
| 63 | DRB1_1101 | DNVWLTLAKKINTDC | 3 | WLTLAKKIN | 0.873 | 3ECA_A_PDBID_CH | 0.638302 | 1.91 | NA | <=WB |
| 64 | DRB1_1101 | NVWLTLAKKINTDCD | 3 | LTLAKKINT | 0.653 | 3ECA_A_PDBID_CH | 0.427659 | 3.89 | NA | <=WB |
| 127 | DRB1_1101 | FNLYNAVVTAADKAS | 3 | YNAVVTAAD | 0.993 | 3ECA_A_PDBID_CH | 0.370538 | 4.61 | NA | <=WB |
| 129 | DRB1_1101 | LYNAVVTAADKASAN | 5 | VTAADKASA | 0.953 | 3ECA_A_PDBID_CH | 0.469117 | 3.41 | NA | <=WB |
| 130 | DRB1_1101 | YNAVVTAADKASANR | 4 | VTAADKASA | 1.000 | 3ECA_A_PDBID_CH | 0.766093 | 1.14 | NA | <=WB |
| 132 | DRB1_1101 | AVVTAADKASANRGV | 2 | VTAADKASA | 1.000 | 3ECA_A_PDBID_CH | 0.537141 | 2.72 | NA | <=WB |
| 177 | DRB1_1101 | GPLGYIHNGKIDYQR | 4 | YIHNGKIDY | 0.960 | 3ECA_A_PDBID_CH | 0.500025 | 3.07 | NA | <=WB |
| 178 | DRB1_1101 | PLGYIHNGKIDYQRT | 3 | YIHNGKIDY | 0.993 | 3ECA_A_PDBID_CH | 0.525776 | 2.82 | NA | <=WB |
| 186 | DRB1_1101 | KIDYQRTPARKHTSD | 3 | YQRTPARKH | 1.000 | 3ECA_A_PDBID_CH | 0.385684 | 4.40 | NA | <=WB |
| 211 | DRB1_1101 | LPKVGIVYNYANASD | 5 | IVYNYANAS | 0.987 | 3ECA_A_PDBID_CH | 0.379786 | 4.47 | NA | <=WB |
| 212 | DRB1_1101 | PKVGIVYNYANASDL | 4 | IVYNYANAS | 1.000 | 3ECA_A_PDBID_CH | 0.440919 | 3.74 | NA | <=WB |
| 213 | DRB1_1101 | KVGIVYNYANASDLP | 3 | IVYNYANAS | 0.993 | 3ECA_A_PDBID_CH | 0.566517 | 2.47 | NA | <=WB |
| 263 | DRB1_1101 | TGTAVVRSSRVPTGA | 4 | VVRSSRVPT | 1.000 | 3ECA_A_PDBID_CH | 0.519380 | 2.88 | NA | <=WB |
| 264 | DRB1_1101 | GTAVVRSSRVPTGAT | 3 | VVRSSRVPT | 0.980 | 3ECA_A_PDBID_CH | 0.564436 | 2.48 | NA | <=WB |

**# Allele: DRB1_1301**

| | | | | | | | | | | |
|---|---|---|---|---|---|---|---|---|---|---|
| 184 | DRB1_1301 | NGKIDYQRTPARKH | 3 | IDYQRTPAR | 0.987 | 3ECA_A_PDBID_CH | 0.756006 | 0.90 | NA | <=SB |
| 183 | DRB1_1301 | HNGKIDYQRTPARKH | 4 | IDYQRTPAR | 0.973 | 3ECA_A_PDBID_CH | 0.749549 | 0.94 | NA | <=SB |
| 184 | DRB1_1301 | NGKIDYQRTPARKHT | 3 | IDYQRTPAR | 0.973 | 3ECA_A_PDBID_CH | 0.770490 | 0.81 | NA | <=SB |
| 165 | DRB1_1301 | TTDVATFKSVNYG | 3 | VATFKSVNY | 1.000 | 3ECA_A_PDBID_CH | 0.495974 | 3.74 | NA | <=WB |

| | | | | | | | | | | | |
|---|---|---|---|---|---|---|---|---|---|---|---|
| 166 | DRB1_1301 | TDVATFKSVNYGP | 2 | VATFKSVNY | 0.993 | 3ECA_A_PDBID_CH | 0.480626 | 3.99 | NA | <=WB |
| 184 | DRB1_1301 | NGKIDYQRTPARK | 3 | IDYQRTPAR | 0.993 | 3ECA_A_PDBID_CH | 0.656866 | 1.71 | NA | <=WB |
| 185 | DRB1_1301 | GKIDYQRTPARKH | 2 | IDYQRTPAR | 0.987 | 3ECA_A_PDBID_CH | 0.623093 | 2.09 | NA | <=WB |
| 213 | DRB1_1301 | KVGIVYNYANASD | 3 | IVYNYANAS | 0.987 | 3ECA_A_PDBID_CH | 0.565993 | 2.73 | NA | <=WB |
| 264 | DRB1_1301 | GTAVVRSSRVPTG | 3 | VVRSSRVPT | 0.987 | 3ECA_A_PDBID_CH | 0.448059 | 4.59 | NA | <=WB |
| 131 | DRB1_1301 | NAVVTAADKASANR | 3 | VTAADKASA | 0.980 | 3ECA_A_PDBID_CH | 0.578727 | 2.58 | NA | <=WB |
| 132 | DRB1_1301 | AVVTAADKASANRG | 2 | VTAADKASA | 0.980 | 3ECA_A_PDBID_CH | 0.457973 | 4.40 | NA | <=WB |
| 164 | DRB1_1301 | NTTDVATFKSVNYG | 4 | VATFKSVNY | 1.000 | 3ECA_A_PDBID_CH | 0.470290 | 4.18 | NA | <=WB |
| 165 | DRB1_1301 | TTDVATFKSVNYGP | 3 | VATFKSVNY | 0.993 | 3ECA_A_PDBID_CH | 0.621143 | 2.11 | NA | <=WB |
| 183 | DRB1_1301 | HNGKIDYQRTPARK | 4 | IDYQRTPAR | 0.980 | 3ECA_A_PDBID_CH | 0.662992 | 1.65 | NA | <=WB |
| 185 | DRB1_1301 | GKIDYQRTPARKHT | 2 | IDYQRTPAR | 0.980 | 3ECA_A_PDBID_CH | 0.661536 | 1.66 | NA | <=WB |
| 212 | DRB1_1301 | PKVGIVYNYANASD | 4 | IVYNYANAS | 0.967 | 3ECA_A_PDBID_CH | 0.554197 | 2.88 | NA | <=WB |
| 213 | DRB1_1301 | KVGIVYNYANASDL | 3 | IVYNYANAS | 0.973 | 3ECA_A_PDBID_CH | 0.576071 | 2.61 | NA | <=WB |
| 214 | DRB1_1301 | VGIVYNYANASDLP | 2 | IVYNYANAS | 0.947 | 3ECA_A_PDBID_CH | 0.452728 | 4.50 | NA | <=WB |
| 264 | DRB1_1301 | GTAVVRSSRVPTGA | 3 | VVRSSRVPT | 0.987 | 3ECA_A_PDBID_CH | 0.563172 | 2.77 | NA | <=WB |
| 130 | DRB1_1301 | YNAVVTAADKASANR | 4 | VTAADKASA | 0.960 | 3ECA_A_PDBID_CH | 0.608891 | 2.24 | NA | <=WB |
| 131 | DRB1_1301 | NAVVTAADKASANRG | 3 | VTAADKASA | 0.987 | 3ECA_A_PDBID_CH | 0.684887 | 1.44 | NA | <=WB |
| 163 | DRB1_1301 | TNTTDVATFKSVNYG | 5 | VATFKSVNY | 0.993 | 3ECA_A_PDBID_CH | 0.453859 | 4.48 | NA | <=WB |
| 164 | DRB1_1301 | NTTDVATFKSVNYGP | 4 | VATFKSVNY | 1.000 | 3ECA_A_PDBID_CH | 0.648380 | 1.81 | NA | <=WB |
| 165 | DRB1_1301 | TTDVATFKSVNYGPL | 3 | VATFKSVNY | 0.993 | 3ECA_A_PDBID_CH | 0.600451 | 2.33 | NA | <=WB |
| 182 | DRB1_1301 | IHNGKIDYQRTPARK | 5 | IDYQRTPAR | 0.980 | 3ECA_A_PDBID_CH | 0.636649 | 1.94 | NA | <=WB |
| 185 | DRB1_1301 | GKIDYQRTPARKHTS | 2 | IDYQRTPAR | 0.960 | 3ECA_A_PDBID_CH | 0.607344 | 2.26 | NA | <=WB |
| 211 | DRB1_1301 | LPKVGIVYNYANASD | 5 | IVYNYANAS | 0.947 | 3ECA_A_PDBID_CH | 0.502494 | 3.63 | NA | <=WB |
| 212 | DRB1_1301 | PKVGIVYNYANASDL | 4 | IVYNYANAS | 0.973 | 3ECA_A_PDBID_CH | 0.550376 | 2.92 | NA | <=WB |
| 213 | DRB1_1301 | KVGIVYNYANASDLP | 3 | IVYNYANAS | 0.980 | 3ECA_A_PDBID_CH | 0.646161 | 1.84 | NA | <=WB |
| 263 | DRB1_1301 | TGTAVVRSSRVPTGA | 4 | VVRSSRVPT | 0.980 | 3ECA_A_PDBID_CH | 0.529964 | 3.21 | NA | <=WB |

| | | | | | | | | | | |
|---|---|---|---|---|---|---|---|---|---|---|
| 264 | DRB1_1301 | GTAVVRSSRVPTGAT | 3 | VVRSSRVPT | 0.973 | 3ECA_A_PDBID_CH | 0.571609 | 2.66 | NA | <=WB |

# Allele: DRB1_1501

| | | | | | | | | | | |
|---|---|---|---|---|---|---|---|---|---|---|
| 42 | DRB1_1501 | LKDIANVKGEQVVN | 3 | IANVKGEQV | 1.000 | 3ECA_A_PDBID_CH | 0.731120 | 0.72 | NA | <=SB |
| 165 | DRB1_1501 | TTDVATFKSVNYGP | 3 | VATFKSVNY | 1.000 | 3ECA_A_PDBID_CH | 0.673522 | 0.90 | NA | <=SB |
| 41 | DRB1_1501 | QLKDIANVKGEQVVN | 4 | IANVKGEQV | 1.000 | 3ECA_A_PDBID_CH | 0.715774 | 0.76 | NA | <=SB |
| 42 | DRB1_1501 | LKDIANVKGEQVVNI | 3 | IANVKGEQV | 1.000 | 3ECA_A_PDBID_CH | 0.709645 | 0.78 | NA | <=SB |
| 124 | DRB1_1501 | DGPFNLYNAVVTAAD | 3 | FNLYNAVVT | 1.000 | 3ECA_A_PDBID_CH | 0.690907 | 0.84 | NA | <=SB |
| 144 | DRB1_1501 | RGVLVVMNDTVLDGR | 3 | LVVMNDTVL | 0.960 | 3ECA_A_PDBID_CH | 0.694990 | 0.82 | NA | <=SB |
| 164 | DRB1_1501 | NTTDVATFKSVNYGP | 4 | VATFKSVNY | 1.000 | 3ECA_A_PDBID_CH | 0.658345 | 0.96 | NA | <=SB |
| 176 | DRB1_1501 | YGPLGYIHNGKIDYQ | 3 | LGYIHNGKI | 1.000 | 3ECA_A_PDBID_CH | 0.650172 | 0.99 | NA | <=SB |
| 43 | DRB1_1501 | KDIANVKGEQVV | 2 | IANVKGEQV | 0.993 | 3ECA_A_PDBID_CH | 0.313248 | 3.15 | NA | <=WB |
| 125 | DRB1_1501 | GPFNLYNAVVTA | 2 | FNLYNAVVT | 1.000 | 3ECA_A_PDBID_CH | 0.249286 | 3.98 | NA | <=WB |
| 166 | DRB1_1501 | TDVATFKSVNYG | 2 | VATFKSVNY | 1.000 | 3ECA_A_PDBID_CH | 0.247827 | 4.00 | NA | <=WB |
| 177 | DRB1_1501 | GPLGYIHNGKID | 2 | LGYIHNGKI | 0.973 | 3ECA_A_PDBID_CH | 0.205518 | 4.87 | NA | <=WB |
| 42 | DRB1_1501 | LKDIANVKGEQVV | 3 | IANVKGEQV | 1.000 | 3ECA_A_PDBID_CH | 0.570010 | 1.34 | NA | <=WB |
| 43 | DRB1_1501 | KDIANVKGEQVVN | 2 | IANVKGEQV | 1.000 | 3ECA_A_PDBID_CH | 0.638603 | 1.04 | NA | <=WB |
| 123 | DRB1_1501 | ADGPFNLYNAVVT | 4 | FNLYNAVVT | 0.973 | 3ECA_A_PDBID_CH | 0.219501 | 4.58 | NA | <=WB |
| 124 | DRB1_1501 | DGPFNLYNAVVTA | 3 | FNLYNAVVT | 1.000 | 3ECA_A_PDBID_CH | 0.555055 | 1.41 | NA | <=WB |
| 125 | DRB1_1501 | GPFNLYNAVVTAA | 2 | FNLYNAVVT | 1.000 | 3ECA_A_PDBID_CH | 0.465939 | 1.88 | NA | <=WB |
| 126 | DRB1_1501 | PFNLYNAVVTAAD | 1 | FNLYNAVVT | 0.993 | 3ECA_A_PDBID_CH | 0.208326 | 4.81 | NA | <=WB |
| 144 | DRB1_1501 | RGVLVVMNDTVLD | 3 | LVVMNDTVL | 0.907 | 3ECA_A_PDBID_CH | 0.443797 | 2.02 | NA | <=WB |
| 145 | DRB1_1501 | GVLVVMNDTVLDG | 2 | LVVMNDTVL | 0.940 | 3ECA_A_PDBID_CH | 0.323788 | 3.03 | NA | <=WB |
| 165 | DRB1_1501 | TTDVATFKSVNYG | 3 | VATFKSVNY | 1.000 | 3ECA_A_PDBID_CH | 0.532377 | 1.51 | NA | <=WB |

| | | | | | | | | | | |
|---|---|---|---|---|---|---|---|---|---|---|
| 166 | DRB1_1501 | TDVATFKSVNYGP | 2 | VATFKSVNY | 1.000 | 3ECA_A_PDBID_CH | 0.514772 | 1.61 | NA | <=WB |
| 176 | DRB1_1501 | YGPLGYIHNGKID | 3 | LGYIHNGKI | 0.993 | 3ECA_A_PDBID_CH | 0.447847 | 1.99 | NA | <=WB |
| 177 | DRB1_1501 | GPLGYIHNGKIDY | 2 | LGYIHNGKI | 1.000 | 3ECA_A_PDBID_CH | 0.454826 | 1.95 | NA | <=WB |
| 213 | DRB1_1501 | KVGIVYNYANASD | 3 | IVYNYANAS | 0.620 | 3ECA_A_PDBID_CH | 0.239978 | 4.16 | NA | <=WB |
| 214 | DRB1_1501 | VGIVYNYANASDL | 3 | VYNYANASD | 0.733 | 3ECA_A_PDBID_CH | 0.265123 | 3.76 | NA | <=WB |
| 215 | DRB1_1501 | GIVYNYANASDLP | 2 | VYNYANASD | 0.887 | 3ECA_A_PDBID_CH | 0.257497 | 3.87 | NA | <=WB |
| 39 | DRB1_1501 | VPQLKDIANVKGEQ | 3 | LKDIANVKG | 1.000 | 3ECA_A_PDBID_CH | 0.317961 | 3.10 | NA | <=WB |
| 40 | DRB1_1501 | PQLKDIANVKGEQV | 2 | LKDIANVKG | 0.693 | 3ECA_A_PDBID_CH | 0.234792 | 4.27 | NA | <=WB |
| 41 | DRB1_1501 | QLKDIANVKGEQVV | 4 | IANVKGEQV | 0.993 | 3ECA_A_PDBID_CH | 0.544961 | 1.46 | NA | <=WB |
| 43 | DRB1_1501 | KDIANVKGEQVVNI | 2 | IANVKGEQV | 1.000 | 3ECA_A_PDBID_CH | 0.627026 | 1.09 | NA | <=WB |
| 50 | DRB1_1501 | GEQVVNIGSQDMND | 3 | VVNIGSQDM | 1.000 | 3ECA_A_PDBID_CH | 0.287652 | 3.45 | NA | <=WB |
| 123 | DRB1_1501 | ADGPFNLYNAVVTA | 4 | FNLYNAVVT | 0.987 | 3ECA_A_PDBID_CH | 0.520855 | 1.57 | NA | <=WB |
| 124 | DRB1_1501 | DGPFNLYNAVVTAA | 3 | FNLYNAVVT | 1.000 | 3ECA_A_PDBID_CH | 0.627178 | 1.09 | NA | <=WB |
| 125 | DRB1_1501 | GPFNLYNAVVTAAD | 2 | FNLYNAVVT | 1.000 | 3ECA_A_PDBID_CH | 0.546174 | 1.45 | NA | <=WB |
| 143 | DRB1_1501 | NRGVLVVMNDTVLD | 4 | LVVMNDTVL | 0.747 | 3ECA_A_PDBID_CH | 0.463977 | 1.89 | NA | <=WB |
| 144 | DRB1_1501 | RGVLVVMNDTVLDG | 3 | LVVMNDTVL | 0.907 | 3ECA_A_PDBID_CH | 0.581248 | 1.29 | NA | <=WB |
| 145 | DRB1_1501 | GVLVVMNDTVLDGR | 2 | LVVMNDTVL | 0.953 | 3ECA_A_PDBID_CH | 0.462374 | 1.90 | NA | <=WB |
| 164 | DRB1_1501 | NTTDVATFKSVNYG | 4 | VATFKSVNY | 1.000 | 3ECA_A_PDBID_CH | 0.491604 | 1.73 | NA | <=WB |
| 166 | DRB1_1501 | TDVATFKSVNYGPL | 2 | VATFKSVNY | 1.000 | 3ECA_A_PDBID_CH | 0.425956 | 2.16 | NA | <=WB |
| 175 | DRB1_1501 | NYGPLGYIHNGKID | 4 | LGYIHNGKI | 0.980 | 3ECA_A_PDBID_CH | 0.419418 | 2.21 | NA | <=WB |
| 176 | DRB1_1501 | YGPLGYIHNGKIDY | 3 | LGYIHNGKI | 1.000 | 3ECA_A_PDBID_CH | 0.571267 | 1.33 | NA | <=WB |
| 177 | DRB1_1501 | GPLGYIHNGKIDYQ | 2 | LGYIHNGKI | 1.000 | 3ECA_A_PDBID_CH | 0.533160 | 1.51 | NA | <=WB |
| 184 | DRB1_1501 | NGKIDYQRTPARKH | 3 | IDYQRTPAR | 0.993 | 3ECA_A_PDBID_CH | 0.206038 | 4.86 | NA | <=WB |
| 212 | DRB1_1501 | PKVGIVYNYANASD | 4 | IVYNYANAS | 0.520 | 3ECA_A_PDBID_CH | 0.257007 | 3.87 | NA | <=WB |
| 213 | DRB1_1501 | KVGIVYNYANASDL | 4 | VYNYANASD | 0.533 | 3ECA_A_PDBID_CH | 0.329602 | 2.97 | NA | <=WB |
| 214 | DRB1_1501 | VGIVYNYANASDLP | 3 | VYNYANASD | 0.773 | 3ECA_A_PDBID_CH | 0.404756 | 2.32 | NA | <=WB |

| | | | | | | | | | | |
|---|---|---|---|---|---|---|---|---|---|---|
| 215 | DRB1_1501 | GIVYNYANASDLPA | 2 | VYNYANASD | 0.940 | 3ECA_A_PDBID_CH | 0.326109 | 3.00 | NA | <=WB |
| 38 | DRB1_1501 | AVPQLKDIANVKGEQ | 4 | LKDIANVKG | 1.000 | 3ECA_A_PDBID_CH | 0.327560 | 2.99 | NA | <=WB |
| 39 | DRB1_1501 | VPQLKDIANVKGEQV | 3 | LKDIANVKG | 0.913 | 3ECA_A_PDBID_CH | 0.369752 | 2.60 | NA | <=WB |
| 40 | DRB1_1501 | PQLKDIANVKGEQVV | 5 | IANVKGEQV | 0.867 | 3ECA_A_PDBID_CH | 0.486444 | 1.76 | NA | <=WB |
| 43 | DRB1_1501 | KDIANVKGEQVVNIG | 2 | IANVKGEQV | 1.000 | 3ECA_A_PDBID_CH | 0.524740 | 1.55 | NA | <=WB |
| 49 | DRB1_1501 | KGEQVVNIGSQDMND | 4 | VVNIGSQDM | 1.000 | 3ECA_A_PDBID_CH | 0.292557 | 3.40 | NA | <=WB |
| 50 | DRB1_1501 | GEQVVNIGSQDMNDN | 3 | VVNIGSQDM | 1.000 | 3ECA_A_PDBID_CH | 0.405126 | 2.32 | NA | <=WB |
| 122 | DRB1_1501 | SADGPFNLYNAVVTA | 5 | FNLYNAVVT | 0.987 | 3ECA_A_PDBID_CH | 0.442219 | 2.03 | NA | <=WB |
| 123 | DRB1_1501 | ADGPFNLYNAVVTAA | 4 | FNLYNAVVT | 1.000 | 3ECA_A_PDBID_CH | 0.587291 | 1.26 | NA | <=WB |
| 125 | DRB1_1501 | GPFNLYNAVVTAADK | 2 | FNLYNAVVT | 0.993 | 3ECA_A_PDBID_CH | 0.430430 | 2.12 | NA | <=WB |
| 142 | DRB1_1501 | ANRGVLVVMNDTVLD | 5 | LVVMNDTVL | 0.753 | 3ECA_A_PDBID_CH | 0.414541 | 2.25 | NA | <=WB |
| 143 | DRB1_1501 | NRGVLVVMNDTVLDG | 4 | LVVMNDTVL | 0.780 | 3ECA_A_PDBID_CH | 0.609664 | 1.16 | NA | <=WB |
| 145 | DRB1_1501 | GVLVVMNDTVLDGRD | 2 | LVVMNDTVL | 0.953 | 3ECA_A_PDBID_CH | 0.378788 | 2.52 | NA | <=WB |
| 163 | DRB1_1501 | TNTTDVATFKSVNYG | 5 | VATFKSVNY | 1.000 | 3ECA_A_PDBID_CH | 0.424914 | 2.17 | NA | <=WB |
| 165 | DRB1_1501 | TTDVATFKSVNYGPL | 3 | VATFKSVNY | 1.000 | 3ECA_A_PDBID_CH | 0.609214 | 1.16 | NA | <=WB |
| 166 | DRB1_1501 | TDVATFKSVNYGPLG | 2 | VATFKSVNY | 1.000 | 3ECA_A_PDBID_CH | 0.317866 | 3.10 | NA | <=WB |
| 174 | DRB1_1501 | VNYGPLGYIHNGKID | 5 | LGYIHNGKI | 0.987 | 3ECA_A_PDBID_CH | 0.341831 | 2.86 | NA | <=WB |
| 175 | DRB1_1501 | NYGPLGYIHNGKIDY | 4 | LGYIHNGKI | 0.993 | 3ECA_A_PDBID_CH | 0.540256 | 1.48 | NA | <=WB |
| 177 | DRB1_1501 | GPLGYIHNGKIDYQR | 2 | LGYIHNGKI | 1.000 | 3ECA_A_PDBID_CH | 0.420828 | 2.20 | NA | <=WB |
| 184 | DRB1_1501 | NGKIDYQRTPARKHT | 3 | IDYQRTPAR | 0.987 | 3ECA_A_PDBID_CH | 0.217859 | 4.61 | NA | <=WB |
| 211 | DRB1_1501 | LPKVGIVYNYANASD | 3 | VGIVYNYAN | 0.413 | 3ECA_A_PDBID_CH | 0.244185 | 4.08 | NA | <=WB |
| 212 | DRB1_1501 | PKVGIVYNYANASDL | 5 | VYNYANASD | 0.440 | 3ECA_A_PDBID_CH | 0.298246 | 3.33 | NA | <=WB |
| 213 | DRB1_1501 | KVGIVYNYANASDLP | 4 | VYNYANASD | 0.633 | 3ECA_A_PDBID_CH | 0.469879 | 1.86 | NA | <=WB |
| 214 | DRB1_1501 | VGIVYNYANASDLPA | 3 | VYNYANASD | 0.933 | 3ECA_A_PDBID_CH | 0.497247 | 1.70 | NA | <=WB |
| 215 | DRB1_1501 | GIVYNYANASDLPAK | 2 | VYNYANASD | 0.873 | 3ECA_A_PDBID_CH | 0.280540 | 3.54 | NA | <=WB |

Table S2. Output of the NetMHCIIpan version 4.0 program. for the 4O0H protein. Of a total of 2086 fragmented peptides, only those that bind to MHCII either weakly or strongly and are therefore considered antigenic are presented.

```
# NetMHCIIpan version 4.0

# Input is in FASTA format

# Peptide length 9,10,11,12,13,14,15

# Prediction Mode: EL

# Threshold for Strong binding peptides (%Rank)1%
# Threshold for Weak binding peptides (%Rank) 5%

# Allele: DRB1_0101
```

| Pos | MHC | Peptide | Of | Core | Core_Rel | Identity | Score_EL | %Rank_EL | Exp_Bind | BindLevel |
|---|---|---|---|---|---|---|---|---|---|---|
| 235 | DRB1_0101 | TLFHIEQGKTVE | 2 | FHIEQGKTV | 1.000 | 4O0H_PDBID | 0.794961 | 0.90 | NA | <=SB |
| 234 | DRB1_0101 | LTLFHIEQGKTVE | 3 | FHIEQGKTV | 1.000 | 4O0H_PDBID | 0.921956 | 0.33 | NA | <=SB |
| 235 | DRB1_0101 | TLFHIEQGKTVEE | 2 | FHIEQGKTV | 1.000 | 4O0H_PDBID | 0.947656 | 0.21 | NA | <=SB |
| 236 | DRB1_0101 | LFHIEQGKTVEEA | 1 | FHIEQGKTV | 1.000 | 4O0H_PDBID | 0.779704 | 0.97 | NA | <=SB |
| 233 | DRB1_0101 | RLTLFHIEQGKTVE | 4 | FHIEQGKTV | 1.000 | 4O0H_PDBID | 0.907577 | 0.39 | NA | <=SB |
| 234 | DRB1_0101 | LTLFHIEQGKTVEE | 3 | FHIEQGKTV | 1.000 | 4O0H_PDBID | 0.963580 | 0.13 | NA | <=SB |
| 235 | DRB1_0101 | TLFHIEQGKTVEEA | 2 | FHIEQGKTV | 1.000 | 4O0H_PDBID | 0.965539 | 0.12 | NA | <=SB |
| 232 | DRB1_0101 | ARLTLFHIEQGKTVE | 5 | FHIEQGKTV | 1.000 | 4O0H_PDBID | 0.844460 | 0.67 | NA | <=SB |
| 233 | DRB1_0101 | RLTLFHIEQGKTVEE | 4 | FHIEQGKTV | 1.000 | 4O0H_PDBID | 0.950899 | 0.20 | NA | <=SB |
| 234 | DRB1_0101 | LTLFHIEQGKTVEEA | 3 | FHIEQGKTV | 1.000 | 4O0H_PDBID | 0.968406 | 0.11 | NA | <=SB |
| 235 | DRB1_0101 | TLFHIEQGKTVEEAA | 2 | FHIEQGKTV | 1.000 | 4O0H_PDBID | 0.932854 | 0.28 | NA | <=SB |
| 257 | DRB1_0101 | KSRVKGLGGLIVVSK | 3 | VKGLGGLIV | 1.000 | 4O0H_PDBID | 0.843803 | 0.67 | NA | <=SB |
| 3 | DRB1_0101 | NPIVVVHGGGAG | 2 | IVVVHGGGA | 1.000 | 4O0H_PDBID | 0.266913 | 4.98 | NA | <=WB |
| 234 | DRB1_0101 | LTLFHIEQGKTV | 3 | FHIEQGKTV | 1.000 | 4O0H_PDBID | 0.321740 | 4.24 | NA | <=WB |
| 236 | DRB1_0101 | LFHIEQGKTVEE | 1 | FHIEQGKTV | 1.000 | 4O0H_PDBID | 0.540090 | 2.26 | NA | <=WB |

| | | | | | | | | | | |
|---|---|---|---|---|---|---|---|---|---|---|
| 2 | DRB1_0101 | MNPIVVVHGGGAG | 3 | IVVVHGGGA | 1.000 | 4O0H_PDBID | 0.523037 | 2.37 | NA | <=WB |
| 3 | DRB1_0101 | NPIVVVHGGGAGP | 2 | IVVVHGGGA | 1.000 | 4O0H_PDBID | 0.626272 | 1.73 | NA | <=WB |
| 35 | DRB1_0101 | GYGILREGGSAVD | 3 | ILREGGSAV | 0.973 | 4O0H_PDBID | 0.375503 | 3.65 | NA | <=WB |
| 36 | DRB1_0101 | YGILREGGSAVDA | 2 | ILREGGSAV | 1.000 | 4O0H_PDBID | 0.345526 | 3.96 | NA | <=WB |
| 105 | DRB1_0101 | LARLVMEKTPHCF | 3 | LVMEKTPHC | 1.000 | 4O0H_PDBID | 0.335812 | 4.07 | NA | <=WB |
| 106 | DRB1_0101 | ARLVMEKTPHCFL | 2 | LVMEKTPHC | 1.000 | 4O0H_PDBID | 0.298515 | 4.52 | NA | <=WB |
| 233 | DRB1_0101 | RLTLFHIEQGKTV | 4 | FHIEQGKTV | 1.000 | 4O0H_PDBID | 0.467340 | 2.81 | NA | <=WB |
| 257 | DRB1_0101 | KSRVKGLGGLIVV | 3 | VKGLGGLIV | 1.000 | 4O0H_PDBID | 0.605052 | 1.84 | NA | <=WB |
| 258 | DRB1_0101 | SRVKGLGGLIVVS | 2 | VKGLGGLIV | 1.000 | 4O0H_PDBID | 0.451764 | 2.94 | NA | <=WB |
| 1 | DRB1_0101 | HMNPIVVVHGGGAG | 4 | IVVVHGGGA | 1.000 | 4O0H_PDBID | 0.484795 | 2.66 | NA | <=WB |
| 2 | DRB1_0101 | MNPIVVVHGGGAGP | 3 | IVVVHGGGA | 1.000 | 4O0H_PDBID | 0.720307 | 1.24 | NA | <=WB |
| 3 | DRB1_0101 | NPIVVVHGGGAGPI | 2 | IVVVHGGGA | 1.000 | 4O0H_PDBID | 0.610306 | 1.82 | NA | <=WB |
| 34 | DRB1_0101 | VGYGILREGGSAVD | 4 | ILREGGSAV | 0.847 | 4O0H_PDBID | 0.364254 | 3.77 | NA | <=WB |
| 35 | DRB1_0101 | GYGILREGGSAVDA | 3 | ILREGGSAV | 0.993 | 4O0H_PDBID | 0.611734 | 1.81 | NA | <=WB |
| 36 | DRB1_0101 | YGILREGGSAVDAV | 2 | ILREGGSAV | 1.000 | 4O0H_PDBID | 0.359834 | 3.81 | NA | <=WB |
| 104 | DRB1_0101 | KLARLVMEKTPHCF | 4 | LVMEKTPHC | 1.000 | 4O0H_PDBID | 0.289687 | 4.65 | NA | <=WB |
| 105 | DRB1_0101 | LARLVMEKTPHCFL | 3 | LVMEKTPHC | 1.000 | 4O0H_PDBID | 0.337699 | 4.05 | NA | <=WB |
| 106 | DRB1_0101 | ARLVMEKTPHCFLT | 2 | LVMEKTPHC | 1.000 | 4O0H_PDBID | 0.320367 | 4.25 | NA | <=WB |
| 123 | DRB1_0101 | AAQFAAAMGVPEIP | 3 | FAAAMGVPE | 1.000 | 4O0H_PDBID | 0.377623 | 3.63 | NA | <=WB |
| 124 | DRB1_0101 | AQFAAAMGVPEIPG | 2 | FAAAMGVPE | 1.000 | 4O0H_PDBID | 0.285966 | 4.70 | NA | <=WB |
| 180 | DRB1_0101 | NVAYATSTGGIVNK | 3 | YATSTGGIV | 1.000 | 4O0H_PDBID | 0.484222 | 2.66 | NA | <=WB |
| 188 | DRB1_0101 | GGIVNKMVGRVGDS | 3 | VNKMVGRVG | 0.980 | 4O0H_PDBID | 0.343882 | 3.98 | NA | <=WB |
| 232 | DRB1_0101 | ARLTLFHIEQGKTV | 5 | FHIEQGKTV | 1.000 | 4O0H_PDBID | 0.337233 | 4.06 | NA | <=WB |
| 236 | DRB1_0101 | LFHIEQGKTVEEAA | 1 | FHIEQGKTV | 1.000 | 4O0H_PDBID | 0.660758 | 1.55 | NA | <=WB |
| 256 | DRB1_0101 | MKSRVKGLGGLIVV | 4 | VKGLGGLIV | 1.000 | 4O0H_PDBID | 0.537931 | 2.27 | NA | <=WB |
| 257 | DRB1_0101 | KSRVKGLGGLIVVS | 3 | VKGLGGLIV | 1.000 | 4O0H_PDBID | 0.756743 | 1.09 | NA | <=WB |

| | | | | | | | | | | |
|---|---|---|---|---|---|---|---|---|---|---|
| 258 | DRB1_0101 | SRVKGLGGLIVVSK | 2 | VKGLGGLIV | 1.000 | 4O0H_PDBID | 0.621928 | 1.75 | NA | <=WB |
| 1 | DRB1_0101 | HMNPIVVVHGGGAGP | 4 | IVVVHGGGA | 1.000 | 4O0H_PDBID | 0.734173 | 1.18 | NA | <=WB |
| 2 | DRB1_0101 | MNPIVVVHGGGAGPI | 3 | IVVVHGGGA | 1.000 | 4O0H_PDBID | 0.733037 | 1.18 | NA | <=WB |
| 3 | DRB1_0101 | NPIVVVHGGGAGPIS | 2 | IVVVHGGGA | 0.987 | 4O0H_PDBID | 0.540890 | 2.25 | NA | <=WB |
| 33 | DRB1_0101 | TVGYGILREGGSAVD | 5 | ILREGGSAV | 0.627 | 4O0H_PDBID | 0.360550 | 3.81 | NA | <=WB |
| 34 | DRB1_0101 | VGYGILREGGSAVDA | 4 | ILREGGSAV | 0.987 | 4O0H_PDBID | 0.600244 | 1.87 | NA | <=WB |
| 35 | DRB1_0101 | GYGILREGGSAVDAV | 3 | ILREGGSAV | 1.000 | 4O0H_PDBID | 0.655353 | 1.58 | NA | <=WB |
| 43 | DRB1_0101 | GSAVDAVEGAVVALE | 3 | VDAVEGAVV | 0.993 | 4O0H_PDBID | 0.301235 | 4.48 | NA | <=WB |
| 59 | DRB1_0101 | DPEFNAGCGSVLNTN | 3 | FNAGCGSVL | 1.000 | 4O0H_PDBID | 0.276263 | 4.85 | NA | <=WB |
| 104 | DRB1_0101 | KLARLVMEKTPHCFL | 4 | LVMEKTPHC | 1.000 | 4O0H_PDBID | 0.308310 | 4.40 | NA | <=WB |
| 105 | DRB1_0101 | LARLVMEKTPHCFLT | 3 | LVMEKTPHC | 1.000 | 4O0H_PDBID | 0.367026 | 3.74 | NA | <=WB |
| 122 | DRB1_0101 | GAAQFAAAMGVPEIP | 4 | FAAAMGVPE | 1.000 | 4O0H_PDBID | 0.369261 | 3.71 | NA | <=WB |
| 123 | DRB1_0101 | AAQFAAAMGVPEIPG | 3 | FAAAMGVPE | 1.000 | 4O0H_PDBID | 0.528276 | 2.33 | NA | <=WB |
| 178 | DRB1_0101 | KGNVAYATSTGGIVN | 3 | VAYATSTGG | 0.547 | 4O0H_PDBID | 0.274336 | 4.87 | NA | <=WB |
| 179 | DRB1_0101 | GNVAYATSTGGIVNK | 4 | YATSTGGIV | 0.993 | 4O0H_PDBID | 0.463845 | 2.84 | NA | <=WB |
| 180 | DRB1_0101 | NVAYATSTGGIVNKM | 3 | YATSTGGIV | 1.000 | 4O0H_PDBID | 0.457679 | 2.89 | NA | <=WB |
| 187 | DRB1_0101 | TGGIVNKMVGRVGDS | 4 | VNKMVGRVG | 0.953 | 4O0H_PDBID | 0.354398 | 3.87 | NA | <=WB |
| 188 | DRB1_0101 | GGIVNKMVGRVGDSP | 3 | VNKMVGRVG | 0.993 | 4O0H_PDBID | 0.503391 | 2.50 | NA | <=WB |
| 236 | DRB1_0101 | LFHIEQGKTVEEAAD | 1 | FHIEQGKTV | 1.000 | 4O0H_PDBID | 0.484952 | 2.65 | NA | <=WB |
| 255 | DRB1_0101 | YMKSRVKGLGGLIVV | 5 | VKGLGGLIV | 1.000 | 4O0H_PDBID | 0.447356 | 2.98 | NA | <=WB |
| 256 | DRB1_0101 | MKSRVKGLGGLIVVS | 4 | VKGLGGLIV | 1.000 | 4O0H_PDBID | 0.709083 | 1.31 | NA | <=WB |
| 258 | DRB1_0101 | SRVKGLGGLIVVSKT | 2 | VKGLGGLIV | 1.000 | 4O0H_PDBID | 0.487787 | 2.63 | NA | <=WB |
| 283 | DRB1_0101 | SMPWAAAKDGKLHFG | 3 | WAAAKDGKL | 1.000 | 4O0H_PDBID | 0.395875 | 3.44 | NA | <=WB |

# Allele: DRB1_0301

| | | | | | | | | | | |
|---|---|---|---|---|---|---|---|---|---|---|
| 13 | DRB1_0301 | AGPISKDRKERV | 3 | ISKDRKERV | 1.000 | 4O0H_PDBID | 0.795437 | 0.60 | NA | <=SB |
| 14 | DRB1_0301 | GPISKDRKERVH | 2 | ISKDRKERV | 1.000 | 4O0H_PDBID | 0.888930 | 0.29 | NA | <=SB |
| 15 | DRB1_0301 | PISKDRKERVHQ | 1 | ISKDRKERV | 1.000 | 4O0H_PDBID | 0.729421 | 0.87 | NA | <=SB |
| 12 | DRB1_0301 | GAGPISKDRKERV | 4 | ISKDRKERV | 1.000 | 4O0H_PDBID | 0.853889 | 0.40 | NA | <=SB |
| 13 | DRB1_0301 | AGPISKDRKERVH | 3 | ISKDRKERV | 1.000 | 4O0H_PDBID | 0.951725 | 0.09 | NA | <=SB |
| 14 | DRB1_0301 | GPISKDRKERVHQ | 2 | ISKDRKERV | 1.000 | 4O0H_PDBID | 0.941981 | 0.11 | NA | <=SB |
| 15 | DRB1_0301 | PISKDRKERVHQG | 1 | ISKDRKERV | 1.000 | 4O0H_PDBID | 0.835588 | 0.46 | NA | <=SB |
| 11 | DRB1_0301 | GGAGPISKDRKERV | 5 | ISKDRKERV | 1.000 | 4O0H_PDBID | 0.807664 | 0.55 | NA | <=SB |
| 12 | DRB1_0301 | GAGPISKDRKERVH | 4 | ISKDRKERV | 1.000 | 4O0H_PDBID | 0.948722 | 0.10 | NA | <=SB |
| 13 | DRB1_0301 | AGPISKDRKERVHQ | 3 | ISKDRKERV | 1.000 | 4O0H_PDBID | 0.961202 | 0.05 | NA | <=SB |
| 14 | DRB1_0301 | GPISKDRKERVHQG | 2 | ISKDRKERV | 1.000 | 4O0H_PDBID | 0.944649 | 0.10 | NA | <=SB |
| 15 | DRB1_0301 | PISKDRKERVHQGM | 1 | ISKDRKERV | 1.000 | 4O0H_PDBID | 0.767790 | 0.70 | NA | <=SB |
| 170 | DRB1_0301 | VGAVALDCKGNVAY | 3 | VALDCKGNV | 1.000 | 4O0H_PDBID | 0.732741 | 0.85 | NA | <=SB |
| 10 | DRB1_0301 | GGGAGPISKDRKERV | 6 | ISKDRKERV | 1.000 | 4O0H_PDBID | 0.730762 | 0.86 | NA | <=SB |
| 11 | DRB1_0301 | GGAGPISKDRKERVH | 5 | ISKDRKERV | 1.000 | 4O0H_PDBID | 0.932647 | 0.14 | NA | <=SB |
| 12 | DRB1_0301 | GAGPISKDRKERVHQ | 4 | ISKDRKERV | 1.000 | 4O0H_PDBID | 0.954829 | 0.07 | NA | <=SB |
| 13 | DRB1_0301 | AGPISKDRKERVHQG | 3 | ISKDRKERV | 1.000 | 4O0H_PDBID | 0.960116 | 0.05 | NA | <=SB |
| 14 | DRB1_0301 | GPISKDRKERVHQGM | 2 | ISKDRKERV | 1.000 | 4O0H_PDBID | 0.921955 | 0.18 | NA | <=SB |
| 73 | DRB1_0301 | NGEVEMDASIMDGKD | 3 | VEMDASIMD | 1.000 | 4O0H_PDBID | 0.763901 | 0.72 | NA | <=SB |
| 169 | DRB1_0301 | TVGAVALDCKGNVAY | 4 | VALDCKGNV | 1.000 | 4O0H_PDBID | 0.706531 | 0.96 | NA | <=SB |
| 170 | DRB1_0301 | VGAVALDCKGNVAYA | 3 | VALDCKGNV | 1.000 | 4O0H_PDBID | 0.745860 | 0.79 | NA | <=SB |
| 14 | DRB1_0301 | GPISKDRKERV | 2 | ISKDRKERV | 1.000 | 4O0H_PDBID | 0.563047 | 1.67 | NA | <=WB |
| 15 | DRB1_0301 | PISKDRKERVH | 1 | ISKDRKERV | 1.000 | 4O0H_PDBID | 0.536466 | 1.80 | NA | <=WB |
| 171 | DRB1_0301 | GAVALDCKGNVA | 2 | VALDCKGNV | 1.000 | 4O0H_PDBID | 0.406877 | 2.75 | NA | <=WB |
| 73 | DRB1_0301 | NGEVEMDASIMDG | 3 | VEMDASIMD | 1.000 | 4O0H_PDBID | 0.416057 | 2.67 | NA | <=WB |
| 74 | DRB1_0301 | GEVEMDASIMDGK | 2 | VEMDASIMD | 1.000 | 4O0H_PDBID | 0.450544 | 2.39 | NA | <=WB |

| | | | | | | | | | | |
|---|---|---|---|---|---|---|---|---|---|---|
| 136 | DRB1_0301 | PGEKLVTERNKKR | 4 | LVTERNKKR | 0.987 | 4O0H_PDBID | 0.251198 | 4.50 | NA | <=WB |
| 137 | DRB1_0301 | GEKLVTERNKKRL | 3 | LVTERNKKR | 1.000 | 4O0H_PDBID | 0.420967 | 2.62 | NA | <=WB |
| 138 | DRB1_0301 | EKLVTERNKKRLE | 2 | LVTERNKKR | 1.000 | 4O0H_PDBID | 0.355246 | 3.20 | NA | <=WB |
| 169 | DRB1_0301 | TVGAVALDCKGNV | 4 | VALDCKGNV | 1.000 | 4O0H_PDBID | 0.307280 | 3.70 | NA | <=WB |
| 170 | DRB1_0301 | VGAVALDCKGNVA | 3 | VALDCKGNV | 1.000 | 4O0H_PDBID | 0.693143 | 1.02 | NA | <=WB |
| 171 | DRB1_0301 | GAVALDCKGNVAY | 2 | VALDCKGNV | 1.000 | 4O0H_PDBID | 0.621616 | 1.35 | NA | <=WB |
| 172 | DRB1_0301 | AVALDCKGNVAYA | 1 | VALDCKGNV | 1.000 | 4O0H_PDBID | 0.241995 | 4.66 | NA | <=WB |
| 295 | DRB1_0301 | HFGIDPDDTTITD | 3 | IDPDDTTIT | 0.967 | 4O0H_PDBID | 0.279165 | 4.07 | NA | <=WB |
| 72 | DRB1_0301 | TNGEVEMDASIMDG | 4 | VEMDASIMD | 1.000 | 4O0H_PDBID | 0.395541 | 2.84 | NA | <=WB |
| 73 | DRB1_0301 | NGEVEMDASIMDGK | 3 | VEMDASIMD | 1.000 | 4O0H_PDBID | 0.626170 | 1.33 | NA | <=WB |
| 74 | DRB1_0301 | GEVEMDASIMDGKD | 2 | VEMDASIMD | 0.993 | 4O0H_PDBID | 0.528498 | 1.85 | NA | <=WB |
| 136 | DRB1_0301 | PGEKLVTERNKKRL | 4 | LVTERNKKR | 1.000 | 4O0H_PDBID | 0.395662 | 2.84 | NA | <=WB |
| 137 | DRB1_0301 | GEKLVTERNKKRLE | 3 | LVTERNKKR | 1.000 | 4O0H_PDBID | 0.483708 | 2.16 | NA | <=WB |
| 138 | DRB1_0301 | EKLVTERNKKRLEK | 2 | LVTERNKKR | 0.993 | 4O0H_PDBID | 0.412246 | 2.70 | NA | <=WB |
| 168 | DRB1_0301 | GTVGAVALDCKGNV | 5 | VALDCKGNV | 1.000 | 4O0H_PDBID | 0.229031 | 4.88 | NA | <=WB |
| 169 | DRB1_0301 | TVGAVALDCKGNVA | 4 | VALDCKGNV | 1.000 | 4O0H_PDBID | 0.657289 | 1.19 | NA | <=WB |
| 171 | DRB1_0301 | GAVALDCKGNVAYA | 2 | VALDCKGNV | 1.000 | 4O0H_PDBID | 0.634596 | 1.29 | NA | <=WB |
| 285 | DRB1_0301 | PWAAAKDGKLHFGI | 3 | AAKDGKLHF | 1.000 | 4O0H_PDBID | 0.238018 | 4.72 | NA | <=WB |
| 294 | DRB1_0301 | LHFGIDPDDTTITD | 4 | IDPDDTTIT | 0.893 | 4O0H_PDBID | 0.272281 | 4.18 | NA | <=WB |
| 295 | DRB1_0301 | HFGIDPDDTTITDL | 3 | IDPDDTTIT | 0.987 | 4O0H_PDBID | 0.296260 | 3.84 | NA | <=WB |
| 296 | DRB1_0301 | FGIDPDDTTITDLP | 2 | IDPDDTTIT | 1.000 | 4O0H_PDBID | 0.228716 | 4.89 | NA | <=WB |
| 15 | DRB1_0301 | PISKDRKERVHQGMV | 1 | ISKDRKERV | 1.000 | 4O0H_PDBID | 0.678569 | 1.08 | NA | <=WB |
| 51 | DRB1_0301 | GAVVALEDDPEFNAG | 5 | LEDDPEFNA | 0.620 | 4O0H_PDBID | 0.242519 | 4.65 | NA | <=WB |
| 71 | DRB1_0301 | NTNGEVEMDASIMDG | 5 | VEMDASIMD | 1.000 | 4O0H_PDBID | 0.415130 | 2.68 | NA | <=WB |
| 72 | DRB1_0301 | TNGEVEMDASIMDGK | 4 | VEMDASIMD | 1.000 | 4O0H_PDBID | 0.687719 | 1.04 | NA | <=WB |
| 74 | DRB1_0301 | GEVEMDASIMDGKDL | 2 | VEMDASIMD | 1.000 | 4O0H_PDBID | 0.533156 | 1.82 | NA | <=WB |

| | | | | | | | | | | |
|---|---|---|---|---|---|---|---|---|---|---|
| 114 | DRB1_0301 | PHCFLTDQGAAQFAA | 3 | FLTDQGAAQ | 1.000 | 4O0H_PDBID | 0.244545 | 4.61 | NA | <=WB |
| 135 | DRB1_0301 | IPGEKLVTERNKKRL | 5 | LVTERNKKR | 0.993 | 4O0H_PDBID | 0.392786 | 2.87 | NA | <=WB |
| 136 | DRB1_0301 | PGEKLVTERNKKRLE | 4 | LVTERNKKR | 1.000 | 4O0H_PDBID | 0.513817 | 1.94 | NA | <=WB |
| 137 | DRB1_0301 | GEKLVTERNKKRLEK | 3 | LVTERNKKR | 1.000 | 4O0H_PDBID | 0.591510 | 1.51 | NA | <=WB |
| 138 | DRB1_0301 | EKLVTERNKKRLEKE | 2 | LVTERNKKR | 0.993 | 4O0H_PDBID | 0.384751 | 2.94 | NA | <=WB |
| 168 | DRB1_0301 | GTVGAVALDCKGNVA | 5 | VALDCKGNV | 1.000 | 4O0H_PDBID | 0.593264 | 1.50 | NA | <=WB |
| 171 | DRB1_0301 | GAVALDCKGNVAYAT | 2 | VALDCKGNV | 1.000 | 4O0H_PDBID | 0.530504 | 1.84 | NA | <=WB |
| 284 | DRB1_0301 | MPWAAAKDGKLHFGI | 4 | AAKDGKLHF | 1.000 | 4O0H_PDBID | 0.240768 | 4.68 | NA | <=WB |
| 285 | DRB1_0301 | PWAAAKDGKLHFGID | 3 | AAKDGKLHF | 1.000 | 4O0H_PDBID | 0.330045 | 3.43 | NA | <=WB |
| 293 | DRB1_0301 | KLHFGIDPDDTTITD | 5 | IDPDDTTIT | 0.747 | 4O0H_PDBID | 0.307529 | 3.70 | NA | <=WB |
| 294 | DRB1_0301 | LHFGIDPDDTTITDL | 4 | IDPDDTTIT | 0.967 | 4O0H_PDBID | 0.329248 | 3.44 | NA | <=WB |
| 295 | DRB1_0301 | HFGIDPDDTTITDLP | 3 | IDPDDTTIT | 0.987 | 4O0H_PDBID | 0.432742 | 2.52 | NA | <=WB |

# Allele: DRB1_0401

| | | | | | | | | | | |
|---|---|---|---|---|---|---|---|---|---|---|
| 106 | DRB1_0401 | ARLVMEKTPHCF | 2 | LVMEKTPHC | 1.000 | 4O0H_PDBID | 0.369495 | 3.42 | NA | <=WB |
| 73 | DRB1_0401 | NGEVEMDASIMDG | 3 | VEMDASIMD | 1.000 | 4O0H_PDBID | 0.295383 | 4.62 | NA | <=WB |
| 74 | DRB1_0401 | GEVEMDASIMDGK | 2 | VEMDASIMD | 1.000 | 4O0H_PDBID | 0.318607 | 4.20 | NA | <=WB |
| 104 | DRB1_0401 | KLARLVMEKTPHC | 4 | LVMEKTPHC | 1.000 | 4O0H_PDBID | 0.278960 | 4.94 | NA | <=WB |
| 105 | DRB1_0401 | LARLVMEKTPHCF | 3 | LVMEKTPHC | 1.000 | 4O0H_PDBID | 0.583614 | 1.47 | NA | <=WB |
| 106 | DRB1_0401 | ARLVMEKTPHCFL | 2 | LVMEKTPHC | 1.000 | 4O0H_PDBID | 0.469519 | 2.36 | NA | <=WB |
| 114 | DRB1_0401 | PHCFLTDQGAAQF | 3 | FLTDQGAAQ | 1.000 | 4O0H_PDBID | 0.531471 | 1.86 | NA | <=WB |
| 115 | DRB1_0401 | HCFLTDQGAAQFA | 2 | FLTDQGAAQ | 1.000 | 4O0H_PDBID | 0.372406 | 3.38 | NA | <=WB |
| 178 | DRB1_0401 | KGNVAYATSTGGI | 3 | VAYATSTGG | 0.987 | 4O0H_PDBID | 0.279827 | 4.92 | NA | <=WB |
| 293 | DRB1_0401 | KLHFGIDPDDTTI | 3 | FGIDPDDTT | 0.973 | 4O0H_PDBID | 0.293047 | 4.66 | NA | <=WB |
| 73 | DRB1_0401 | NGEVEMDASIMDGK | 3 | VEMDASIMD | 1.000 | 4O0H_PDBID | 0.527907 | 1.89 | NA | <=WB |

| | | | | | | | | | | |
|---|---|---|---|---|---|---|---|---|---|---|
| 74 | DRB1_0401 | GEVEMDASIMDGKD | 2 | VEMDASIMD | 1.000 | 4O0H_PDBID | 0.404888 | 2.98 | NA | <=WB |
| 104 | DRB1_0401 | KLARLVMEKTPHCF | 4 | LVMEKTPHC | 1.000 | 4O0H_PDBID | 0.539727 | 1.80 | NA | <=WB |
| 105 | DRB1_0401 | LARLVMEKTPHCFL | 3 | LVMEKTPHC | 1.000 | 4O0H_PDBID | 0.513852 | 1.99 | NA | <=WB |
| 106 | DRB1_0401 | ARLVMEKTPHCFLT | 2 | LVMEKTPHC | 1.000 | 4O0H_PDBID | 0.456011 | 2.47 | NA | <=WB |
| 113 | DRB1_0401 | TPHCFLTDQGAAQF | 4 | FLTDQGAAQ | 1.000 | 4O0H_PDBID | 0.471377 | 2.34 | NA | <=WB |
| 114 | DRB1_0401 | PHCFLTDQGAAQFA | 3 | FLTDQGAAQ | 1.000 | 4O0H_PDBID | 0.625057 | 1.21 | NA | <=WB |
| 115 | DRB1_0401 | HCFLTDQGAAQFAA | 2 | FLTDQGAAQ | 1.000 | 4O0H_PDBID | 0.386352 | 3.20 | NA | <=WB |
| 178 | DRB1_0401 | KGNVAYATSTGGIV | 3 | VAYATSTGG | 1.000 | 4O0H_PDBID | 0.332082 | 3.98 | NA | <=WB |
| 293 | DRB1_0401 | KLHFGIDPDDTTIT | 3 | FGIDPDDTT | 0.987 | 4O0H_PDBID | 0.378625 | 3.30 | NA | <=WB |
| 294 | DRB1_0401 | LHFGIDPDDTTITD | 2 | FGIDPDDTT | 0.533 | 4O0H_PDBID | 0.302679 | 4.48 | NA | <=WB |
| 50 | DRB1_0401 | EGAVVALEDDPEFNA | 3 | VVALEDDPE | 0.867 | 4O0H_PDBID | 0.304441 | 4.45 | NA | <=WB |
| 72 | DRB1_0401 | TNGEVEMDASIMDGK | 4 | VEMDASIMD | 1.000 | 4O0H_PDBID | 0.513649 | 1.99 | NA | <=WB |
| 73 | DRB1_0401 | NGEVEMDASIMDGKD | 3 | VEMDASIMD | 1.000 | 4O0H_PDBID | 0.636779 | 1.14 | NA | <=WB |
| 74 | DRB1_0401 | GEVEMDASIMDGKDL | 2 | VEMDASIMD | 1.000 | 4O0H_PDBID | 0.330792 | 4.00 | NA | <=WB |
| 103 | DRB1_0401 | IKLARLVMEKTPHCF | 5 | LVMEKTPHC | 1.000 | 4O0H_PDBID | 0.427185 | 2.75 | NA | <=WB |
| 104 | DRB1_0401 | KLARLVMEKTPHCFL | 4 | LVMEKTPHC | 1.000 | 4O0H_PDBID | 0.446446 | 2.56 | NA | <=WB |
| 105 | DRB1_0401 | LARLVMEKTPHCFLT | 3 | LVMEKTPHC | 1.000 | 4O0H_PDBID | 0.474297 | 2.32 | NA | <=WB |
| 106 | DRB1_0401 | ARLVMEKTPHCFLTD | 2 | LVMEKTPHC | 1.000 | 4O0H_PDBID | 0.325840 | 4.08 | NA | <=WB |
| 112 | DRB1_0401 | KTPHCFLTDQGAAQF | 5 | FLTDQGAAQ | 1.000 | 4O0H_PDBID | 0.355881 | 3.61 | NA | <=WB |
| 113 | DRB1_0401 | TPHCFLTDQGAAQFA | 4 | FLTDQGAAQ | 1.000 | 4O0H_PDBID | 0.557882 | 1.67 | NA | <=WB |
| 114 | DRB1_0401 | PHCFLTDQGAAQFAA | 3 | FLTDQGAAQ | 1.000 | 4O0H_PDBID | 0.626289 | 1.20 | NA | <=WB |
| 123 | DRB1_0401 | AAQFAAAMGVPEIPG | 3 | FAAAMGVPE | 0.993 | 4O0H_PDBID | 0.285592 | 4.81 | NA | <=WB |
| 177 | DRB1_0401 | CKGNVAYATSTGGIV | 4 | VAYATSTGG | 1.000 | 4O0H_PDBID | 0.329979 | 4.01 | NA | <=WB |
| 178 | DRB1_0401 | KGNVAYATSTGGIVN | 3 | VAYATSTGG | 1.000 | 4O0H_PDBID | 0.435767 | 2.67 | NA | <=WB |
| 283 | DRB1_0401 | SMPWAAAKDGKLHFG | 3 | WAAAKDGKL | 1.000 | 4O0H_PDBID | 0.285509 | 4.81 | NA | <=WB |
| 292 | DRB1_0401 | GKLHFGIDPDDTTIT | 4 | FGIDPDDTT | 0.967 | 4O0H_PDBID | 0.353531 | 3.65 | NA | <=WB |

| | | | | | | | | | | |
|---|---|---|---|---|---|---|---|---|---|---|
| 293 | DRB1_0401 | KLHFGIDPDDTTITD | 3 | FGIDPDDTT | 0.813 | 4O0H_PDBID | 0.511403 | 2.01 | NA | <=WB |
| 295 | DRB1_0401 | HFGIDPDDTTITDLP | 3 | IDPDDTTIT | 0.953 | 4O0H_PDBID | 0.299212 | 4.54 | NA | <=WB |

# Allele: DRB1_0701

| | | | | | | | | | | |
|---|---|---|---|---|---|---|---|---|---|---|
| 180 | DRB1_0701 | NVAYATSTGGIVN | 3 | YATSTGGIV | 1.000 | 4O0H_PDBID | 0.692139 | 0.75 | NA | <=SB |
| 181 | DRB1_0701 | VAYATSTGGIVNK | 2 | YATSTGGIV | 1.000 | 4O0H_PDBID | 0.632000 | 0.99 | NA | <=SB |
| 179 | DRB1_0701 | GNVAYATSTGGIVN | 4 | YATSTGGIV | 0.993 | 4O0H_PDBID | 0.669566 | 0.84 | NA | <=SB |
| 180 | DRB1_0701 | NVAYATSTGGIVNK | 3 | YATSTGGIV | 1.000 | 4O0H_PDBID | 0.810703 | 0.37 | NA | <=SB |
| 178 | DRB1_0701 | KGNVAYATSTGGIVN | 5 | YATSTGGIV | 0.927 | 4O0H_PDBID | 0.636043 | 0.97 | NA | <=SB |
| 179 | DRB1_0701 | GNVAYATSTGGIVNK | 4 | YATSTGGIV | 0.973 | 4O0H_PDBID | 0.804572 | 0.39 | NA | <=SB |
| 180 | DRB1_0701 | NVAYATSTGGIVNKM | 3 | YATSTGGIV | 1.000 | 4O0H_PDBID | 0.769086 | 0.49 | NA | <=SB |
| 180 | DRB1_0701 | NVAYATSTGGIV | 3 | YATSTGGIV | 0.973 | 4O0H_PDBID | 0.267935 | 4.42 | NA | <=WB |
| 181 | DRB1_0701 | VAYATSTGGIVN | 2 | YATSTGGIV | 1.000 | 4O0H_PDBID | 0.318897 | 3.59 | NA | <=WB |
| 123 | DRB1_0701 | AAQFAAAMGVPEI | 3 | FAAAMGVPE | 0.980 | 4O0H_PDBID | 0.253273 | 4.70 | NA | <=WB |
| 164 | DRB1_0701 | QKNLGTVGAVALD | 3 | LGTVGAVAL | 1.000 | 4O0H_PDBID | 0.323134 | 3.53 | NA | <=WB |
| 179 | DRB1_0701 | GNVAYATSTGGIV | 4 | YATSTGGIV | 0.900 | 4O0H_PDBID | 0.387379 | 2.73 | NA | <=WB |
| 234 | DRB1_0701 | LTLFHIEQGKTVE | 3 | FHIEQGKTV | 1.000 | 4O0H_PDBID | 0.331161 | 3.42 | NA | <=WB |
| 235 | DRB1_0701 | TLFHIEQGKTVEE | 2 | FHIEQGKTV | 0.993 | 4O0H_PDBID | 0.336301 | 3.35 | NA | <=WB |
| 252 | DRB1_0701 | SLGYMKSRVKGLG | 3 | YMKSRVKGL | 1.000 | 4O0H_PDBID | 0.474788 | 1.96 | NA | <=WB |
| 253 | DRB1_0701 | LGYMKSRVKGLGG | 2 | YMKSRVKGL | 1.000 | 4O0H_PDBID | 0.355080 | 3.09 | NA | <=WB |
| 283 | DRB1_0701 | SMPWAAAKDGKLH | 3 | WAAAKDGKL | 1.000 | 4O0H_PDBID | 0.349419 | 3.17 | NA | <=WB |
| 122 | DRB1_0701 | GAAQFAAAMGVPEI | 4 | FAAAMGVPE | 0.907 | 4O0H_PDBID | 0.239993 | 4.97 | NA | <=WB |
| 123 | DRB1_0701 | AAQFAAAMGVPEIP | 3 | FAAAMGVPE | 0.987 | 4O0H_PDBID | 0.376377 | 2.85 | NA | <=WB |
| 124 | DRB1_0701 | AQFAAAMGVPEIPG | 2 | FAAAMGVPE | 0.987 | 4O0H_PDBID | 0.250850 | 4.75 | NA | <=WB |
| 163 | DRB1_0701 | CQKNLGTVGAVALD | 4 | LGTVGAVAL | 1.000 | 4O0H_PDBID | 0.297312 | 3.92 | NA | <=WB |

| | | | | | | | | | | |
|---|---|---|---|---|---|---|---|---|---|---|
| 178 | DRB1_0701 | KGNVAYATSTGGIV | 5 | YATSTGGIV | 0.760 | 4O0H_PDBID | 0.339423 | 3.31 | NA | <=WB |
| 181 | DRB1_0701 | VAYATSTGGIVNKM | 2 | YATSTGGIV | 1.000 | 4O0H_PDBID | 0.555918 | 1.42 | NA | <=WB |
| 233 | DRB1_0701 | RLTLFHIEQGKTVE | 4 | FHIEQGKTV | 1.000 | 4O0H_PDBID | 0.314186 | 3.67 | NA | <=WB |
| 234 | DRB1_0701 | LTLFHIEQGKTVEE | 3 | FHIEQGKTV | 0.993 | 4O0H_PDBID | 0.403341 | 2.56 | NA | <=WB |
| 235 | DRB1_0701 | TLFHIEQGKTVEEA | 2 | FHIEQGKTV | 0.987 | 4O0H_PDBID | 0.370295 | 2.91 | NA | <=WB |
| 251 | DRB1_0701 | LSLGYMKSRVKGLG | 4 | YMKSRVKGL | 1.000 | 4O0H_PDBID | 0.426749 | 2.35 | NA | <=WB |
| 252 | DRB1_0701 | SLGYMKSRVKGLGG | 3 | YMKSRVKGL | 1.000 | 4O0H_PDBID | 0.522432 | 1.63 | NA | <=WB |
| 253 | DRB1_0701 | LGYMKSRVKGLGGL | 2 | YMKSRVKGL | 1.000 | 4O0H_PDBID | 0.258149 | 4.60 | NA | <=WB |
| 282 | DRB1_0701 | TSMPWAAAKDGKLH | 4 | WAAAKDGKL | 1.000 | 4O0H_PDBID | 0.339025 | 3.31 | NA | <=WB |
| 283 | DRB1_0701 | SMPWAAAKDGKLHF | 3 | WAAAKDGKL | 1.000 | 4O0H_PDBID | 0.350708 | 3.15 | NA | <=WB |
| 1 | DRB1_0701 | HMNPIVVVHGGGAGP | 4 | IVVVHGGGA | 0.993 | 4O0H_PDBID | 0.263757 | 4.49 | NA | <=WB |
| 2 | DRB1_0701 | MNPIVVVHGGGAGPI | 3 | IVVVHGGGA | 0.953 | 4O0H_PDBID | 0.250739 | 4.75 | NA | <=WB |
| 121 | DRB1_0701 | QGAAQFAAAMGVPEI | 5 | FAAAMGVPE | 0.693 | 4O0H_PDBID | 0.248672 | 4.79 | NA | <=WB |
| 122 | DRB1_0701 | GAAQFAAAMGVPEIP | 4 | FAAAMGVPE | 0.973 | 4O0H_PDBID | 0.385080 | 2.76 | NA | <=WB |
| 123 | DRB1_0701 | AAQFAAAMGVPEIPG | 3 | FAAAMGVPE | 0.987 | 4O0H_PDBID | 0.449923 | 2.16 | NA | <=WB |
| 162 | DRB1_0701 | DCQKNLGTVGAVALD | 5 | LGTVGAVAL | 0.993 | 4O0H_PDBID | 0.283205 | 4.16 | NA | <=WB |
| 164 | DRB1_0701 | QKNLGTVGAVALDCK | 3 | LGTVGAVAL | 0.993 | 4O0H_PDBID | 0.286821 | 4.09 | NA | <=WB |
| 177 | DRB1_0701 | CKGNVAYATSTGGIV | 6 | YATSTGGIV | 0.700 | 4O0H_PDBID | 0.293971 | 3.98 | NA | <=WB |
| 181 | DRB1_0701 | VAYATSTGGIVNKMV | 2 | YATSTGGIV | 0.993 | 4O0H_PDBID | 0.449922 | 2.16 | NA | <=WB |
| 232 | DRB1_0701 | ARLTLFHIEQGKTVE | 5 | FHIEQGKTV | 0.987 | 4O0H_PDBID | 0.259722 | 4.57 | NA | <=WB |
| 233 | DRB1_0701 | RLTLFHIEQGKTVEE | 4 | FHIEQGKTV | 0.993 | 4O0H_PDBID | 0.381479 | 2.79 | NA | <=WB |
| 234 | DRB1_0701 | LTLFHIEQGKTVEEA | 3 | FHIEQGKTV | 1.000 | 4O0H_PDBID | 0.420689 | 2.40 | NA | <=WB |
| 235 | DRB1_0701 | TLFHIEQGKTVEEAA | 2 | FHIEQGKTV | 0.967 | 4O0H_PDBID | 0.281718 | 4.18 | NA | <=WB |
| 250 | DRB1_0701 | DLSLGYMKSRVKGLG | 5 | YMKSRVKGL | 0.987 | 4O0H_PDBID | 0.362089 | 3.00 | NA | <=WB |
| 251 | DRB1_0701 | LSLGYMKSRVKGLGG | 4 | YMKSRVKGL | 1.000 | 4O0H_PDBID | 0.503141 | 1.76 | NA | <=WB |
| 252 | DRB1_0701 | SLGYMKSRVKGLGGL | 3 | YMKSRVKGL | 1.000 | 4O0H_PDBID | 0.433228 | 2.30 | NA | <=WB |

| | | | | | | | | | | |
|---|---|---|---|---|---|---|---|---|---|---|
| 281 | DRB1_0701 | STSMPWAAAKDGKLH | 5 | WAAAKDGKL | 1.000 | 4O0H_PDBID | 0.341089 | 3.28 | NA | <=WB |
| 282 | DRB1_0701 | TSMPWAAAKDGKLHF | 4 | WAAAKDGKL | 1.000 | 4O0H_PDBID | 0.385034 | 2.76 | NA | <=WB |
| 283 | DRB1_0701 | SMPWAAAKDGKLHFG | 3 | WAAAKDGKL | 1.000 | 4O0H_PDBID | 0.434615 | 2.29 | NA | <=WB |

# Allele: DRB1_0801

| | | | | | | | | | | |
|---|---|---|---|---|---|---|---|---|---|---|
| 263 | DRB1_0801 | LGGLIVVSKTGDW | 3 | LIVVSKTGD | 1.000 | 4O0H_PDBID | 0.847285 | 0.38 | NA | <=SB |
| 264 | DRB1_0801 | GGLIVVSKTGDWV | 2 | LIVVSKTGD | 1.000 | 4O0H_PDBID | 0.835851 | 0.43 | NA | <=SB |
| 262 | DRB1_0801 | GLGGLIVVSKTGDW | 4 | LIVVSKTGD | 1.000 | 4O0H_PDBID | 0.821740 | 0.50 | NA | <=SB |
| 263 | DRB1_0801 | LGGLIVVSKTGDWV | 3 | LIVVSKTGD | 1.000 | 4O0H_PDBID | 0.840129 | 0.41 | NA | <=SB |
| 264 | DRB1_0801 | GGLIVVSKTGDWVA | 2 | LIVVSKTGD | 1.000 | 4O0H_PDBID | 0.853021 | 0.36 | NA | <=SB |
| 261 | DRB1_0801 | KGLGGLIVVSKTGDW | 5 | LIVVSKTGD | 1.000 | 4O0H_PDBID | 0.767702 | 0.80 | NA | <=SB |
| 262 | DRB1_0801 | GLGGLIVVSKTGDWV | 4 | LIVVSKTGD | 1.000 | 4O0H_PDBID | 0.819479 | 0.51 | NA | <=SB |
| 263 | DRB1_0801 | LGGLIVVSKTGDWVA | 3 | LIVVSKTGD | 1.000 | 4O0H_PDBID | 0.857689 | 0.34 | NA | <=SB |
| 264 | DRB1_0801 | GGLIVVSKTGDWVAK | 2 | LIVVSKTGD | 0.993 | 4O0H_PDBID | 0.767018 | 0.81 | NA | <=SB |
| 263 | DRB1_0801 | LGGLIVVSKTGD | 3 | LIVVSKTGD | 1.000 | 4O0H_PDBID | 0.526083 | 3.17 | NA | <=WB |
| 264 | DRB1_0801 | GGLIVVSKTGDW | 2 | LIVVSKTGD | 1.000 | 4O0H_PDBID | 0.717812 | 1.16 | NA | <=WB |
| 262 | DRB1_0801 | GLGGLIVVSKTGD | 4 | LIVVSKTGD | 0.987 | 4O0H_PDBID | 0.656790 | 1.66 | NA | <=WB |
| 265 | DRB1_0801 | GLIVVSKTGDWVA | 1 | LIVVSKTGD | 0.993 | 4O0H_PDBID | 0.518842 | 3.28 | NA | <=WB |
| 261 | DRB1_0801 | KGLGGLIVVSKTGD | 5 | LIVVSKTGD | 0.980 | 4O0H_PDBID | 0.557018 | 2.75 | NA | <=WB |
| 272 | DRB1_0801 | TGDWVAKWTSTSMP | 3 | WVAKWTSTS | 0.953 | 4O0H_PDBID | 0.482560 | 3.85 | NA | <=WB |
| 260 | DRB1_0801 | VKGLGGLIVVSKTGD | 6 | LIVVSKTGD | 0.967 | 4O0H_PDBID | 0.436428 | 4.73 | NA | <=WB |
| 271 | DRB1_0801 | KTGDWVAKWTSTSMP | 4 | WVAKWTSTS | 0.960 | 4O0H_PDBID | 0.459674 | 4.26 | NA | <=WB |

# Allele: DRB1_1101

| | | | | | | | | | | |
|---|---|---|---|---|---|---|---|---|---|---|
| 2 | DRB1_1101 | MNPIVVVHGGGAGP | 3 | IVVVHGGGA | 0.980 | 4O0H_PDBID | 0.370587 | 4.61 | NA | <=WB |
| 146 | DRB1_1101 | KKRLEKEKHEKGAQ | 3 | LEKEKHEKG | 1.000 | 4O0H_PDBID | 0.412402 | 4.08 | NA | <=WB |
| 263 | DRB1_1101 | LGGLIVVSKTGDWV | 3 | LIVVSKTGD | 1.000 | 4O0H_PDBID | 0.395112 | 4.29 | NA | <=WB |
| 264 | DRB1_1101 | GGLIVVSKTGDWVA | 2 | LIVVSKTGD | 0.993 | 4O0H_PDBID | 0.416927 | 4.02 | NA | <=WB |
| 1 | DRB1_1101 | HMNPIVVVHGGGAGP | 4 | IVVVHGGGA | 0.960 | 4O0H_PDBID | 0.390631 | 4.34 | NA | <=WB |
| 2 | DRB1_1101 | MNPIVVVHGGGAGPI | 3 | IVVVHGGGA | 0.973 | 4O0H_PDBID | 0.360103 | 4.78 | NA | <=WB |
| 33 | DRB1_1101 | TVGYGILREGGSAVD | 3 | YGILREGGS | 1.000 | 4O0H_PDBID | 0.421324 | 3.97 | NA | <=WB |
| 145 | DRB1_1101 | NKKRLEKEKHEKGAQ | 4 | LEKEKHEKG | 1.000 | 4O0H_PDBID | 0.394799 | 4.29 | NA | <=WB |
| 146 | DRB1_1101 | KKRLEKEKHEKGAQK | 3 | LEKEKHEKG | 1.000 | 4O0H_PDBID | 0.512616 | 2.94 | NA | <=WB |
| 262 | DRB1_1101 | GLGGLIVVSKTGDWV | 4 | LIVVSKTGD | 1.000 | 4O0H_PDBID | 0.383862 | 4.42 | NA | <=WB |
| 263 | DRB1_1101 | LGGLIVVSKTGDWVA | 3 | LIVVSKTGD | 1.000 | 4O0H_PDBID | 0.505084 | 3.01 | NA | <=WB |

# Allele: DRB1_1301

| | | | | | | | | | | |
|---|---|---|---|---|---|---|---|---|---|---|
| 137 | DRB1_1301 | GEKLVTERNKKRLE | 3 | LVTERNKKR | 0.667 | 4O0H_PDBID | 0.775160 | 0.78 | NA | <=SB |
| 138 | DRB1_1301 | EKLVTERNKKRLEK | 3 | VTERNKKRL | 0.573 | 4O0H_PDBID | 0.804879 | 0.58 | NA | <=SB |
| 136 | DRB1_1301 | PGEKLVTERNKKRLE | 4 | LVTERNKKR | 0.680 | 4O0H_PDBID | 0.751393 | 0.92 | NA | <=SB |
| 137 | DRB1_1301 | GEKLVTERNKKRLEK | 3 | LVTERNKKR | 0.593 | 4O0H_PDBID | 0.839923 | 0.39 | NA | <=SB |
| 138 | DRB1_1301 | EKLVTERNKKRLEKE | 3 | VTERNKKRL | 0.740 | 4O0H_PDBID | 0.801169 | 0.61 | NA | <=SB |
| 13 | DRB1_1301 | AGPISKDRKERVH | 3 | ISKDRKERV | 0.967 | 4O0H_PDBID | 0.523783 | 3.30 | NA | <=WB |
| 14 | DRB1_1301 | GPISKDRKERVHQ | 2 | ISKDRKERV | 0.993 | 4O0H_PDBID | 0.500647 | 3.66 | NA | <=WB |
| 137 | DRB1_1301 | GEKLVTERNKKRL | 3 | LVTERNKKR | 0.840 | 4O0H_PDBID | 0.628310 | 2.03 | NA | <=WB |
| 138 | DRB1_1301 | EKLVTERNKKRLE | 3 | VTERNKKRL | 0.520 | 4O0H_PDBID | 0.700606 | 1.31 | NA | <=WB |

| | | | | | | | | | | |
|---|---|---|---|---|---|---|---|---|---|---|
| 139 | DRB1_1301 | KLVTERNKKRLEK | 2 | VTERNKKRL | 0.713 | 4O0H_PDBID | 0.617134 | 2.15 | NA | <=WB |
| 2 | DRB1_1301 | MNPIVVVHGGGAGP | 3 | IVVVHGGGA | 0.640 | 4O0H_PDBID | 0.436216 | 4.81 | NA | <=WB |
| 12 | DRB1_1301 | GAGPISKDRKERVH | 4 | ISKDRKERV | 0.920 | 4O0H_PDBID | 0.534821 | 3.14 | NA | <=WB |
| 13 | DRB1_1301 | AGPISKDRKERVHQ | 3 | ISKDRKERV | 0.987 | 4O0H_PDBID | 0.628198 | 2.03 | NA | <=WB |
| 14 | DRB1_1301 | GPISKDRKERVHQG | 2 | ISKDRKERV | 1.000 | 4O0H_PDBID | 0.569367 | 2.69 | NA | <=WB |
| 136 | DRB1_1301 | PGEKLVTERNKKRL | 4 | LVTERNKKR | 0.860 | 4O0H_PDBID | 0.610364 | 2.22 | NA | <=WB |
| 139 | DRB1_1301 | KLVTERNKKRLEKE | 2 | VTERNKKRL | 0.820 | 4O0H_PDBID | 0.640646 | 1.90 | NA | <=WB |
| 187 | DRB1_1301 | TGGIVNKMVGRVGD | 3 | IVNKMVGRV | 0.967 | 4O0H_PDBID | 0.521849 | 3.33 | NA | <=WB |
| 188 | DRB1_1301 | GGIVNKMVGRVGDS | 2 | IVNKMVGRV | 0.887 | 4O0H_PDBID | 0.431720 | 4.90 | NA | <=WB |
| 1 | DRB1_1301 | HMNPIVVVHGGGAGP | 4 | IVVVHGGGA | 0.667 | 4O0H_PDBID | 0.469446 | 4.20 | NA | <=WB |
| 2 | DRB1_1301 | MNPIVVVHGGGAGPI | 3 | IVVVHGGGA | 0.567 | 4O0H_PDBID | 0.501903 | 3.64 | NA | <=WB |
| 3 | DRB1_1301 | NPIVVVHGGGAGPIS | 3 | VVVHGGGAG | 0.633 | 4O0H_PDBID | 0.469823 | 4.19 | NA | <=WB |
| 11 | DRB1_1301 | GGAGPISKDRKERVH | 5 | ISKDRKERV | 0.873 | 4O0H_PDBID | 0.495831 | 3.74 | NA | <=WB |
| 12 | DRB1_1301 | GAGPISKDRKERVHQ | 4 | ISKDRKERV | 0.927 | 4O0H_PDBID | 0.629941 | 2.01 | NA | <=WB |
| 13 | DRB1_1301 | AGPISKDRKERVHQG | 3 | ISKDRKERV | 0.980 | 4O0H_PDBID | 0.676421 | 1.52 | NA | <=WB |
| 14 | DRB1_1301 | GPISKDRKERVHQGM | 2 | ISKDRKERV | 0.993 | 4O0H_PDBID | 0.508031 | 3.54 | NA | <=WB |
| 135 | DRB1_1301 | IPGEKLVTERNKKRL | 5 | LVTERNKKR | 0.867 | 4O0H_PDBID | 0.545423 | 2.98 | NA | <=WB |
| 139 | DRB1_1301 | KLVTERNKKRLEKEK | 2 | VTERNKKRL | 0.813 | 4O0H_PDBID | 0.546870 | 2.97 | NA | <=WB |
| 186 | DRB1_1301 | STGGIVNKMVGRVGD | 4 | IVNKMVGRV | 0.960 | 4O0H_PDBID | 0.528945 | 3.22 | NA | <=WB |
| 187 | DRB1_1301 | TGGIVNKMVGRVGDS | 3 | IVNKMVGRV | 0.960 | 4O0H_PDBID | 0.590800 | 2.44 | NA | <=WB |

# Allele: DRB1_1501

| | | | | | | | | | | |
|---|---|---|---|---|---|---|---|---|---|---|
| 3 | DRB1_1501 | NPIVVVHGGGAG | 2 | IVVVHGGGA | 0.993 | 4O0H_PDBID | 0.261895 | 3.81 | NA | <=WB |
| 2 | DRB1_1501 | MNPIVVVHGGGAG | 3 | IVVVHGGGA | 1.000 | 4O0H_PDBID | 0.479346 | 1.80 | NA | <=WB |

| | | | | | | | | | | |
|---|---|---|---|---|---|---|---|---|---|---|
| 3 | DRB1_1501 | NPIVVVHGGGAGP | 2 | IVVVHGGGA | 0.960 | 4O0H_PDBID | 0.535023 | 1.50 | NA | <=WB |
| 257 | DRB1_1501 | KSRVKGLGGLIVV | 3 | VKGLGGLIV | 1.000 | 4O0H_PDBID | 0.210067 | 4.77 | NA | <=WB |
| 273 | DRB1_1501 | GDWVAKWTSTSMP | 3 | VAKWTSTSM | 0.993 | 4O0H_PDBID | 0.271760 | 3.67 | NA | <=WB |
| 1 | DRB1_1501 | HMNPIVVVHGGGAG | 4 | IVVVHGGGA | 0.993 | 4O0H_PDBID | 0.454273 | 1.95 | NA | <=WB |
| 2 | DRB1_1501 | MNPIVVVHGGGAGP | 3 | IVVVHGGGA | 0.987 | 4O0H_PDBID | 0.623864 | 1.10 | NA | <=WB |
| 3 | DRB1_1501 | NPIVVVHGGGAGPI | 2 | IVVVHGGGA | 0.907 | 4O0H_PDBID | 0.520709 | 1.58 | NA | <=WB |
| 257 | DRB1_1501 | KSRVKGLGGLIVVS | 3 | VKGLGGLIV | 1.000 | 4O0H_PDBID | 0.317373 | 3.11 | NA | <=WB |
| 258 | DRB1_1501 | SRVKGLGGLIVVSK | 2 | VKGLGGLIV | 1.000 | 4O0H_PDBID | 0.200532 | 4.97 | NA | <=WB |
| 272 | DRB1_1501 | TGDWVAKWTSTSMP | 4 | VAKWTSTSM | 0.980 | 4O0H_PDBID | 0.244537 | 4.07 | NA | <=WB |
| 273 | DRB1_1501 | GDWVAKWTSTSMPW | 3 | VAKWTSTSM | 1.000 | 4O0H_PDBID | 0.291959 | 3.40 | NA | <=WB |
| 1 | DRB1_1501 | HMNPIVVVHGGGAGP | 4 | IVVVHGGGA | 0.987 | 4O0H_PDBID | 0.624314 | 1.10 | NA | <=WB |
| 2 | DRB1_1501 | MNPIVVVHGGGAGPI | 3 | IVVVHGGGA | 0.947 | 4O0H_PDBID | 0.624292 | 1.10 | NA | <=WB |
| 3 | DRB1_1501 | NPIVVVHGGGAGPIS | 2 | IVVVHGGGA | 0.827 | 4O0H_PDBID | 0.469541 | 1.86 | NA | <=WB |
| 35 | DRB1_1501 | GYGILREGGSAVDAV | 3 | ILREGGSAV | 0.993 | 4O0H_PDBID | 0.210121 | 4.77 | NA | <=WB |
| 256 | DRB1_1501 | MKSRVKGLGGLIVVS | 4 | VKGLGGLIV | 1.000 | 4O0H_PDBID | 0.283149 | 3.51 | NA | <=WB |
| 257 | DRB1_1501 | KSRVKGLGGLIVVSK | 3 | VKGLGGLIV | 1.000 | 4O0H_PDBID | 0.427525 | 2.15 | NA | <=WB |
| 271 | DRB1_1501 | KTGDWVAKWTSTSMP | 5 | VAKWTSTSM | 0.960 | 4O0H_PDBID | 0.209546 | 4.78 | NA | <=WB |
| 272 | DRB1_1501 | TGDWVAKWTSTSMPW | 4 | VAKWTSTSM | 0.993 | 4O0H_PDBID | 0.290189 | 3.42 | NA | <=WB |
| 273 | DRB1_1501 | GDWVAKWTSTSMPWA | 3 | VAKWTSTSM | 1.000 | 4O0H_PDBID | 0.395839 | 2.39 | NA | <=WB |

Table S3 Epitope density of the 3ECA ,4O0H and 3ECA Humanized Chimeric Protein proteins. To calculate the relative frequency, the formula fi = ni ⁄ N was used, where ni is the number of predicted immunogenic epitopes, and N is the total number of epitopes determined by the program (immunogenic and nonimmunogenic). The epitope density of each protein was determined for the alleles HLA-DRB1*01:01, HLA-DRB1*03:01, HLA-DRB1*04:01, HLA-DRB1*07:01, HLADRB1*08:01, HLA-DRB1*11:01, HLA-DRB1*13:01 and HLADRB1*15:01, which are reference alleles in the literature with a wide global frequency.

| | 3ECA Protien | | | 4O0H Protein | | |
|---|---|---|---|---|---|---|
| Allele | Total Immunogenic Epitopes (ni) | Total Epitopes (N) | fi | Total Immunogenic Epitopes (ni) | Total Epitopes (N) | fi |
| HLA-DRB1*01:01 | 71 | 2205 | 0.032 | 64 | 2086 | 0.031 |
| HLA-DRB1*03:01 | 33 | 2205 | 0.014 | 64 | 2086 | 0.031 |
| HLA-DRB1*04:01 | 94 | 2205 | 0.042 | 39 | 2086 | 0.019 |
| HLA-DRB1*07:01 | 162 | 2205 | 0.073 | 50 | 2086 | 0.024 |
| HLA-DRB1*08:01 | 33 | 2205 | 0.014 | 17 | 2086 | 0.008 |
| HLA-DRB1*11:01 | 36 | 2205 | 0.016 | 11 | 2086 | 0.005 |
| HLA-DRB1*13:01 | 31 | 2205 | 0.014 | 29 | 2086 | 0.014 |
| HLA-DRB1*15:01 | 72 | 2205 | 0.032 | 21 | 2086 | 0.010 |

| | 3ECA Humanized Chimera Protein | | |
|---|---|---|---|
| Allele | Total Immunogenic Epitopes (ni) | Total Epitopes (N) | fi |
| HLA-DRB1*01:01 | 76 | 2205 | 0.034 |
| HLA-DRB1*03:01 | 40 | 2205 | 0.018 |
| HLA-DRB1*04:01 | 105 | 2205 | 0.048 |
| HLA-DRB1*07:01 | 148 | 2205 | 0.067 |
| HLA-DRB1*08:01 | 22 | 2205 | 0.010 |
| HLA-DRB1*11:01 | 36 | 2205 | 0.016 |
| HLA-DRB1*13:01 | 28 | 2205 | 0.013 |
| HLA-DRB1*15:01 | 53 | 2205 | 0.024 |

**Table** S4.**Affinity results of 7 ligands evaluated in retrospective docking with Chimeric Asparaginase.**
**The asparagine value appears in blue.**
**The ligands were chosen at random, conserving glutamine and asparagine: glutamine(5961), ATP(5957), leucine(6106) ,phenylalanine(994), tyrosine(6075), tryptophan(6305), asparagine(6267).**

| Ligand | Binding Affinity | rmsd/ub | rmsd/lb |
|---|---|---|---|
| recpetor6_6106 | -4.7 | 0.0 | 0.0 |
| recpetor6_6106 | -4.6 | 14.218 | 13.704 |
| recpetor6_6106 | -4.6 | 24.905 | 24.07 |
| recpetor6_6106 | -4.6 | 12.513 | 11.765 |
| recpetor6_6106 | -4.5 | 25.568 | 24.34 |
| recpetor6_6106 | -4.5 | 26.509 | 25.414 |
| recpetor6_6106 | -4.5 | 33.083 | 31.617 |
| recpetor6_6106 | -4.5 | 20.956 | 19.886 |
| recpetor6_6106 | -4.4 | 27.881 | 26.661 |
| recpetor6_5957 | -7.9 | 0.0 | 0.0 |
| recpetor6_5957 | -7.6 | 15.225 | 12.788 |
| recpetor6_5957 | -7.6 | 14.934 | 12.216 |
| recpetor6_5957 | -7.4 | 15.815 | 13.528 |
| recpetor6_5957 | -7.2 | 5.255 | 3.033 |
| recpetor6_5957 | -7.2 | 15.617 | 13.358 |
| recpetor6_5957 | -7.1 | 14.856 | 12.146 |
| recpetor6_5957 | -7.1 | 6.886 | 4.195 |
| recpetor6_5957 | -7.1 | 5.725 | 4.259 |
| recpetor6_6305 | -6.7 | 0.0 | 0.0 |
| recpetor6_6305 | -6.6 | 11.988 | 10.524 |
| recpetor6_6305 | -6.6 | 5.838 | 4.309 |
| recpetor6_6305 | -6.4 | 2.251 | 1.688 |
| recpetor6_6305 | -6.4 | 17.587 | 16.113 |
| recpetor6_6305 | -6.4 | 24.662 | 23.536 |
| recpetor6_6305 | -6.3 | 18.273 | 16.779 |
| recpetor6_6305 | -6.2 | 2.961 | 2.318 |
| recpetor6_6305 | -6.1 | 30.02 | 28.437 |
| recpetor6_6057 | -6.5 | 0.0 | 0.0 |
| recpetor6_6057 | -6.3 | 38.012 | 36.756 |
| recpetor6_6057 | -6.2 | 23.791 | 22.529 |
| recpetor6_6057 | -6.2 | 37.511 | 36.288 |
| recpetor6_6057 | -6.2 | 38.026 | 37.053 |
| recpetor6_6057 | -6.2 | 13.536 | 12.375 |
| recpetor6_6057 | -6.1 | 16.368 | 14.91 |
| recpetor6_6057 | -6.1 | 25.283 | 24.11 |
| recpetor6_6057 | -6.1 | 37.08 | 36.074 |
| recpetor6_994 | -6.0 | 0.0 | 0.0 |
| recpetor6_994 | -5.9 | 12.264 | 11.3 |
| recpetor6_994 | -5.9 | 25.553 | 24.613 |
| recpetor6_994 | -5.9 | 19.581 | 18.578 |
| recpetor6_994 | -5.8 | 16.055 | 14.762 |
| recpetor6_994 | -5.8 | 5.978 | 4.597 |
| recpetor6_994 | -5.7 | 17.316 | 16.249 |
| recpetor6_994 | -5.6 | 19.232 | 17.884 |
| recpetor6_994 | -5.6 | 29.337 | 27.709 |

| | | | |
|---|---|---|---|
| recpetor6_5961 | -5.7 | 0.0 | 0.0 |
| recpetor6_5961 | -5.1 | 4.324 | 2.047 |
| recpetor6_5961 | -5.0 | 16.51 | 15.663 |
| recpetor6_5961 | -5.0 | 17.922 | 17.108 |
| recpetor6_5961 | -4.9 | 29.228 | 28.392 |
| recpetor6_5961 | -4.9 | 39.943 | 39.208 |
| recpetor6_5961 | -4.7 | 22.375 | 21.551 |
| recpetor6_5961 | -4.7 | 23.583 | 22.604 |
| recpetor6_5961 | -4.7 | 15.388 | 14.57 |
| recpetor6_6267 | -5.9 | 0.0 | 0.0 |
| recpetor6_6267 | -5.9 | 28.312 | 26.825 |
| recpetor6_6267 | -5.8 | 16.66 | 15.655 |
| recpetor6_6267 | -5.7 | 16.643 | 15.667 |
| recpetor6_6267 | -5.7 | 28.275 | 26.577 |
| recpetor6_6267 | -5.7 | 3.793 | 2.024 |
| recpetor6_6267 | -5.5 | 11.221 | 10.456 |
| recpetor6_6267 | -5.5 | 7.378 | 6.081 |
| recpetor6_6267 | -5.4 | 11.102 | 10.313 |

Table S5. Docking of the native enzyme 3ECA with the ligand asparagine. Affinity Results

| Ligand | Binding Affinity | rmsd/ub | rmsd/lb |
|---|---|---|---|
| 3eca_cleaned_6267_uff_E=44.03 | -5.6 | 0.0 | 0.0 |
| 3eca_cleaned_6267_uff_E=44.03 | -5.5 | 3.805 | 1.551 |
| 3eca_cleaned_6267_uff_E=44.03 | -5.5 | 29.988 | 28.166 |
| 3eca_cleaned_6267_uff_E=44.03 | -5.4 | 29.74 | 28.281 |
| 3eca_cleaned_6267_uff_E=44.03 | -5.4 | 30.121 | 28.93 |
| 3eca_cleaned_6267_uff_E=44.03 | -5.4 | 6.634 | 6.056 |
| 3eca_cleaned_6267_uff_E=44.03 | -5.2 | 4.03 | 2.14 |
| 3eca_cleaned_6267_uff_E=44.03 | -5.2 | 6.861 | 5.852 |
| 3eca_cleaned_6267_uff_E=44.03 | -5.2 | 7.076 | 6.322 |
